# Palmitate promotes liver metastases by decreasing neutrophil antitumour behaviour

**DOI:** 10.1038/s42255-026-01582-0

**Published:** 2026-08-05

**Authors:** Anke Vandekeere, Juan Fernández-García, Yiming Peng-Winkler, Miguel Martín Pérez, Ming Liu, Sara Isabel Fernandes, Janine Theile, Philipp Willnow, Sanne Verheul, Gitte Zels, Anirudh Pabba, Christine Desmedt, Cornelis Verhoef, Patricia Altea-Manzano, Salvador Aznar Benitah, Ilaria Elia, Ilaria Malanchi, Sarah-Maria Fendt

**Affiliations:** 1 Laboratory for Tumor Evolution and Metastasis, VIB-KU Leuven Center for Cancer Biology, VIB, Herestraat 49, 3000 Leuven, Belgium; 2 Laboratory for Tumor Evolution and Metastasis, Department of Oncology, KU Leuven and Leuven Cancer Institute (LKI), Herestraat 49, 3000 Leuven, Belgium; 3 Department of Gastroenterology, Hepatology and Infectious Diseases, University Hospital Dusseldorf, Dusseldorf, Germany; 4 Institute for Research in Biomedicine (IRB Barcelona), The Barcelona Institute of Science and Technology (BIST), 08028 Barcelona, Spain; 5 Department of Cell Biology, Physiology and Immunology, University of Barcelona, 08028 Barcelona, Spain; 6 Serra Húnter Programme (PSH), Departament de Recerca i Universitats de la Generalitat de Catalunya, 08003 Barcelona, Spain; 7 Department of Surgery, Erasmus MC Cancer Institute, Rotterdam, The Netherlands; 8 Laboratory for Translational Breast Cancer Research, Department of Oncology, KU Leuven, Leuven, Belgium; 9 Catalan Institution for Research and Advanced Studies (ICREA), 08010 Barcelona, Spain; 10 Department of Cellular and Molecular Medicine, KU Leuven, 3000 Leuven, Belgium; 11 Tumour Host Interaction laboratory, The Francis Crick Institute, 1 Midland Road, NW1 1AT London; 12 Laboratory of Metabolic Regulation and Signaling in Cancer, Andalusian Molecular Biology and Regenerative Medicine Centre (CABIMER)- University of Seville - CSIC- University Pablo de Olavide, 41092, Sevilla, Spain

## Abstract

Liver metastases are frequent and challenging to treat owing to the liver’s metabolically active and immune-tolerant environment. However, how cancer cells exploit nutrient availability in the liver to evade immune surveillance remains unknown. Here, we show that cancer cells use the palmitate availability in the liver to impair the neutrophil antitumor function. Mechanistically, we find that breast and colorectal cancer cells metastasizing to the liver, but not the lung, require the palmitoyltransferase 17 (DHHC17, gene name *ZDHHC17*) to stabilize laminin-511 enabling its secretion. In turn, neutrophils in the liver metastasis environment respond to laminin-511 by decreasing their cancer cell-killing capacity. Consistently, silencing *ZDHHC17* in cancer cells decreases liver metastases only in the presence of neutrophils, while metastasis growth is restored in *ZDHHC17*-silenced metastases upon injection of laminin-511 or inhibition of neutrophil degranulation. Taken together, we find that liver palmitate not only supports tumour intrinsic processes but also enables immune evasion.

## Introduction

The liver and its metabolism play a key role in maintaining a healthy whole-body physiology^1^. They preserve blood glucose homeostasis, produce bile acid for dietary fat digestion, synthesize lipids and proteins, detoxify various intrinsic and extrinsic molecules, and control immune functions such as immune tolerance to help prevent autoimmunity^1,2^. The liver is susceptible to various diseases including primary tumours and metastases from several cancer types such as breast and colorectal cancer^3^. However, liver metastases across cancer types show a poor response to current immunotherapy options, highlighting a significant clinical challenge^4,5^.

The unique metabolic environment of the liver can enhance the fitness of cancer cells within liver metastases^6^. The high glucose availability fuels glycolysis, supporting macromolecule synthesis and allowing cancer cells to adapt to low oxygenated regions of the liver^7,8^. Additionally, liver metastases synthesize creatine and increase their energy metabolism flexibility^9, 10^. Moreover, the liver is inherently rich in lipids and fatty acids such as palmitate and oleate^11^. Cancer cells can exploit these lipids for membrane synthesis^12^, energy production through oxidation^13^, and generation of precursors for post-translational modifications and signalling molecules^14,15^. Dietary imbalances from carbohydrate and/or fat-rich diets can further increase the hepatic lipid availability and promote metastases across multiple cancer types^11,16,17^. Thus, particular nutrients including lipids support a pro-metastatic metabolism in the liver.

The metabolic environment of the liver also fosters a stromal cell niche supportive of liver metastases. For example, bile acids and their derivatives produced by the liver can impair the antitumor function of T cells in hepatocellular carcinoma models^18^. Moreover, elevated lipid levels associated with diet or obesity can impair CD8^+^ T cell and dendritic cell function^19^, and induce an inflammatory phenotype in liver endothelial cells to promote the recruitment of protumour immune cells to the liver^20,21^. Additionally, tumour cells in the liver can themselves exacerbate this immunosuppressive environment by secreting long-chain fatty acids (LCFAs) to promote the polarization of macrophages toward an immunosuppressive phenotype^22^. Thus, the liver metabolites, and in particular lipids, can directly affect stromal and immune cell behaviour, weakening their ability to act as a barrier against tumour progression.

However, despite these findings it remains largely elusive whether cancer cells actively use environmental lipids from the liver to hamper antitumor immune cells within the liver metastasis environment. We discovered that the activity of palmitoyltransferase (DHHC) 17 and subsequent laminin-511 secretion by breast and colorectal cancer cells impair the antitumour behaviour of neutrophils in the liver metastasis environment.

## Results

### DHHC17 is required for liver but not lung metastasis growth

The expression landscape of palmitoyltransferases, which post-translationally attach palmitate to proteins, is strongly influenced by the cancer type^23^. Yet, little is known about whether their importance and role change when cancer cells of the same origin grow in different organs, such as during metastasis growth.

**Fig 1:**
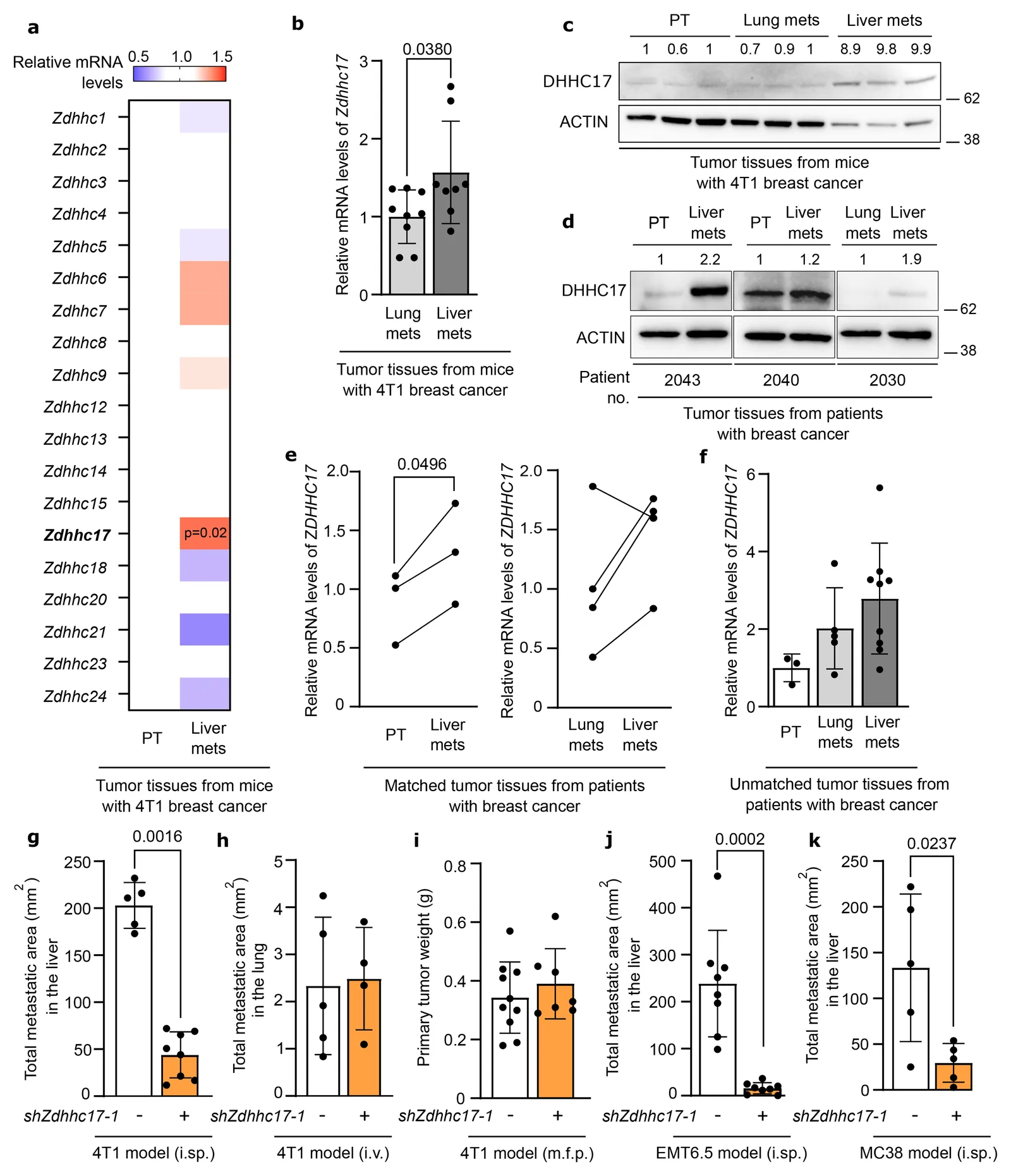
DHHC17 is required for liver metastasis growth a. Relative mRNA levels of detected palmitoyl transferases (*Zdhhc)* in primary tumors from mice injected with 4T1 cells in the mammary fat pad and in liver metastases from mice *intrasplenically* injected with 4T1 cells (n=4 primary tumors, n=6 liver metastases). Data are normalized to the primary tumors. Two-sided unpaired t test. b. Relative mRNA levels of *Zdhhc17* in lung and liver metastases from mice *hydrodynamically* injected with 4T1 cells (n=9 lung metastases, n=8 liver metastases). Data are normalized to the lung metastases. Two-sided unpaired t-test. c. Protein levels of DHHC17 in primary tumors (PT), lung and liver metastases from the 4T1 mouse model (n=3 mice). Numbers above the bands indicate the relative band intensity after normalization to β-ACTIN. d. Protein levels of DHHC17 in matched primary tumors (PT), lung and liver metastases from tissues of human breast cancer patients (UPTIDER program) (n=3 individuals). Numbers above the bands indicate the relative band intensity after normalization to β-ACTIN. e. Left: Relative mRNA levels of *ZDHHC17* in primary tumor (PT) and liver metastases from matched tissues of human breast cancer patients (UPTIDER program) (n=3 primary tumors and n=3 liver metastases). Data are normalized to each matched primary tumor. Right: Relative mRNA levels of ZDHHC17 in lung and liver metastases from matched tissues of human breast cancer patients (UPTIDER program) (n=4 lung metastases, n=4 liver metastases). Data are normalized to each matched primary tumor. Two-sided paired t-test. f. Relative mRNA levels of *ZDHHC17* in primary tumor (PT), lung and liver metastases from unmatched tissues of human breast cancer patients (UPTIDER program) (n=3 primary tumors, n=5 lung metastases and n=9 liver metastases). Data are normalized to primary tumors. g. Total metastatic area in livers of mice *intrasplenically* injected with shSCR (control) or sh*Zdhhc17-1* 4T1 cells (n=5 for control, n=8 for sh*Zdhhc17*). Two-sided unpaired Mann-Whitney t test. Outliers were detected with Grubb’s test and removed. Representative H&E images are shown in Extended Data Fig. 9a. h. Total metastatic area in lung of mice *intravenously* injected with shSCR2 (control) or sh*Zdhhc17-1* 4T1 cells (n=5). i. Primary tumor weight (g) 21 days after mice were inoculated in the mammary fat pad with shSCR or sh*Zdhhc17-1* 4T1 cells (n=10 for shSCR and n=7 for sh*Zdhhc17*) j. Total metastatic area in livers of mice *intrasplenically* injected with shSCR (control) or sh*Zdhhc17-1* EMT6.5 cells (n=8). Two-sided unpaired Mann-Whitney t test. Representative H&E images are shown in Extended Data Fig. 9b. k. Total metastatic area in livers of mice *intrasplenically* injected with shSCR (control) or sh*Zdhhc17-1* MC38 cells (n=5). Two-sided unpaired Mann-Whitney t test. Representative H&E images are shown in Extended Data Fig. 9c. Data are mean ± s.d. unless indicated otherwise.

We analysed the expression of 24 palmitoyltransferases in metastases compared to primary breast tumours of mice. For this, we injected mouse 4T1 breast cancer cells *intrasplenically* into Balb/c mice, which results in liver metastases, and into the mammary fat pad, resulting in primary breast tumours. We observed that *Zdhhc17* (protein name DHHC17) expression significantly increased in liver metastases compared to primary tumours (Fig. 1a). Subsequently, we assessed whether the increase in *Zdhhc17* expression in metastases is related to the liver environment. Therefore, we compared liver and lung metastases by injecting mouse 4T1 breast cancer cells *hydrodynamically* into Balb/c mice, which leads to metastases in both organs within the same mouse. We found that liver metastases showed increased *Zdhhc17* mRNA levels compared to lung metastases, as well as increased protein levels compared to both lung metastases and primary tumours (Fig. 1b-c).

Next, we focused on DHHC17 and asked whether it is differentially expressed in liver compared to lung metastases or primary breast tumours from patients. Specifically, we assessed the mRNA and protein levels of DHHC17 in liver and lung metastasis tissues and/or primary breast tumours of patients with advanced breast cancer (mRNA: 3 primary tumour, 5 lung metastasis, 8 liver metastasis; protein: 2 primary tumour, 1 lung metastasis, 3 liver metastasis) collected *postmortem* via the UPTIDER tissue donation program (KU/UZ Leuven Program for Postmortem Tissue Donation to Enhance Research; Leuven, Belgium, NCT04531696) (Supplementary Table 1)^24^. Consistent with the mouse data, we found that liver metastases showed increased mRNA and protein levels of DHHC17 compared to primary breast tumours and/or lung metastases of patients (Fig. 1d-f). Based on these data, we concluded that the DHHC17 expression landscape changes during cancer progression, with liver metastases having the highest expression compared to lung metastases and primary tumours of breast cancer patients.

**Extended Data Fig. 1:**
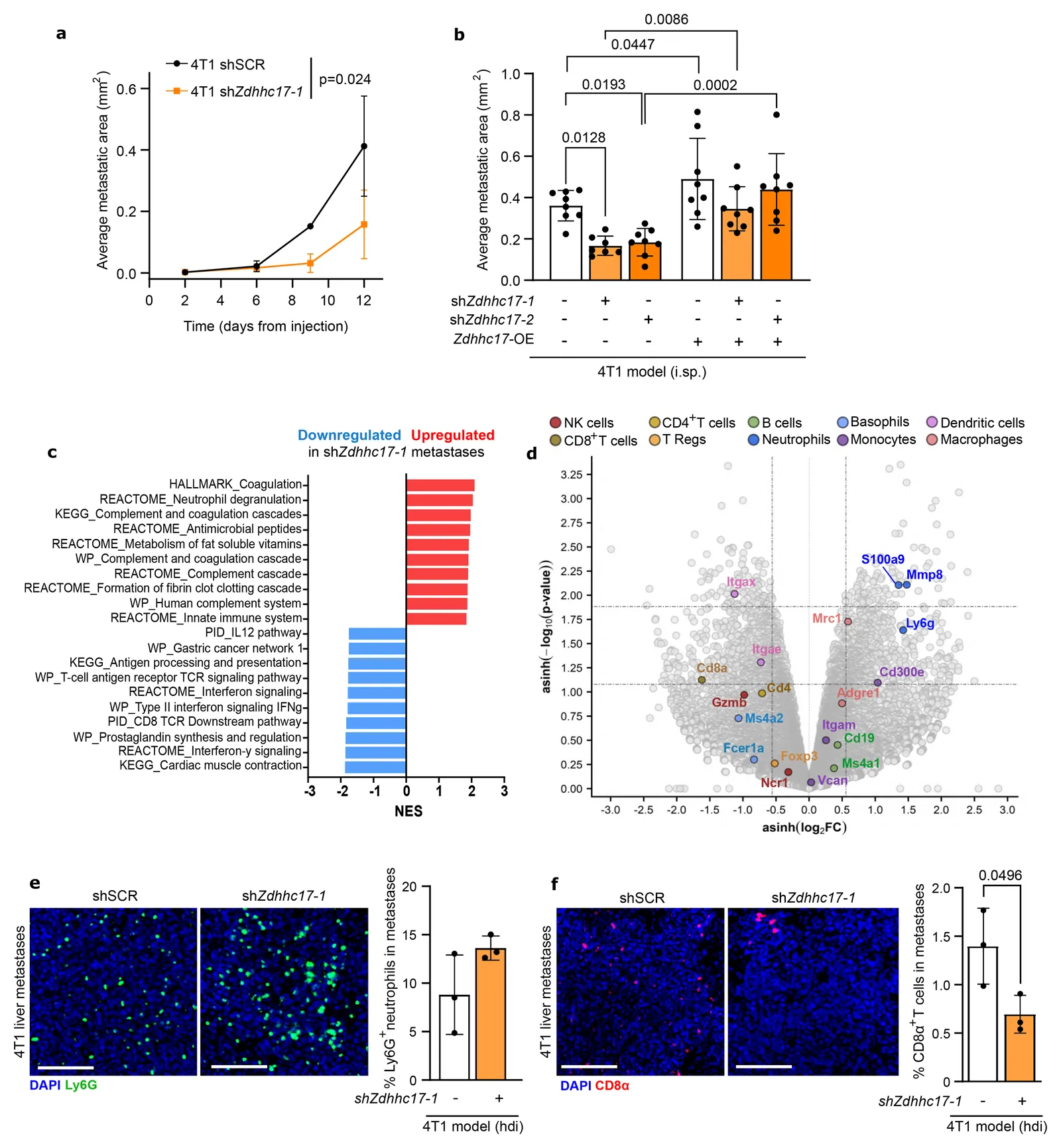
Immune cell infiltration in *Zdhhc17-*silenced liver metastases a. Average metastatic area in livers of mice at day 2, 4, 5, 9 and 12 after intrasplenic injection with shSCR or *shZdhhc17-1* 4T1 cells (n=2 at day 2, n=3 at each other timepoint for both shSCR and *shZdhhc17-1*). Statistical analysis based on one-sided F-Test on 3rd degree polynomials, with F-Test p-value shown in graph. The intercepts of the polynomials (values at day 0) were manually set to 0 by virtue of the experimental design (no metastases present). b. Average metastatic area in livers of mice *intrasplenically* injected with shSCR, *shZdhhc17-1* or *shZdhhc17-2* 4T1 cells, with/without overexpression of *Zdhhc17* (n=7 for *shZdhhc17-1*, n=8 others). 2-way ANOVA. Representative H&E images are shown in Extended Data Fig. 9q. c. GSEA-derived normalized enrichment scores (NES) for the top 10 enriched/depleted genesets in sh*Zdhhc17-1* relative to shSCR2 4T1 liver metastases based on the bulk RNA-sequencing (n=3 mice). d. Differential expression analysis results for sh*Zdhhc17*-1 versus shSCR2 4T1 liver metastases (n=3 mice), based on bulk RNA-sequencing. Marker genes of immune populations are highlighted, indicated by colors. DESeq2-derived log_2_ fold changes (FC) and negative log_10_-transformed *P*-values are indicated in the x and y axes, respectively, and are subject to an inverse hyperbolic sine (asinh) transformation forvisualization. The horizontal dashed lines represent (from bottom to top) raw and FDR-adjusted *P*-values of 0.05, while the vertical ones represent absolute fold-changes of 1.5. *P-*values based on two-sided DESeq2’s Wald test implementation, with FDR adjustment based on the Benjamini-Hochberg (BH) approach. e. Left: Representative staining for Ly6G^+^ cells in shSCR and sh*Zdhhc17-1* liver metastases of 4T1 cells, at day 10 after hydrodynamic injection. Blue, DAPI nuclear staining; green, Ly6G. Scale bar 100µm. Right: Percent Ly6G^+^ neutrophils in the shSCR and sh*Zdhhc17-1* liver metastases of 4T1 cells at day 10 after hydrodynamic injection (n=3 mice). For the analysis, similarly sized metastases were taken. Unpaired two-tailed t-test. f. Left: Representative staining for CD8^+^ T cells in shSCR and sh*Zdhhc17-1* liver metastases of 4T1 cells, at day 10 after hydrodynamic injection. Blue, DAPI nuclear staining; red, CD8a. Scale bar 100µm. Left: Percent CD8^+^ T cells in the shSCR and sh*Zdhhc17-1* liver metastases of 4T1 cells at day 10 after hydrodynamic injection (n=3 mice). For the analysis, similarly sized metastases were taken. Unpaired two-tailed t-test. Data are mean ± s.d. unless indicated otherwise.

Next, we asked whether DHHC17 is essential for the growth of liver metastases. For this, we silenced *Zdhhc17* in 4T1 breast cancer cells and initiated liver and lung metastases by *intrasplenic* or *intravenous* injection, respectively, as well as primary tumour growth by mammary fat pad injection. In line with the expression profiles, we observed that *Zdhhc17* silencing impaired liver but not lung metastases or primary tumour growth (Fig. 1g-i). Importantly, the slower growth of 4T1 liver metastases upon *Zdhhc17* silencing (Extended Data Fig. 1a) was rescued by the overexpression of shRNA resistant *Zdhhc17* (Extended Data Fig. 1b) and was further confirmed in the EMT6.5 breast cancer and the MC38 colorectal cancer models (Fig. 1j-k). Based on these data, we concluded that DHHC17 is specifically required for effective liver metastasis growth in breast and colorectal cancer mouse models.

### Neutrophils mediate the decreased metastasis growth upon loss of DHHC17 in cancer cells

**Extended Data Fig. 2:**
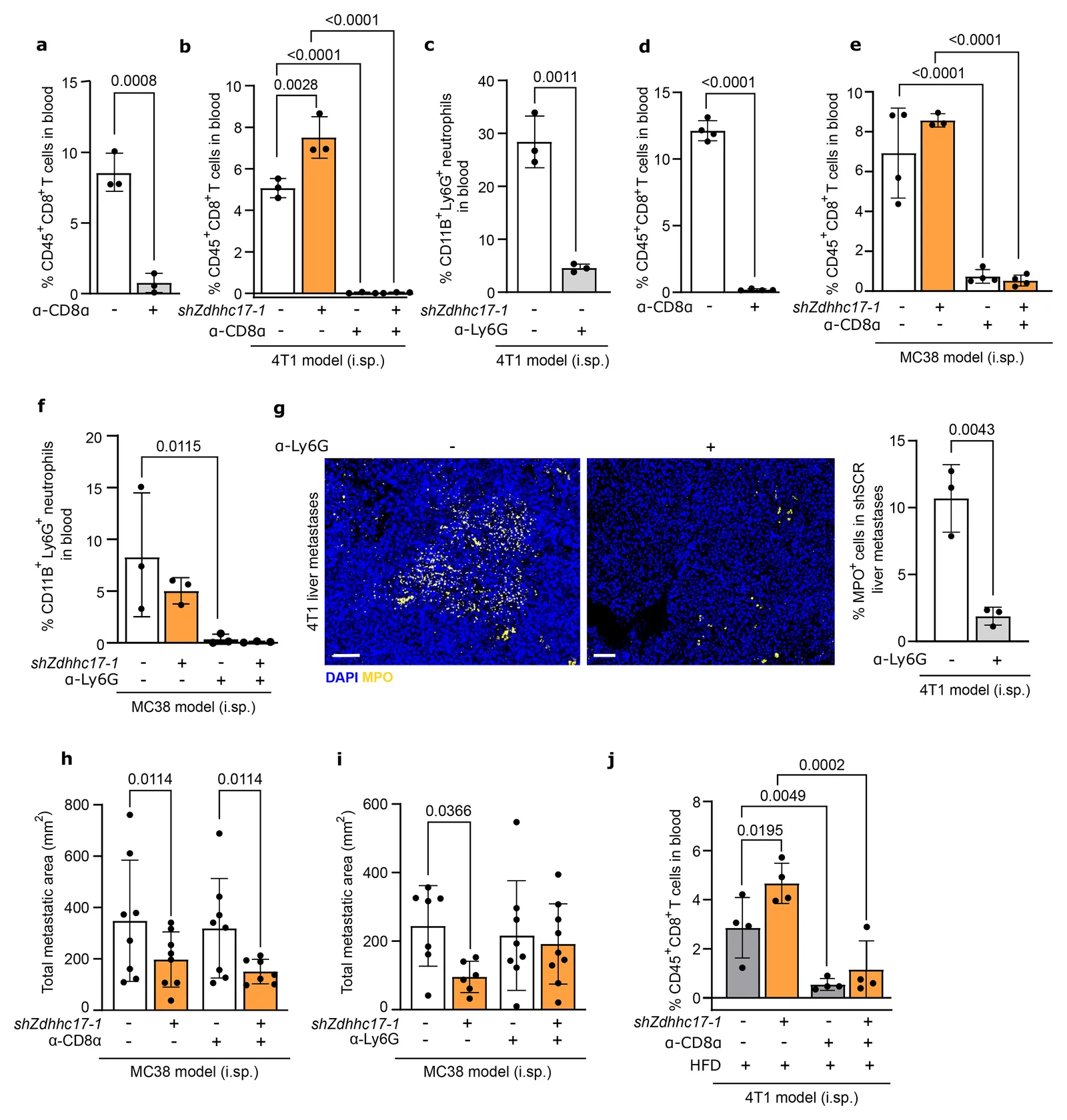
Efficiency of CD8^+^ T cells and neutrophils depletion a. Percent of blood circulating CD45^+^ CD8a^+^ T cells in mice at 3 days post IP injection of IgG2a or α-CD8α depleting antibody (n=3) (day of 4T1 cancer cell injection). Unpaired two-tailed t-test. b. Percent of blood circulating CD45^+^ CD8^+^ T cells in mice on day 9 after cancer cell injection (4T1 shSCR or sh*Zdhhc17-1*) and treated with IgG2a antibody or α-CD8a depleting antibody (n=3 each group). 2-way ANOVA. c. Percent of blood circulating CD11B^+^ Ly6G^+^ neutrophils in mice at day 12 after shSCR 4T1 cancer cell injection and treated with IgG2a or α-Ly6G depleting antibody injection (n=3). Unpaired two-tailed t-test. d. Percent of blood circulating CD45^+^ CD8^+^ T cells in mice at 3 days post IP injection of IgG2aor α-CD8α depleting antibody (n=3) (day of MC38 cancer cell injection). Unpaired two-tailed t-test. e. Percent of blood circulating CD45^+^ CD8^+^ T cells in mice on day 9 after cancer cell injection (MC38 shSCR or sh*Zdhhc17-1*) and treated with IgG2a antibody or α-CD8a depleting antibody (n=3 for sh*Zdhhc17-1* with IgG2a, n=4 for all other groups). 2-way ANOVA. f. Percent of blood circulating CD11B^+^ Ly6G^+^ neutrophils in mice at day 10 after shSCR and sh*Zdhhc17-1* MC38 cancer cell injection and treated with IgG2a control antibody or α - Ly6G depleting antibody injection (n=3 each group). 2-way ANOVA. g. Left: Representative fluorescent staining of MPO^+^ neutrophils in 4T1 shSCR liver metastases and treated with IgG2a or α-Ly6G depleting antibody for 10 days. Right: Percent MPO^+^ cells in 4T1 shSCR liver metastases upon treatment with control or α-Ly6G depleting antibody for 10 days (n=3 mice). Unpaired two-tailed t-test. h. Total metastatic area in livers of mice *intrasplenically* injected with shSCR or sh*Zdhhc17-1* MC38 cells in the presence (IgG2a) (n=8) or absence of CD8α T cells (α-CD8α) (n=8 for shSCR and n=7 for sh*Zdhhc17-1*). Depletion of T cells was initiated 3 days prior cancer cell injection. 2-way ANOVA. Representative H&E images are shown in Extended Data Fig. 9k. i. Total metastatic area in livers of male mice *intrasplenically* injected with shSCR or sh*Zdhhc17-1* MC38 cells and treated after 24h with a Ly6G targeting (α-Ly6G) (n=7 for shSCR and n=6 for sh*Zdhhc17-1*) or IgG2a antibody (n=8 shSCR and n=9 sh*Zdhhc17*). To enhance efficiency of the depletion, an anti-rat kappa immunoglobulin was given bidaily. 2-way ANOVA. Representative H&E images are shown in Extended Data Fig. 9j. j. Percent of blood circulating CD45^+^ CD8^+^ T cells in mice on day 13 after cancer cell injection (4T1 shSCR or sh*Zdhhc17-1*), fed with HFD and treated with IgG2aantibody or α-CD8a depleting antibody (n=4). 2-way ANOVA. Data are mean ± s.d. unless indicated otherwise.

**Fig 2:**
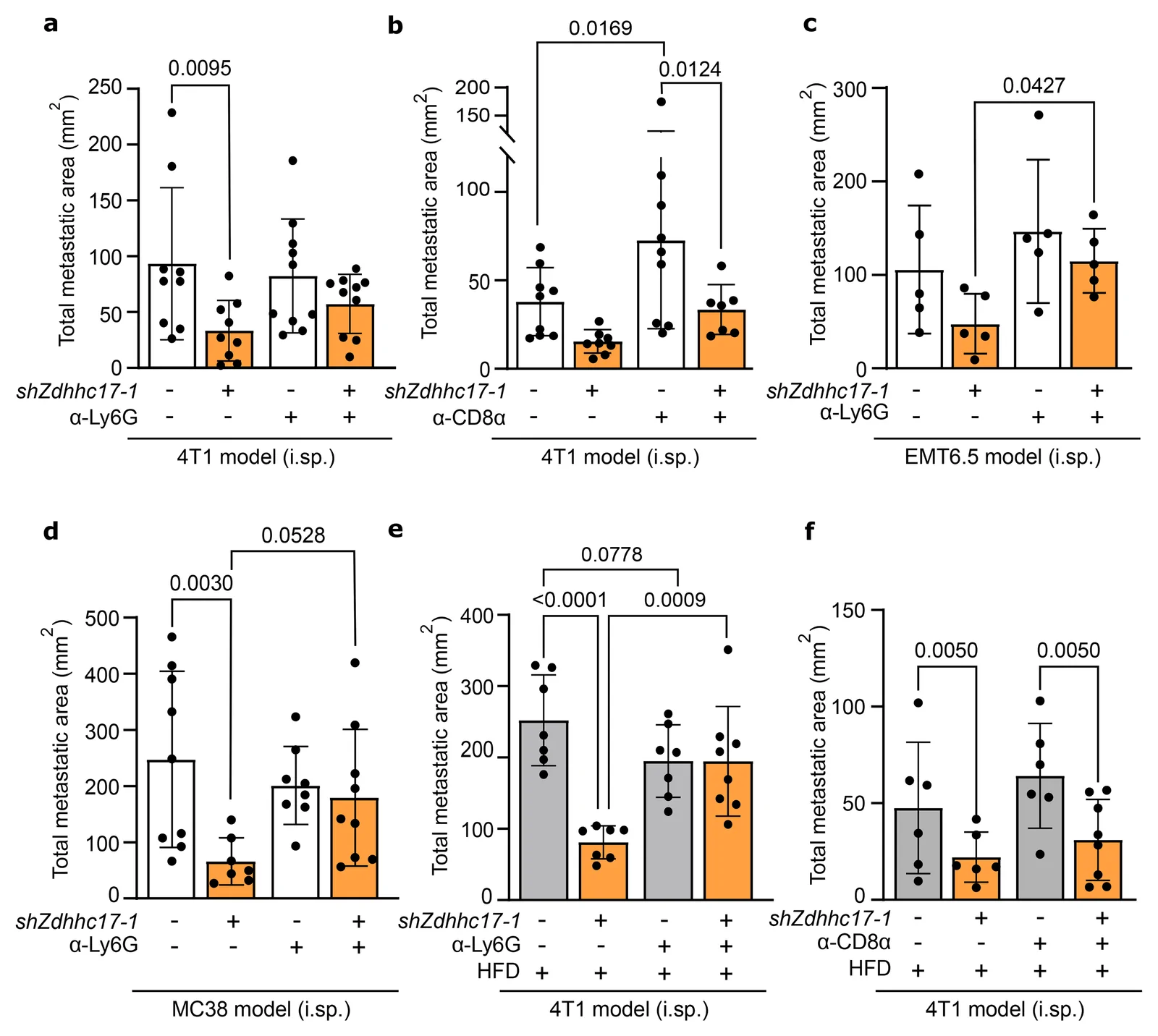
Decreased liver metastasis growth upon loss of DHHC17 in cancer cells is neutrophil dependent a. Total metastatic area in livers of mice *intrasplenically* injected with shSCR or sh*Zdhhc17-1* 4T1 cells and treated after 24h with a Ly6G targeting (α-Ly6G) (n=9) or control antibody (IgG2a) (n=10). 2-way ANOVA. Representative H&E images are shown in Extended Data Fig. 9f. b. Total metastatic area in livers of mice *intrasplenically* injected with shSCR or sh*Zdhhc17-1* 4T1 cells in the presence (IgG2a) (n=9 for shSCR, n=8 for sh*Zdhhc17*) or absence of CD8^+^ T cells (α-CD8α) (n=9 for shSCR, n=7 for sh*Zdhhc17*). Depletion of T cells was initiated 3 days prior cancer cell injection. 2-way ANOVA. Representative H&E images are shown in Extended Data Fig. 9g. c. Total metastatic area in livers of mice *intrasplenically* injected with shSCR or sh*Zdhhc17-1* EMT6.5 cells and treated after 24h with a Ly6G targeting (α-Ly6G) or control antibody (IgG2a) (n=5). 2-way ANOVA with Uncorrected Fisher’s LSD. Representative H&E images are shown in Extended Data Fig. 9h. d. Total metastatic area in livers of female mice *intrasplenically* injected with shSCR or sh*Zdhhc17-1* MC38 cells and treated after 24h with a Ly6G targeting (α-Ly6G) (n=9 for shSCR, n=7 for sh*Zdhhc17*) or control antibody (IgG2a) (n=8 shSCR, n=9 sh*Zdhhc17*). To enhance efficiency of the depletion, an anti-rat kappa immunoglobulin was given bidaily. Outliers were detected with Grubb’s test and removed. 2-way ANOVA. Representative H&E images are shown in Extended Data Fig. 9i. e. Total metastatic area in livers of mice fed with HFD for 16 weeks and *intrasplenically* injected with shSCR or sh*Zdhhc17-1* 4T1 cells and treated after 24h with a Ly6G targeting (α-Ly6G) or control antibody (IgG2a) (n=7). 2-way ANOVA. Representative H&E images are shown in Extended Data Fig. 9l. f. Total metastatic area in livers of mice fed with HFD for 16 weeks and *intrasplenically* injected with shSCR or *shZdhhc17-1* 4T1 cells in the presence (IgG2a) (n=6 for shSCR, n=6 for sh*Zdhhc17*) or absence of CD8^+^ T cells (α-CD8a) (n=6 for shSCR, n=8 for sh*Zdhhc17*). Depletion of T cells was initiated 3 days prior cancer cell injection. 2-way ANOVA. Representative H&E images are shown in Extended Data Fig. 9m Data are mean ± s.d. unless indicated otherwise.

To investigate how DHHC17 is required for the growth of cancer cells in the liver, we performed RNA-sequencing of resected liver metastases from *Zdhhc17-*silenced and control 4T1 cancer cells. Unexpectedly, we observed that some of the most altered gene sets upon *Zdhhc17* silencing were indicative of a shift in the immune compartment of these metastases (Extended Data Fig. 1c). Specifically, we observed that marker genes related to myeloid cells such as neutrophils (*Ly6G, S100a9, Mmp8*) were enriched in metastases from *Zdhhc17*-silenced cancer cells compared to control metastases, while a marker gene related to CD8^+^ T cells (*CD8α*) was decreased (Extended Data Fig. 1d). Immunohistochemistry (IHC) staining and FACS analysis for Ly6G and CD8α showed a slight, non-significant elevation in the percentage of Ly6G^+^ neutrophils and a significant but moderate reduction in the percentage of CD8^+^ T cells in the metastases, while blood CD8^+^ T cells increased upon *Zdhhc17* silencing in cancer cells (Extended Data Fig. 1e-f, Extended Data Fig. 2b). Subsequently, we investigated whether CD8^+^ T cells or neutrophils were required for the metastasis reduction we had observed upon *Zdhhc17* silencing in cancer cells. For this, we depleted either CD8^+^ T-cells or neutrophils in the control and sh*Zdhhc17* liver metastases models from 4T1, EMT6.5 and MC38 cell lines by injecting antibodies against CD8α (three days prior to cancer cell injection and repeated weekly after cancer cell injection)^25^ or Ly6G in female Balb/c mice and Ly6G combined with antibodies against rat kappa immunoglobulin in male and female C57BL/6 mice^26^ (daily after cancer cell injection)^27^. Effective depletion was validated by assessing the percentage of CD8^+^ T cells or Ly6G^+^ neutrophils in the blood of mice at the day of cancer cell injection as well as after injection (Extended Data Fig. 2a-f), and by immunofluorescent staining of myeloperoxidase (MPO) positive neutrophils in the liver upon sacrifice^26^ (Extended Data Fig. 2g). Strikingly, depletion of neutrophils but not CD8^+^ T cells abrogated the reduction in liver metastases upon *Zdhhc17* silencing in 4T1, EMT6.5 and MC38 cancer cells (Fig. 2a-d, Extended Data Fig. 2h-i). Consistent with palmitate being a substrate of DHHC17, the dependences of metastases growth on DHHC17 expression in cancer cells increased in mice fed a high-fat diet (HFD), but only in the presence of neutrophils (Fig. 2e), while CD8^+^ T cell depletion had no impact (Fig. 2f, Extended Data Fig. 2j). Based on these data, we concluded that neutrophils mediate the decrease in liver metastases growth upon loss of DHHC17 in cancer cells.

### Loss of DHHC17 in cancer cells decreases NETs

**Extended Data Fig 3:**
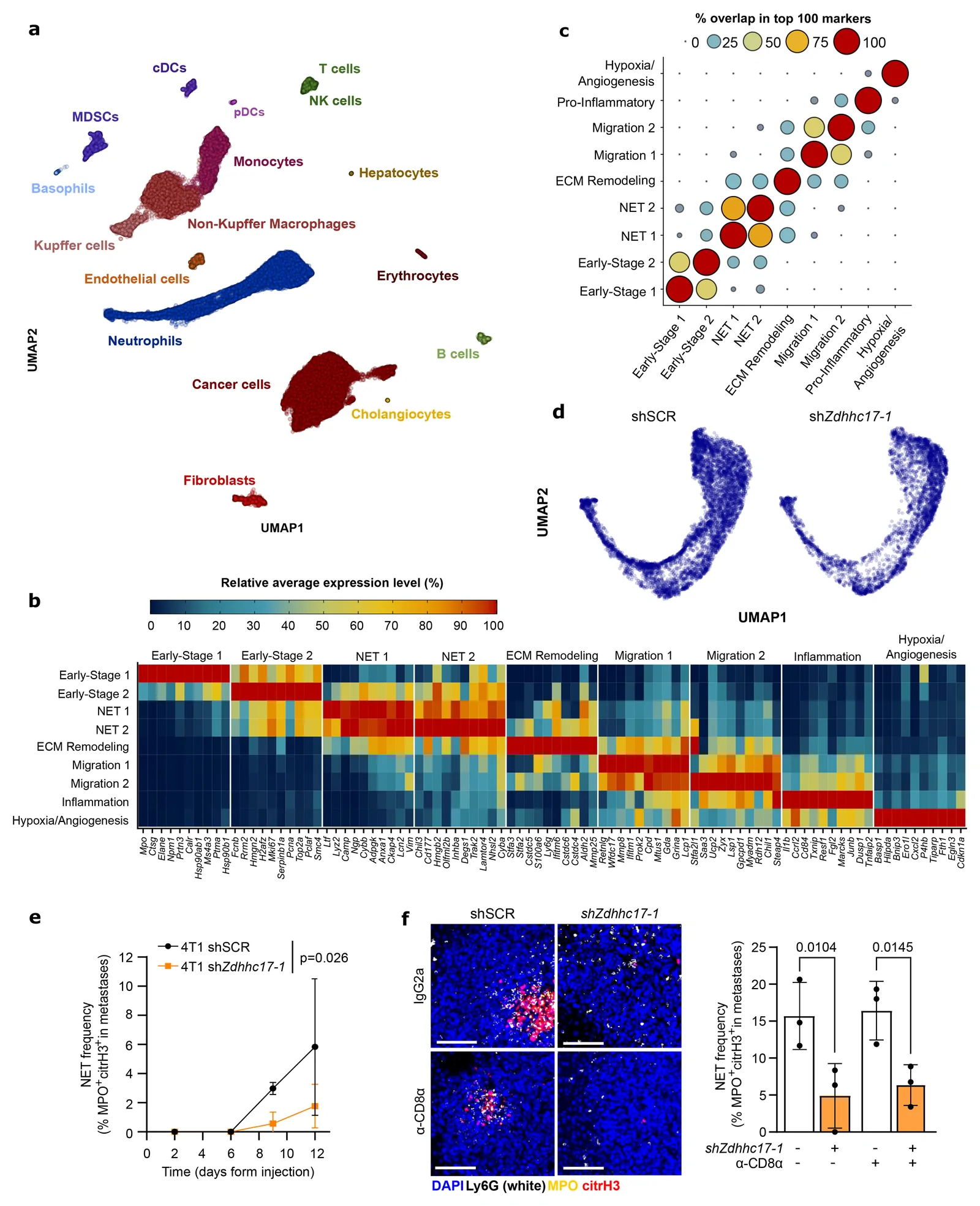
Characterization of neutrophil subpopulations using single cell RNA-sequencing a. Global UMAP including final cell-type annotations for all unambiguously annotated, non-multiplet cells in the scRNA-seq data from 4T1 shSCR and sh*Zdhhc17-1* liver metastases. b. Relative average expression profiles, across different neutrophil subsets (rows), for the top 10 marker genes (columns) identified for each of these subsets (column groups). Marker genes were uniquely assigned to the different subsets based on their expression levels and specificities relative to all other subsets. The color scale indicates the average expression level of each gene in each subset, normalized relative to the maximum average expression level of that gene across subsets. c. Percent overlap between the top 100 potential marker genes across different pairs of neutrophil subsets. The percent overlap is indicated by both the color scale and the dot sizes. d. Neutrophil-specific UMAP plot, showing separately the projections of neutrophils originating from either 4T1 shSCR (left) or sh*Zdhhc17-1* (right) liver metastases e. Percent NET release by mouse neutrophils in the metastases of 4T1 shSCR or sh*Zdhhc17*-1 at day 2, 6, 9 and 12 after cancer cell injection, based on double positive staining of MPO and citrH3 (n=2 at day 2, n=3 mice at all other timepoints). For the analysis only similar sized metastases were analyzed. Statistical analysis based on one-sided F-Test on 2nd degree polynomials, with F-Test p-value shown in graph. The intercepts of the polynomials (values at day 0) were manually set to 0 by virtue of the experimental design (no metastases present). f. Left: Representative fluorescent staining for NETs in shSCR and sh*Zdhhc17-1* 4T1 liver metastases in mice in presence/ absence of CD8^+^ T cells. Blue, DAPI nuclear staining; green, Ly6G; red, citrH3; yellow, MPO. Scale bar 100 µm. Right: Percent NET release by neutrophils in the metastases of 4T1 shSCR or sh*Zdhhc17-1* in presence/absence of CD8^+^ T-cells, based on double positive staining of MPO and citrH3 (n=3 mice). For the analysis multiple (at least 5) similarly sized metastases were analyzed. One dot represents one mouse. 2-way ANOVA. Data are mean ± s.d. unless indicated otherwise.

**Fig 3:**
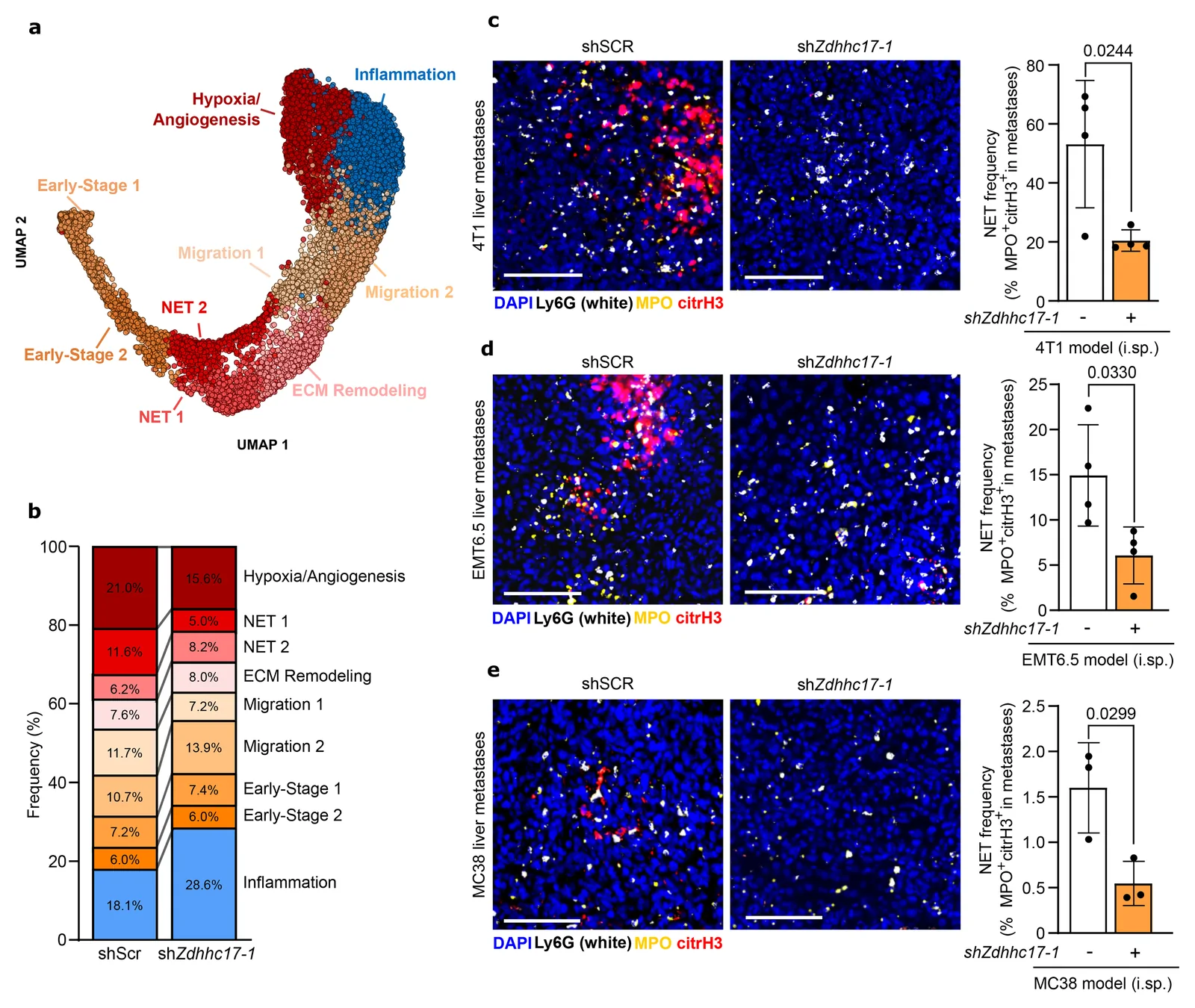
*Zdhhc17* silencing in cancer cells results in less NETosis within the metastases a. Neutrophil-specific UMAP plot, highlighting the different neutrophil subsets identified based on unsupervised sub-clustering of scRNAseq data from shSCR and sh*Zdhhc17-1* 4T1 liver metastases. b. Relative frequency distribution for the 9 neutrophil subsets identified in Fig. 3a across shSCR (left bar) and sh*Zdhhc17-1* (right bar) 4T1 liver metastases c. Left: Representative fluorescent staining for NETs in shSCR and sh*Zdhhc17-1* 4T1 liver metastases at day 14 after injection. Blue, DAPI nuclear staining; white, Ly6G; red, citrullinated histone H3 (citrH3); yellow, myeloperoxidase (MPO). Scale bar 100 µm. Right: Percent of NET release by mouse neutrophils based on double positive staining of MPO and citrH3 (n=4 mice). For the analysis, multiple (at least 5) similarly sized metastases were taken. Two-sided unpaired t-test. d. Left: Representative fluorescent staining for NETs in shSCR and sh*Zdhhc17-1* EMT6.5 liver metastases at day 14 after injection. Blue, DAPI nuclear staining; white, Ly6G; red, citrH3; yellow, MPO. Scale bar 100 µm. Right: Percent of NET release by mouse neutrophils based on double positive staining of MPO and citrH3 (n=4 mice). For the analysis, multiple (at least 5) similarly sized metastases were taken. Two-sided unpaired t-test. e. Left: Representative fluorescent staining for NETs in shSCR and sh*Zdhhc17-1* MC38 liver metastases at day 14 after injection. Blue, DAPI nuclear staining; white, Ly6G; red, citrH3; yellow, MPO. Scale bar 100 µm. Right: Percent of NET release by mouse neutrophils based on double positive staining of MPO and citrH3 (n=3 mice). For the analysis, multipole (at least 5) similarly sized metastases were taken. Twosided unpaired t-test. Data are mean ± s.d. unless indicated otherwise.

Next, we asked how neutrophils mediate the decrease in liver metastases growth upon *Zdhhc17* silencing in cancer cells. For this, we assessed the gene expression patterns of different neutrophil subsets within resected liver metastases from *Zdhhc17-*silenced and control 4T1 cancer cells using BD Rhapsody single-cell RNA-sequencing (Extended Data Fig. 3a). We identified 9 different neutrophil subsets using an unsupervised sub-clustering approach, to which we annotated 6 distinct phenotypes based on the top most differentially enriched genes in each subset relative to all other subsets, and on the overlaps observed between the top markers of each pair of subsets (Fig. 3a, Extended Data Fig. 3b-c). Subsequently, we assessed the changes in the relative frequencies of these neutrophil subsets between metastases from *Zdhhc17-*silenced and control cancer cells (Fig. 3b, Extended Data Fig. 3d). We observed that the neutrophil subsets defined by genes indicative of hypoxia and angiogenesis as well as neutrophil extracellular traps (NETs) were both reduced by ~1.35-fold, suggesting a decrease in protumour neutrophils^28^ (Fig. 3b). To assess the protumour behaviour of neutrophils, we visualized NETs in the liver metastases by performing a multiplex staining for MPO and citrullinated histone H3 (citrH3), together with Ly6G as general mouse neutrophil marker^29,30^. In line with the phenotypic characterization of the neutrophil subsets, we found less NETs in metastases from *Zdhhc17*-silenced cancer cells compared to control metastases in the 4T1, EMT6.5 and the MC38 mouse models (Fig. 3c-e, Extended Data Fig. 3e), which was not significantly altered by the presence of CD8^+^ T cells (Extended Data Fig. 3f). Thus, we concluded that DHHC17-expressing cancer cells promote NET releasing neutrophils within liver metastases independently of CD8^+^ T cells.

### Neutrophils show increased cancer cell killing upon DHHC17 loss in cancer cells

**Extended Data Fig 4:**
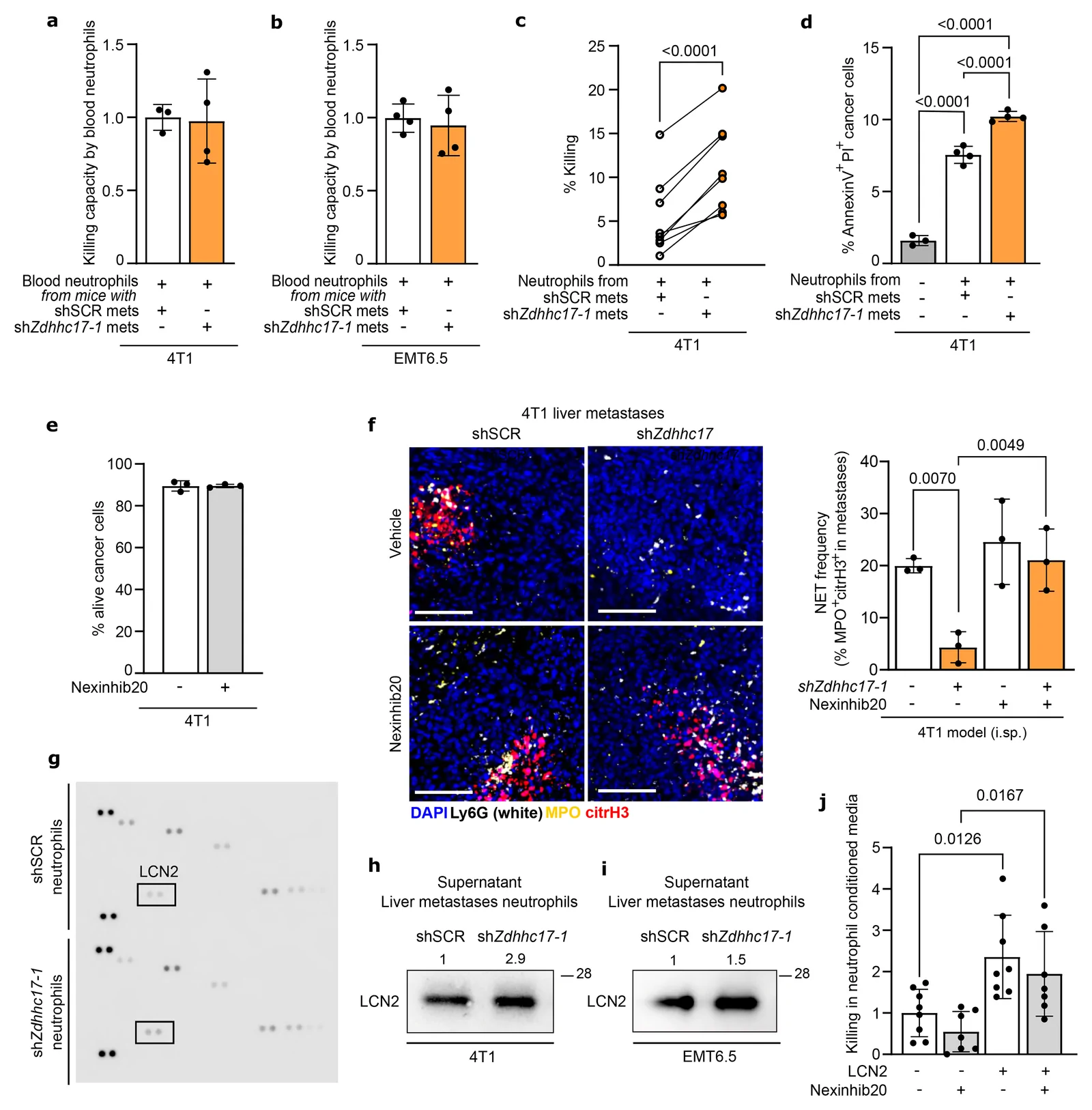
Functional changes in neutrophils from liver metastases a. Cancer cell killing capacity of blood neutrophils isolated from mice with 4T1 shSCR or *shZdhhc17-1* liver metastases after co-culture with 4T1 wild-type cells for 48h. A representative of n=3 experiments is shown. Each dot represents blood neutrophils isolated from one mouse (n=3-4 mice). Unpaired two-tailed t-test. b. Cancer cell killing capacity of blood neutrophils isolated from mice with EMT6.5 shSCR or sh*Zdhhc17-1* liver metastases after co-culture with EMT6.5 wild-type cells for 48h. A representative of n=3 experiments is shown. Each dot represents blood neutrophils isolated from one mouse (n=4 mice). Unpaired two-tailed t-test. c. Matched cancer cell killing capacity of neutrophils isolated from 4T1 shSCR or sh*Zdhhc17-1* liver metastases after coculture with 4T1 wild-type for 48h. Each dot represents the average of three or four mice analyzed at the same time (n=8). Mice from 6 of the 8 experiments are also shown in Fig. 6A. Each data point is the average of semi-biological replicates that originate from the same batch of neutrophil isolation. A paired two-tailed t-test analysis was used because the pairs are defined as average of semi-biological replicates per batch of neutrophil isolation covering control and sh*Zdhhc17* metastasis from mice of the same cohort. d. Percent AnnexinV^+^ PI^+^ cancer cells upon monoculture or co-culture with neutrophils from 4T1 shSCR and *shZdhhc17-1* liver metastases for 48h. A representative of n=4 experiments is shown. Each dot represents neutrophils isolated from one mouse (n=4 mice). One-way ANOVA. e. Percent alive 4T1 cancer cells after 48h treatment with or without Nexinhib20 (0.5 µM) (n=3 biological replicates) f. Left: Representative fluorescent staining for NETs in 4T1 shSCR and sh*Zdhhc17-1* liver metastases and treated with Nexinhib20 or vehicle at day 14 after injection. Blue, DAPI nuclear staining; green, Ly6G; red, citrH3; yellow, MPO. Scale bar 100 µm. Right: Percent NET release by mouse neutrophils based on double positive staining of MPO and citrH3 (n=3 mice). For the analysis multiple (at least 5) similarly sized metastases were analyzed. 2-way ANOVA. g. Cytokine array of conditioned media of neutrophils from 4T1 shSCR or *shZdhhc17* liver metastases. LCN2 is marked with rectangle. h. Protein levels of LCN2 in conditioned media of neutrophils from 4T1 shSCR and *shZdhhc17-1* liver metastases. Numbers above the bands indicate the relative band intensity. A representative blot of n=3 experiments is shown. i. Protein levels of LCN2 in conditioned media of neutrophils from EMT6.5 shSCR and *shZdhhc17-1* liver metastases. Numbers above the bands indicate the relative band intensity. A representative blot of n=3 experiments is shown. j. Cancer cell killing of 4T1 cells after 48h culture in conditioned media from neutrophils isolated from 4T1 shSCR liver metastases, treated with Nexinhib20 (0.5 µM) or vehicle, and with or without LCN2 (0.5µg/ml). Each dot represents conditioned media from neutrophils isolated from one mouse (n=7 mice for Nexinhib20 -/+ LCN2, n=8 for control and +LCN2), One-way ANOVA. Data are mean ± s.d. unless indicated otherwise.

**Fig 4:**
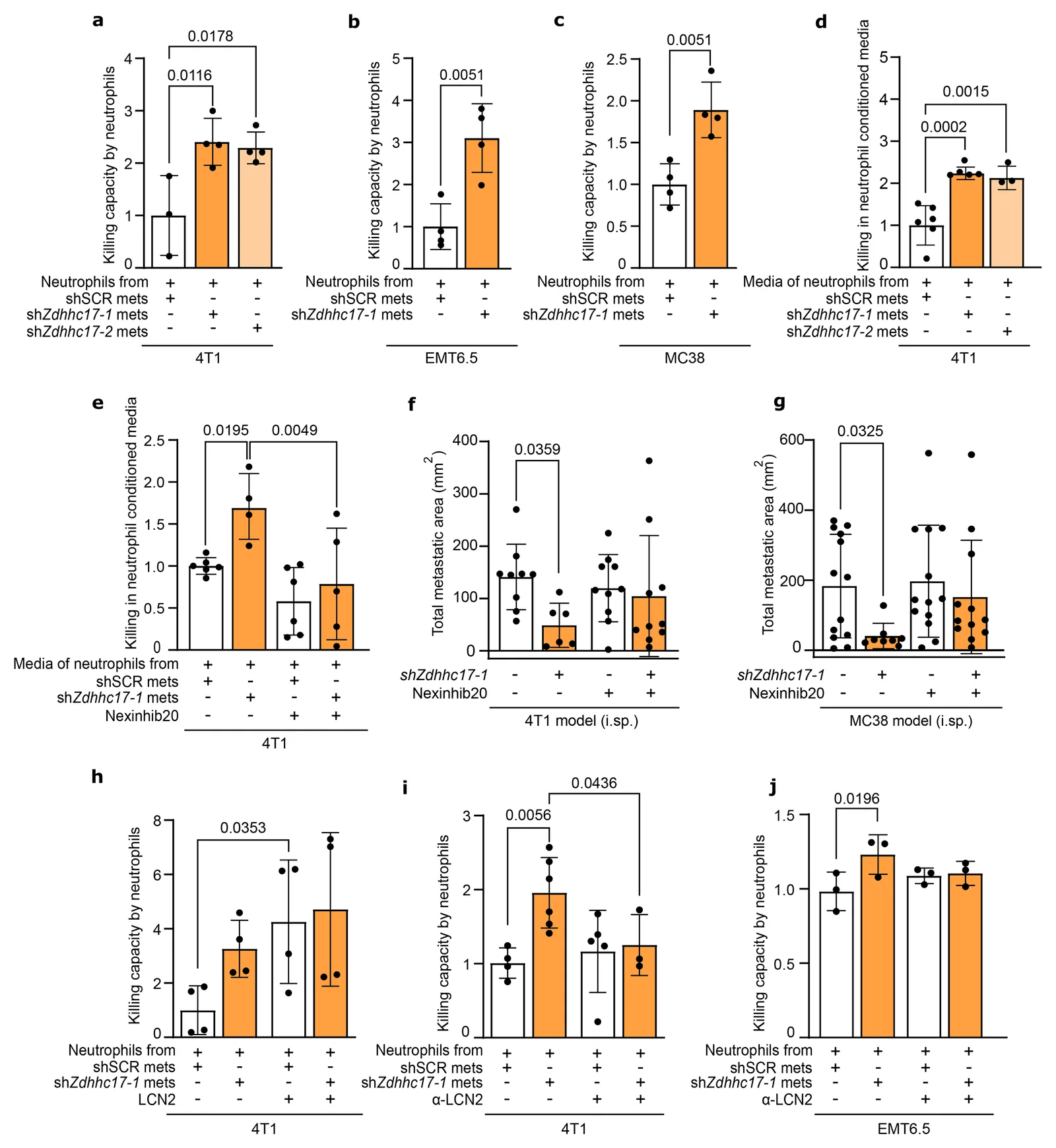
Neutrophil degranulation contributes to the killing of *Zdhhc17*-silenced cancer cells a. Cancer cell killing capacity of neutrophils isolated from 4T1 shSCR, *shZdhhc17-1* and sh*Zdhhc17-2* liver metastases after co-culture with 4T1 wild-type cells for 48h. A representative of n=3 experiments is shown. Each dot represents neutrophils isolated from one mouse (n=3 mice for shSCR, n=4 for sh*Zdhhc17*-1 and -2). One-way ANOVA with Dunnett’s multiple comparison test. b. Cancer cell killing capacity of neutrophils isolated from EMT6.5 shSCR and sh*Zdhhc17-1* liver metastases after co-culture with EMT6.5 wild-type cells for 48h. A representative of n=3 experiments is shown. Each dot represents neutrophils isolated from one mouse (n=4 mice). Two-sided unpaired t-test. c. Cancer cell killing capacity of neutrophils isolated from MC38 shSCR and sh*Zdhhc17-1* liver metastases after co-culture with MC38 wild-type cells for 48h. A representative of n=3 experiments is shown. Each dot represents neutrophils isolated from one mouse (n=4 mice). Two-sided unpaired t-test. d. Cancer cell killing of 4T1 cells after 48h of culture in conditioned media from neutrophils isolated from 4T1 shSCR and sh*Zdhhc17-1* liver metastases. Each dot represents conditioned media from neutrophils isolated from one mouse (n=3 mice for *shZdhhc17*-2, n=5 mice for *shZdhhc17-1* and n=6 mice for shSCR). One-way ANOVA with Dunnett’s multiple comparison test. e. Cancer cell killing of 4T1 cells after 48h culture in conditioned media from neutrophils isolated from 4T1 shSCR and sh*Zdhhc17-1* liver metastases and treated with Nexinhib20 (0.5 µM) or vehicle. Each dot represents conditioned media from neutrophils isolated from one mouse (n=4 mice for sh*Zdhhc17-1*, n=5 sh*Zdhhc17-1* +Nexinhib20, n=6 for shSCR). One-way ANOVA with Dunnett’s multiple comparison test. f. Total metastatic area in livers of mice *intrasplenically* injected with shSCR or sh*Zdhhc17-1* 4T1 cells and treated with Nexinhib20 (30mg/kg) (n=9 shSCR, n=6 sh*Zdhhc17*-1) or vehicle (n=10 each) starting 24h post cancer cell injection. 2-way ANOVA. Representative H&E images are shown in Extended Data Fig. 9n. g. Total metastatic area in livers of mice *intrasplenically* injected with shSCR or sh*Zdhhc17-1* MC38 cells and treated with Nexinhib20 (30mg/kg) (n=8 shSCR, n=10 sh*Zdhhc17*) or vehicle (n=13 shSCR, n=9 sh*Zdhhc17*). 2-way ANOVA. Representative H&E images are shown in Extended Data Fig. 9o. h. Cancer cell killing capacity of neutrophils isolated from 4T1 shSCR and *shZdhhc17-1* liver metastases after 48h co-culture with 4T1 wild-type cells and in presence of LCN2 (0.5µg/ml). Each dot represents neutrophils isolated from one mouse (n=4 mice). 2-way ANOVA. i. Cancer cell killing capacity of neutrophils isolated from 4T1 shSCR and *shZdhhc17-1* liver metastases after co-culture with 4T1 wild-type cells and in presence of a LCN2-capturing antibody (1:1000) or isotype control antibody (1:1000) for 48h. Each dot represents neutrophils isolated from one mouse (n=3 mice for sh*Zdhhc17* + α-LCN2, n=4 for shSCR control, n=5 for shSCR + α-LCN2 and n=6 for sh*Zdhhc17* control). 2-way ANOVA. j. Cancer cell killing capacity of neutrophils isolated from EMT6.5 shSCR and *shZdhhc17-1* liver metastases in co-culture with EMT6.5 wild-type cells and in presence of a LCN2-capturing antibody (1:1000) or isotype control antibody (1:1000) for 48h. Each dot represents neutrophils isolated from one mouse (n=3 mice). 2-way ANOVA. Data are mean ± s.d. unless indicated otherwise.

Next, we asked whether an increased killing of cancer cells by neutrophils is responsible for the observed decrease in liver metastasis growth upon loss of DHHC17 in cancer cells. We isolated neutrophils from metastases of *Zdhhc17-*silenced and control 4T1, EMT6.5 and MC38 cancer cells and peripheral neutrophils from the blood of the same mice. Subsequently, we co-cultured the metastases and blood-isolated neutrophils with parental 4T1, EMT6.5 and MC38 cancer cells, and assessed the percentage of live and dying cancer cells after co-culture using a viability dye as well as Annexin V and PI double positivity as apoptosis markers. Based on the percentage of live cancer cells in the co-cultures versus monoculture (*i*.*e*. no neutrophils added), we calculated the killing capacity of the neutrophils. We observed no difference in cancer cell killing capacity by blood-isolated neutrophils from mice with control or *Zdhhc17*-silenced metastases (Extended Data Fig. 4a-b). However, neutrophils isolated from *Zdhhc17*-silenced liver metastases showed an increased cancer cell killing capacity compared to neutrophils isolated from control metastases (Fig. 4a-c, Extended Data Fig. 4c), which was accompanied by an increase in apoptosis in the cancer cells (Extended Data Fig. 4d). Thus, we concluded that neutrophils from metastases of *Zdhhc17-*silenced cancer cells more potently kill cancer cells.

Next, we investigated which molecular process is linked to the increased cancer cell-killing capacity of neutrophils in metastases from *Zdhhc17-*silenced cancer cells. One potential mechanism is degranulation, which is the release of cytokines and proteins able to kill cancer cells^31,32^. Therefore, we investigated whether media conditioned by neutrophils isolated from *Zdhhc17-*silenced metastases is sufficient to increase cancer cell killing. Indeed, we observed that the conditioned media from neutrophils isolated from *Zdhhc17-*silenced metastases resulted in a higher cancer cell killing compared to conditioned media from neutrophils isolated from control metastases (Fig. 4d). Thus, we next inhibited degranulation in neutrophils isolated from metastases by treating them *ex vivo* with the neutrophil degranulation inhibitor Nexinhib20^33^ (0.5 µM) or vehicle. Subsequently we transferred the neutrophil-conditioned media to 4T1 cancer cells. We also treated cancer cells directly with Nexinhib20 and excluded a direct impact of the drug on cancer cells (Extended Data Fig. 4e). We found that the increased cancer cell killing observed in conditioned media from neutrophils of *Zdhhc17*-silenced metastases was dampened when the neutrophils were treated with Nexinhib20 (Fig. 4e). This suggests that degranulation is important for the increased cancer cell killing capacity of neutrophils from *Zdhhc17-*silenced metastases. Subsequently, we assessed the effect of degranulation inhibition on liver metastasis growth. We injected mice *intrasplenically* with *Zdhhc17*-silenced and control 4T1 and MC38 cancer cells and treated mice with Nexinhib20 (30 mg/kg, 3 times per week) or vehicle ^27^. We found that treating mice with Nexinhib20 abrogated the decrease in metastasis growth previously observed upon *Zdhhc17*-silencing in 4T1 and MC38 cancer cells (Fig. 4f-g). In line, inhibition of degranulation rescued NET release in liver metastases from *Zdhhc17*-silenced cancer cells, potentially due to the increased intracellular levels of azurophilic granules that can translocate to the nucleus^34^ (Extended Data Fig. 4f). Thus, we concluded that neutrophil degranulation is required for the decrease in metastasis growth upon *Zdhhc17* silencing in cancer cells.

We next explored which cytokines released by neutrophils during degranulation are responsible for the enhanced cancer cell killing in *Zdhhc17*-silenced metastases. We performed a cytokine array against >100 soluble proteins in the conditioned media from neutrophils isolated from *Zdhhc17*-silenced liver metastases versus control (Extended Data Fig. 4g). We found that lipocalin-2 (LCN2; also known as neutrophil gelatinase-associated lipocalin, NGAL) was increased in the media conditioned by neutrophils isolated from *Zdhhc17*-silenced liver metastases, which we confirmed by western blot (Extended Data Fig. 4h-i). In addition, we found that LCN2 supplementation increased the cancer cell killing capacity of neutrophils from control metastases (Fig. 4h) and increased the fraction of killed cancer cells in media from neutrophils treated with Nexinhib20 (Extended Data Fig. 4j). Conversely, scavenging LCN2 with an antibody decreased the cancer cell killing capacity of neutrophils from *Zdhhc17*-silenced metastases (Fig. 4i-j). Taken together, we concluded that neutrophils from *Zdhhc17*-silenced metastases release more LCN2 during degranulation, which contributes to their increased capacity to kill cancer cells.

### DHHC17 palmitoylates laminin-511 in cancer cells

**Extended Data Fig. 5:**
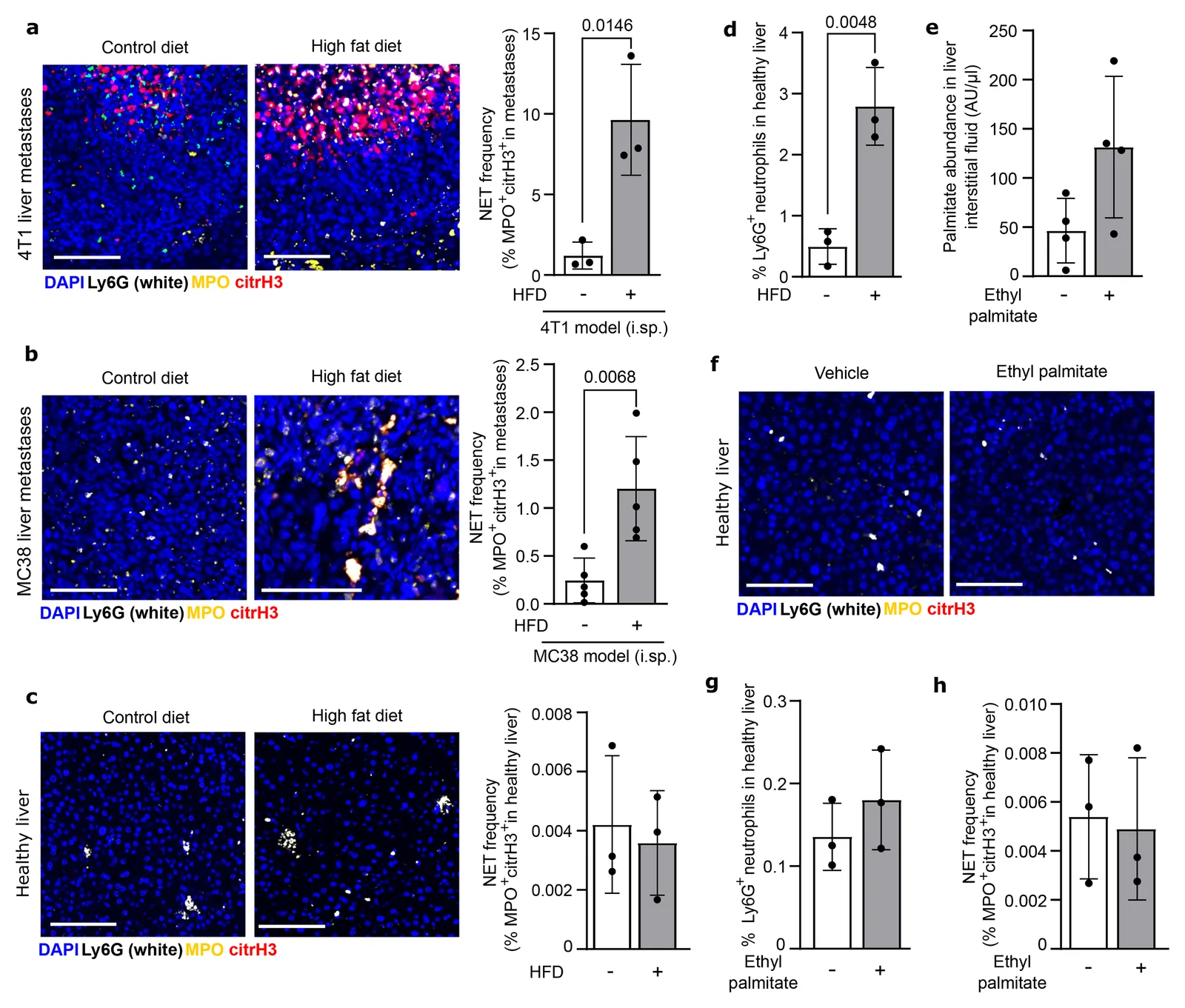
High fat diet and palmitate increase NETs in metastatic but not healthy livers a. Left: Representative fluorescent staining for NETs in 4T1 liver metastases in mice fed with control diet or HFD. Blue, DAPI nuclear staining; green, Ly6G; red, citrH3; yellow, MPO. Scale bar 100 µm. Right: Percent NET release by mouse neutrophils in 4T1 liver metastases fed with CD or HFD based on double positive staining of MPO and citrH3 (n=3 mice). For the analysis, multiple (at least 5) similar sized metastases were analyzed. Unpaired two-tailed t-test. b. Left: Representative fluorescent staining for NETs in MC38 liver metastases in mice fed with control diet or HFD. Blue, DAPI nuclear staining; green, Ly6G; red, citrH3; yellow, MPO. Scale bar 100 µm. Right: Percent NET release by mouse neutrophils in MC38 liver metastases fed with CD or HFD based on double positive staining of MPO and citrH3 (n=5 mice). For the analysis only similar sized metastases were analyzed and at least 5 metastases per mouse were analyzed. Unpaired two-tailed t-test. c. Left: Representative fluorescent staining for NETs in healthy liver of mice fed with control diet or HFD. Blue, DAPI nuclear staining; green, Ly6G; red, citrH3; yellow, MPO. Scale bar 100 µm. Right: Percent NET release by mouse neutrophils in healthy livers of mice fed with CD or HFD based on double positive staining of MPO and citrH3 (n=3 mice). Multiple (at least 5) similar sized regions in the liver were analyzed. d. Percent Ly6G^+^ neutrophils in healthy livers of mice fed with control diet and HFD (n=3 mice). Multiple similar sized regions in the liver were analyzed. e. Palmitate abundance in the interstitial fluid of healthy livers of mice *intrahepatically* injected with ethyl palmitate or vehicle for 14 days (n=4 mice). f. Representative fluorescent staining for NETs in healthy liver of mice *intrahepatically* injected with ethyl palmitate or vehicle for 14 days. Blue, DAPI nuclear staining; green, Ly6G; red, citrH3; yellow, MPO. Scale bar 100 µm. g. Percent Ly6G^+^ neutrophils in healthy livers of mice *intrahepatically* injected with ethyl palmitate or vehicle for 14 days. Multiple (at least 5) similar sized regions in the liver were analyzed (n=3 mice). h. Percent NET release by mouse neutrophils in healthy livers of mice *intrahepatically* injected with ethyl palmitate or vehicle during 14 days. Multiple (at least 5) similar sized regions in the liver were analyzed (n=3 mice). Data are mean ± s.d. unless indicated otherwise.

**Extended Data Fig. 6:**
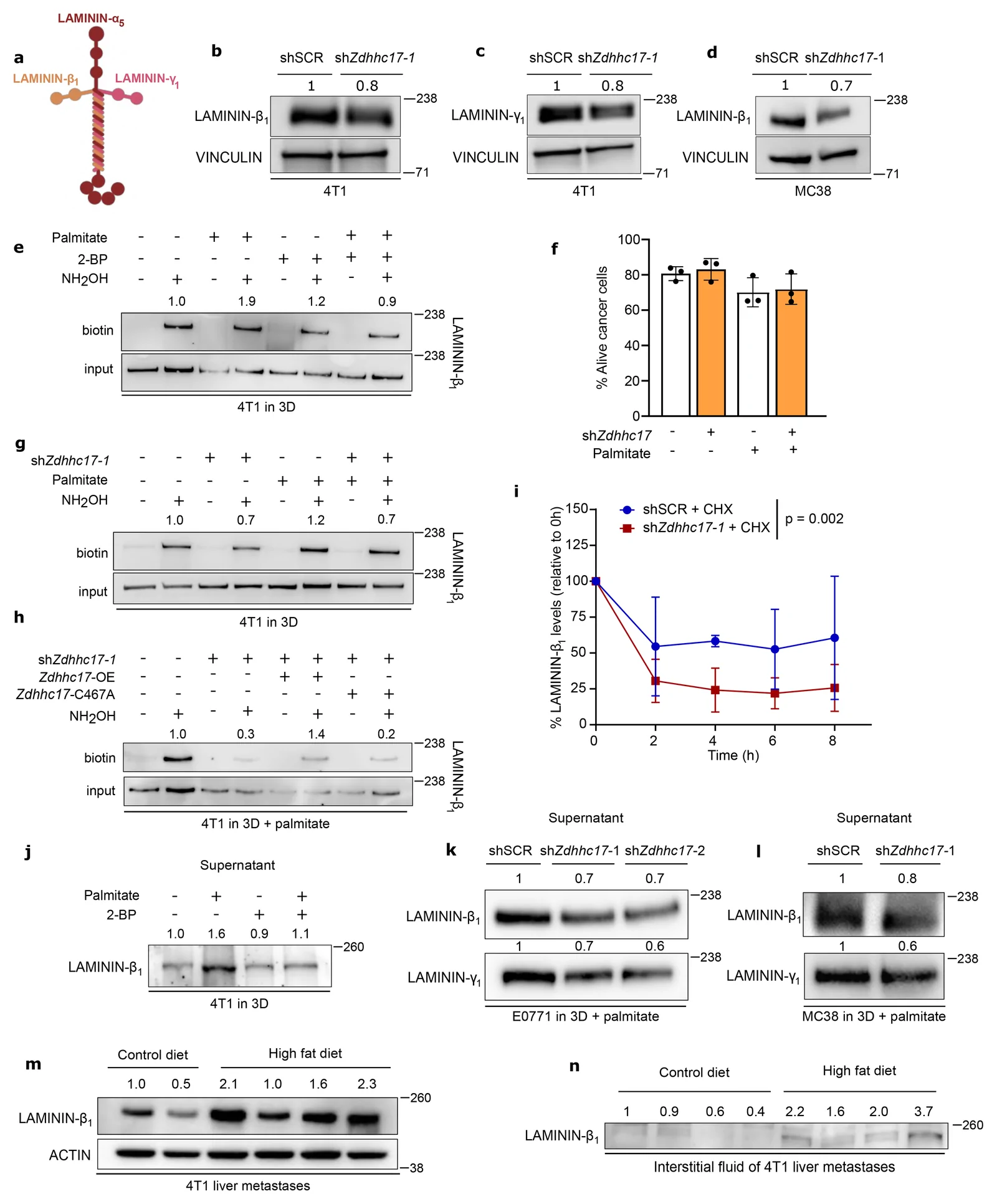
*Zdhhc17* silencing in cancer cells reduces laminin-511 levels, while palmitate promotes LAMININ-β_1_ secretion in vitro and in vivo a. Graphical illustration of laminin-511 b. Protein levels of laminin-β_1_ and VINCULIN in the lysate of 4T1 shSCR and sh*Zdhhc17-1* spheroids. Numbers above the bands indicate the relative band intensity after normalization to VINCULIN. A representative blot of n=3 experiments is shown. c. Protein levels of laminin-γ_1_ and VINCULIN in the lysate of 4T1 shSCR and sh*Zdhhc17-1* spheroids. Numbers above the bands indicate the relative band intensity after normalization to VINCULIN. A representative blot of n=3 experiments is shown. d. Protein levels of laminin-β1 and VINCULIN in the lysate of MC38 shSCR and sh*Zdhhc17-1* spheroids. Numbers above the bands indicate the relative band intensity after normalization to VINCULIN. A representative blot of n=3 experiments is shown. e. Palmitoylation of laminin-β_1_ measured using an ABE assay in 4T1 spheroids, in the presence of palmitate (75 μM), with or without 2-BP (75 µM) and with or without NH_2_OH. Numbers above the bands indicate the relative band intensity after normalization to input. A representative of n=3 experiments is shown. f. Percent alive cancer cells in control and sh*Zdhhc17*-1 4T1 spheroids supplemented with palmitate or vehicle for 5 days. Every dot represents average of technical replicates of each experiment (n=2-3). g. Palmitoylation of laminin-β_1_ measured using an ABE assay in 4T1 shSCR and *shZdhhc17-1* spheroids, in the presence/absence of palmitate and with/without NH_2_OH. Numbers above the bands indicate the relative band intensity after normalization to input. A representative blot of n=3 experiments is shown. h. Palmitoylation of laminin-β_1_ measured using an ABE assay in 4T1 shSCR and sh*Zdhhc17-1* spheroids, with overexpression of wild-type or catalytic mutant (C467A) *Zdhhc17*, in the presence of palmitate and with/without NH_2_OH. A representative blot of n=3 experiments is shown. i. Levels of laminin-β_1_ (% relative to time 0) upon cycloheximide (CHX) treatment in 4T1 shSCR versus *shZdhhc17-1* spheroids, quantified over n=4 independent biological replicates. Statistical analysis based on one-sided F-Test on 3rd degree polynomials, with F-Test p-value shown. The intercepts of the polynomials (values at time 0) were manually set to 100% by virtue of the experimental design. j. Protein levels of laminin-β_1_ in the supernatant of 4T1 spheroids in the presence of palmitate, 2-BP or both. Numbers above the bands indicate the relative band intensity. A representative blot of n=3 experiments is shown. k. Protein levels of laminin-β_1_ and laminin-γ_1_ in the supernatant of E0771 shSCR, sh*Zdhhc17-1* and sh*Zdhhc17-2* spheroids in the presence of palmitate (200 μM). Numbers above the bands indicate the relative band intensity. A representative blot of n=3 experiments is shown. l. Protein levels of laminin-β_1_ and laminin-γ_1_ in the supernatant of MC38 shSCR and sh*Zdhhc17-1* spheroids in the presence of palmitate (200 μM). Numbers above the bands indicate the relative band intensity. A representative blot of n=3 experiments is shown. m. Protein levels of laminin-β1 in 4T1 liver metastases from mice fed with a control diet or HFD for 16 weeks. Every sample is from a mouse (n=2 and n=4) and numbers above the bands indicate relative band intensity, normalized by actin. n. Protein levels of laminin-β_1_ in the interstitial fluid of 4T1 liver metastases in mice fed with a control diet or HFD for 16 weeks. Every sample is from a mouse (n=4 mice) and numbers above the bands indicate the relative band intensity. Data are mean ± s.d. unless indicated otherwise.

**Fig 5:**
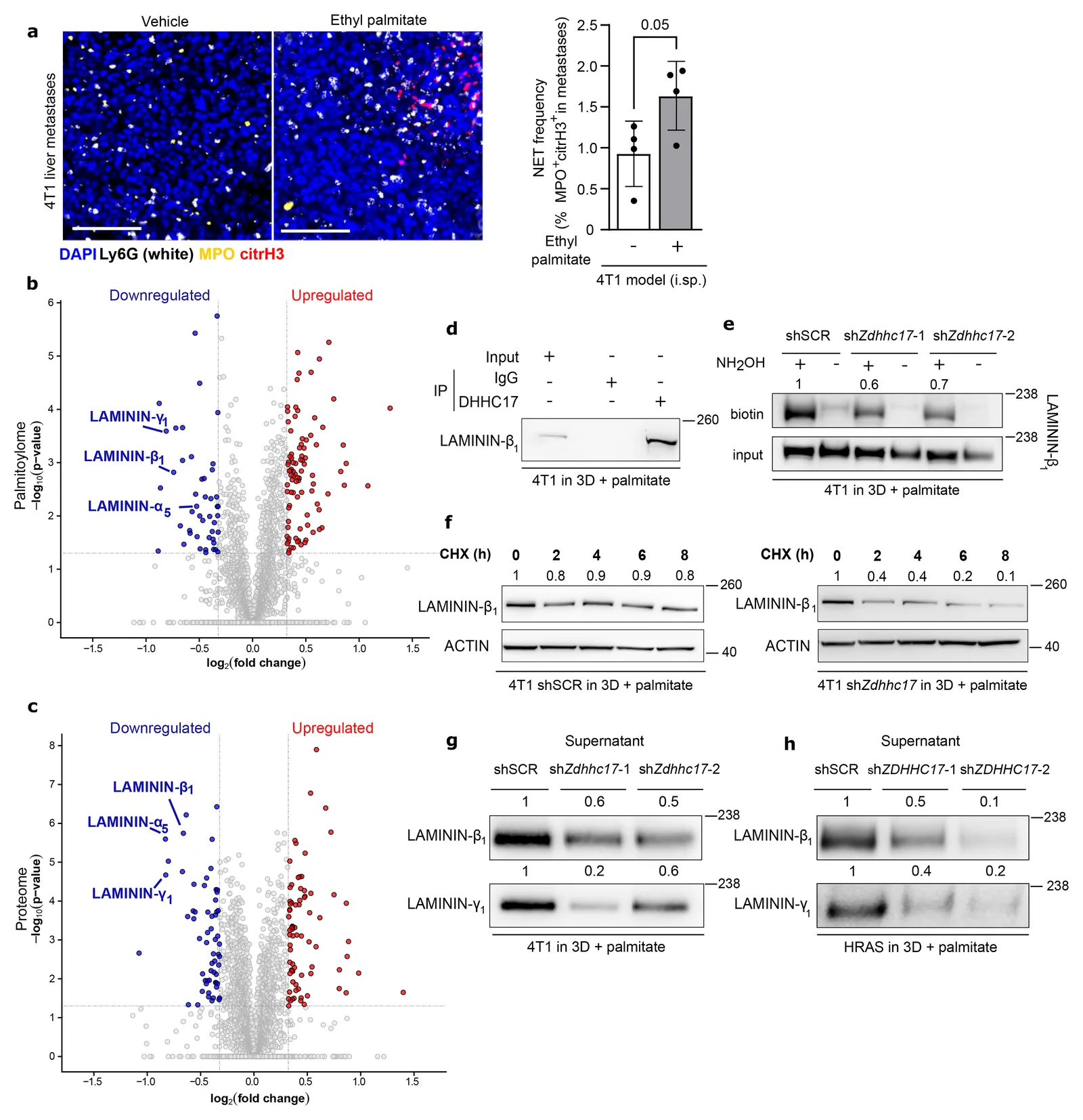
Laminin-511 is palmitoylated by DHHC17 in cancer cells a. Left: Representative fluorescent staining for NETs in 4T1 liver metastases in mice injected with ethyl palmitate or control. Blue, DAPI nuclear staining; white, Ly6G; red, citrH3; yellow, MPO. Scale bar 100 µm. Right: Percent NET release by mouse neutrophils in the metastases based on double positive staining of MPO and citrH3 (n=4 mice). For the analysis, multiple (at least 5) similarly sized metastases were taken. Two-sided unpaired t-test. b. Palmitoylome proteomics from 4T1 sh*Zdhhc17-1* versus shSCR spheroids treated with 17-ODYA (75 µM) for 5 days (n=6 biological replicates). Log_2_ fold changes (FC) and negative log_10_-transformed *P*-values are indicated in the x and y axes respectively. The horizontal dashed line represents a *P*-value of 0.05, while the vertical ones represent absolute fold-changes of 1.5. Two-sided one-sample t-test. Laminin-α_5_, laminin-β_1_ and laminin-γ_1_ are indicated in the figure. c. Global proteome from 4T1 sh*Zdhhc17-1* versus shSCR spheroids treated with 17-ODYA (75 µM) for 5 days (n=6 biological replicates). Log_2_ fold changes (FC) and negative log_10_-transformed *P*-values are indicated in the x and y axes respectively. The horizontal dashed line represents a raw *P*-value of 0.05, while the vertical ones represent absolute fold-changes of 1.5. Two-sided one-sample t-test. Laminin-α_5_, laminin-β_1_ and laminin-γ_1_ are indicated in the figure. d. Co-immunoprecipitation of DHHC17 and laminin-β_1_ in 4T1 spheroids supplemented with palmitate (75 µM). IP, immunoprecipitation. Antibodies for IP and Western blot analyses are indicated. A representative of n=3 experiments is shown. e. Palmitoylation of laminin-β_1_ measured using an ABE assay in 4T1 shSCR, sh*Zdhhc17-1* and sh*Zdhhc17-2* spheroids, in the presence of palmitate (75 μM) and with/without NH_2_OH (hydroxylamine) to cleave the palmitoylated sites. Numbers above the bands indicate the relative band intensity after normalization to input. A representative of n 3 experiments is shown. f. Protein levels of laminin-β_1_ from total lysates of 4T1 shSCR (left) and sh*Zdhhc17-1* (right) spheroids, treated with cycloheximide (CHX) for various timepoints reaching from 2h until 8h. Numbers above the bands indicate the relative band intensity after normalization to β-ACTIN. A representative of n=3 experiments is shown. g. Protein levels of laminin-β_1_ and laminin-γ_1_ in the supernatant of 4T1 shSCR, sh*Zdhhc17-1* and sh*Zdhhc17-2* spheroids, in the presence of palmitate (75 μM). Numbers above the bands indicate the relative band intensity. A representative of n=3 experiments is shown. h. Protein levels of laminin-β_1_ and laminin-γ_1_ in the supernatant of MCF10A H-RAS^V12^ shSCR, sh*Zdhhc17-1* and sh*Zdhhc17-2* spheroids in the presence of palmitate (75 μM). Numbers above the bands indicate the relative band intensity. A representative of n=3 experiments is shown. Data are mean ± s.d. unless indicated otherwise.

Next, we investigated how DHHC17 expression in cancer cells modulates the observed neutrophil behaviour. We focused on the catalytic function of DHHC17 in cancer cells because we observed that both a systemic increase in lipids by HFD feeding and a local increase in palmitate by ultrasound-guided liver injection increased NETs in metastatic but not control livers (Fig. 5a, Extended Data Fig. 5a-h). Specifically, we assessed the DHHC17-mediated palmitoylome changes in 4T1 cancer cells with click-chemistry based proteomics^35^. We detected 2828 proteins of which 124 were differentially palmitoylated in *Zdhhc17*-silenced compared to control 4T1 cells (Fig. 5b). Proteins that decreased in both palmitoylation and protein expression included laminin-α_5_ (gene name *Lama5)*, laminin-β_1_ (gene name *Lamb1*) and laminin-γ_1_ (gene name *Lamc1*) (Fig. 5b-c, Extended Data Fig.6a-d), which together form the laminin-511 complex. Interestingly, laminins have been proposed to trigger increased parasite killing by neutrophils through NETs^36^. Thus, we asked whether cancer cells hijack this natural function of neutrophils by secreting laminin-511 in a DHHC17-dependent manner. In line, we observed that DHHC17 interacts with laminin-β_1_ (Fig. 5d), and that laminin-β_1_ palmitoylation depends on *Zdhhc17* expression (Fig. 5e). We further confirmed that laminin-β_1_ palmitoylation was affected by DHHC17 activity, which we modulated by treatment with the palmitoylation inhibitor 2-BP or extra palmitate (at concentrations not affecting cell viability), in the presence or absence of DHHC17, and by mutation of the DHHC17 catalytic site^37^ (Extended Data Fig. 6e-h). Moreover, we found that loss of *Zdhhc17* decreased the stability of laminin-β_1_ (Fig. 5f, Extended Data Fig. 6i). Next, we assessed whether these DHHC17- and palmitate-dependent changes in laminin-β_1_ levels influenced the amount of secreted laminin-511, required for a potential communication with neutrophils of the metastasis microenvironment. We measured the amount of secreted laminin-511 based on immunoblotting for laminin-β_1_ and -γ_1_ in the culture supernatant of mouse 4T1 and MC38 cancer cells as well as mouse E0771 and human MCF10A H-Ras^V12^ breast cancer cells. We found that palmitate supplementation enhanced the amount of laminin-β_1_, while silencing of *Zdhhc17* decreased the amount of laminin-β_1_ and -γ_1_ in the supernatants of the cancer cells (Fig. 5g-h, Extended Data Fig. 6j-l). In line, laminin-β_1_ levels increased in 4T1 liver metastases and its interstitial fluid when mice were fed with a HFD (Extended Data Fig 6m-n). Based on these data, we concluded that the protein stability and subsequently the amount of laminin-511 secreted by cancer cells depends on the availability of palmitate and the activity of DHHC17.

### Laminin-511 blocks the antitumor behaviour of neutrophils

**Extended Data Fig. 7:**
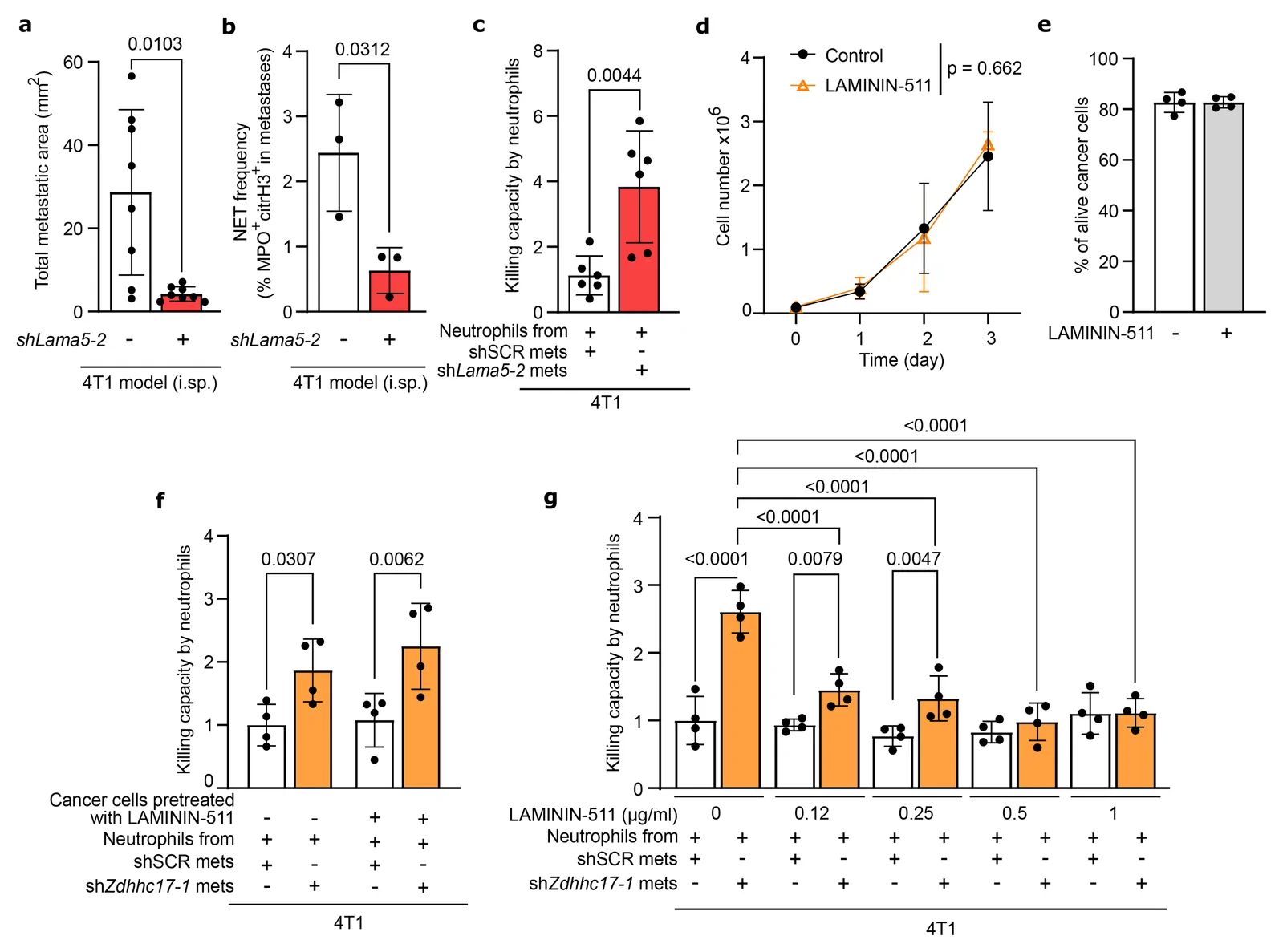
Laminin-511 does not affect cancer cells in vitro and alters neutrophil behavior in a dose-dependent manner a. Total metastatic area in livers of mice *intrasplenically* injected with shSCR or sh*Lama5-2* 4T1 cells (n=8 each). Two-tailed unpaired Mann-Whitney t test. Representative H&E images are shown in Extended Data Fig. 9e. b. Percent NET release by mouse neutrophils in the 4T1 shSCR and sh*Lama5-2* metastases based on double positive staining of MPO and citrH3 (n=3 mice). For the analysis multiple (at least 5) similarly sized metastases were analyzed. Two-tailed unpaired t-test. c. Cancer cell killing capacity of neutrophils isolated from 4T1 shSCR and sh*Lama5-2* liver metastases after co-culture with 4T1 wild-type cells for 48h. Each dot represents neutrophils isolated from one mouse. Unpaired two-tailed t-test. d. Cell counts of 4T1 cells with and without laminin-511 (0.5 µg/ml) counted 24h, 48h and 72h after start of treatment (n=6 biological replicates for day 1 and n=9 biological replicates for the other days). Statistical analysis based on one-sided F-Test on 2nd degree polynomials, with F-Test p-value shown. e. Percent alive 4T1 cancer cells after 48h treatment with or without laminin-511 (0.5 µg/ml) (n=4 biological replicates). f. Cancer cell killing capacity of neutrophils isolated from 4T1 shSCR and *shZdhhc17-1* liver metastases in co-culture with 4T1 wild-type cells for 48h. The cancer cells were pre-treated with or without laminin-511 (0.5 µg/ml) for 24h before addition of the neutrophils. Each dot represents neutrophils isolated from one mouse (n=4 mice). 2-way ANOVA. g. Cancer cell killing capacity of neutrophils isolated from 4T1 shSCR and sh*Zdhhc17*-1 liver metastases in co-culture with 4T1 wild-type cells and treated with/without laminin-511 at various doses in the co-culture for 48h. Each dot represents neutrophils isolated from one mouse (n=4 mice). 2-way ANOVA. Data are mean ± s.d. unless indicated otherwise.

**Fig 6:**
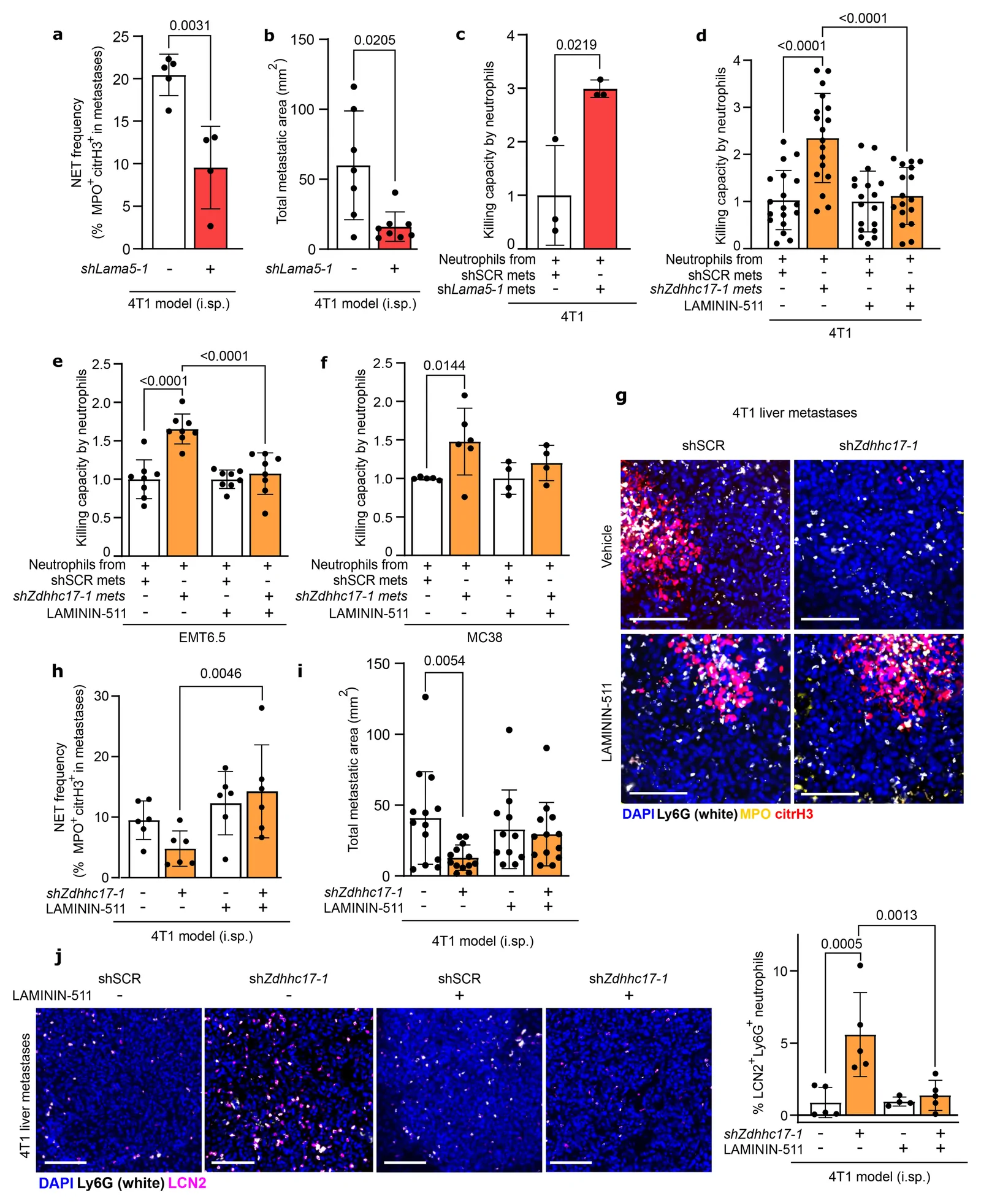
Laminin-511 impairs anti-tumor neutrophils of the liver metastasis microenvironment a. Percent NET release by mouse neutrophils in the shSCR and sh*Lama5-1* 4T1 metastases based on double positive staining of MPO and citrH3 (n=5 for shSCR, n=4 for sh*Lama5-1*). For the analysis multiple (at least 5) similarly sized metastases were analyzed. Two-sided unpaired t-test. b. Total metastatic area in livers of mice *intrasplenically* injected with shSCR or sh*Lama5-1* 4T1 cells (n=5 for control, n =8 for sh*Lama5-1*). Two-sided unpaired Mann-Whitney t-test. Representative H&E images are shown in Extended Data Fig. 9d. c. Cancer cell killing capacity of neutrophils isolated from 4T1 shSCR and sh*Lama5-1* liver metastases after co-culture with 4T1 wild-type cells for 48h. Each dot represents neutrophils isolated from one mouse (n=3 mice). Two-sided unpaired t-test. d. Cancer cell killing capacity of neutrophils isolated from 4T1 shSCR and sh*Zdhhc17*-1 liver metastases in co-culture with 4T1 wild-type cells and treated with/without laminin-511 (0.5 µg/well) in the co-culture for 48h. Each dot represents neutrophils isolated from one mouse (n=17 mice for sh*Zdhhc17* + laminin-511, n=18 mice for all others). 2-way ANOVA. The control condition (no laminin-511) is also included in Extended Data Fig 3c. e. Cancer cell killing capacity of neutrophils isolated from EMT6.5 shSCR and sh*Zdhhc17*-1 liver metastases in co-culture with EMT6.5 wild-type cells and treated with/without laminin-511 (0.5 µg/well) in the co-culture for 48h. Each dot represents neutrophils isolated from one mouse (n=8 mice). 2-way ANOVA. f. Cancer cell killing capacity of neutrophils isolated from MC38 shSCR and sh*Zdhhc17*-1 liver metastases in co-culture with MC38 wild-type cells and treated with/without laminin-511 (0.5 µg/well) in the co-culture for 48h. Each dot represents neutrophils isolated from one mouse (n=4 for the Laminin-511 condition, n=5 for shSCR control, and n=6 mice for *shZdhhc17-1* control). 2-way ANOVA. g. Representative fluorescent staining for NETs in shSCR and 4T1 sh*Zdhhc17-1* 4T1 liver metastases in mice *intrahepatically* injected with laminin-511 (10 mg/kg liver weight) or vehicle. Blue, DAPI nuclear staining; white, Ly6G; red, citrH3; yellow, MPO. Scale bar 100 µm. h. Percent NET release by mouse neutrophils in shSCR or sh*Zdhhc17-1* 4T1 metastases, *intrahepatically* injected with laminin-511 (10 mg/kg liver weight) or vehicle, based on double positive staining of MPO and citrH3 (n=6 mice). For the analysis multiple similarly sized metastases were analyzed. 2-way ANOVA. i. Total metastatic area in livers of mice *intrasplenically* injected with shSCR or sh*Zdhhc17-1* 4T1 cells and daily *intrahepatically* injected with laminin-511 (10 mg/kg liver weight) (shSCR n=11, sh*Zdhhc17* n=13) or vehicle (both n=13). 2-way ANOVA. Representative H&E images are shown in Extended Data Fig. 9p. j. Left: representative fluorescent staining for LCN2 and Ly6G in shSCR and *shZdhhc17-1* liver metastases in mice *intrahepatically* injected with laminin-511 (10 mg/kg liver weight) or vehicle. Blue, DAPI nuclear staining; white, Ly6G; cyan, LCN2. Scale bar 100 µm. Right: percent of LCN2_+_ Ly6G_+_ mouse neutrophils based on double positive staining of LCN2 and Ly6G (n=4 or 5 mice). For the analysis, multiple (at least 5) similarly sized metastases were taken. 2-way ANOVA. Data are mean ± s.d. unless indicated otherwise.

Next, we asked whether secreted laminin-511 links DHHC17 activity in cancer cells to the blocked antitumor behaviour of neutrophils. In line with this possibility, we found that silencing *Lama5* in cancer cells decreased both NETs and liver metastasis growth (Fig. 6a-b, Extended Data Fig. 7a-b) as well as neutrophil-mediated cancer cell killing (Fig. 6c, Extended Data Fig. 7c). Next, we isolated neutrophils from *Zdhhc17*-silenced and control metastases and assessed cancer cell killing upon addition of laminin-511 to co-cultures. Notably, we confirmed that (pre-) treatment of cancer cells with laminin-511 had no effect on their growth, viability, or susceptibility to neutrophil killing (Extended Data Fig. 7d-f). However, and consistent with our *in vivo* data, we observed that laminin-511 abrogated the enhanced neutrophil-mediated killing of cancer cells when neutrophils were isolated from *Zdhhc17-*silenced metastases. (Fig. 6d-f). Laminin-511 induced a dose-dependent reduction in the neutrophil-mediated killing of cancer cells when neutrophils were isolated from *Zdhhc17-*silenced metastases, yet the maximal reduction reached levels comparable to those observed with neutrophils isolated from control metastases (Extended Data Fig. 7g). This suggests that neutrophils derived from control metastases already exhibit minimal antitumor activity, likely because laminin-511 concentrations within the metastasis microenvironment are sufficient to maximally suppress the neutrophil antitumor function.

Next, we injected mice with liver metastases from control and *Zdhhc17*-silenced 4T1 cells *intrahepatically* with laminin-511 (10 mg/kg liver weight^38^, daily for 14 days). We observed that supplementing laminin-511 into the livers of mice increased NETs and growth of metastases from *Zdhhc17*-silenced cancer cells (Fig. 6g-i), while laminin-511 injections had no impact on the growth of control metastases, consistent with our dose-response *in vitro* data. In line, we observed more LCN2^+^ and Ly6G^+^ double positive neutrophils in the *Zdhhc17*-silenced metastases, which was abrogated upon laminin-511 injections (Fig. 6j). Thus, we concluded that laminin-511-derived from cancer cells expressing DHHC17 inhibited the antitumor behaviour of neutrophils in liver metastases.

### Evidence for a palmitate-instructed neutrophil behaviour in liver metastases from patients

**Extended Data Fig. 8:**
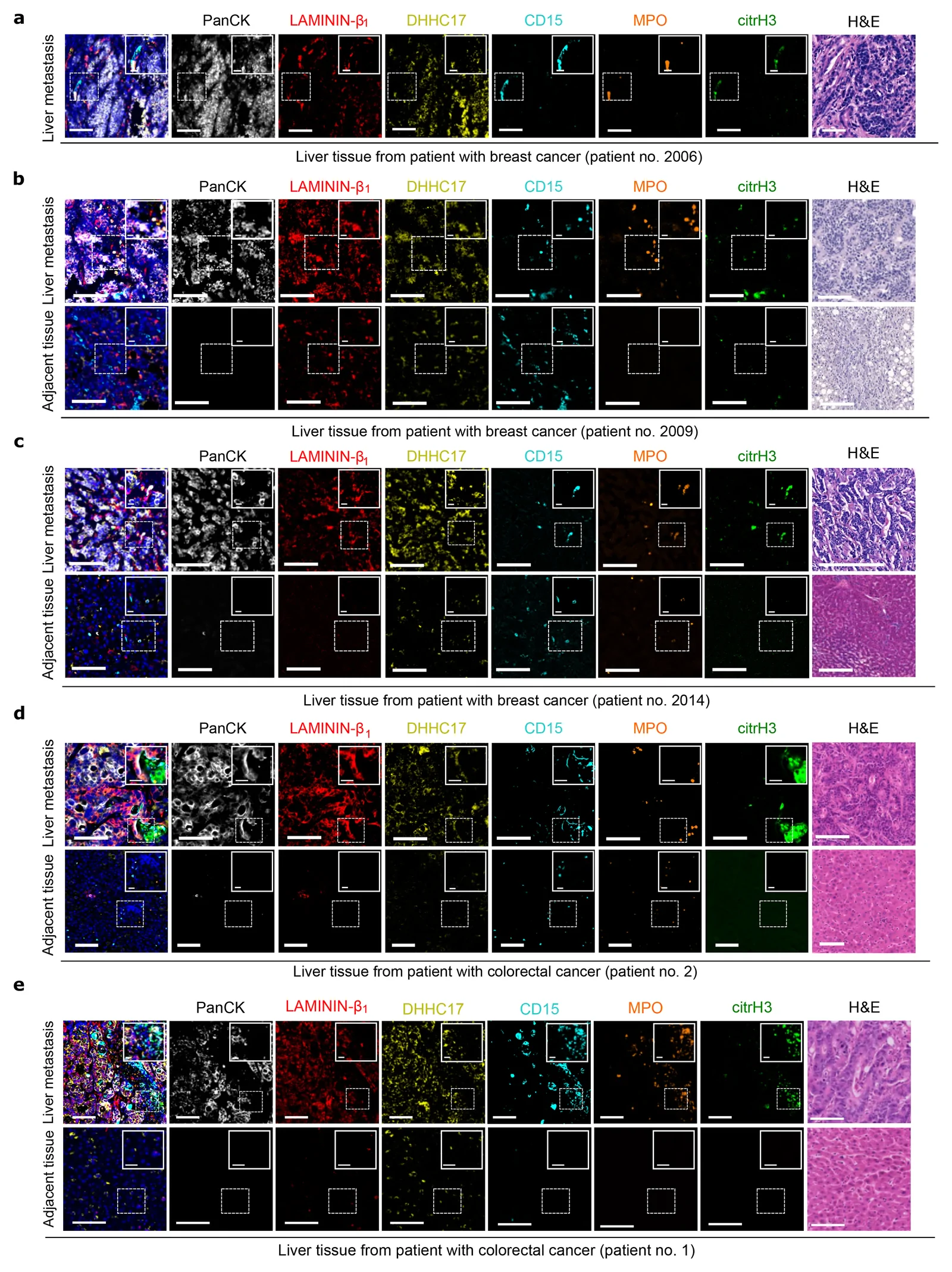
Liver tissues of patients with breast or colorectal cancer a-c. Representative images of PanCK, LAMININ-β_1_, DHHC17, CD15, MPO and citrH3 detection in metastases and adjacent non-tumor liver tissues of a patient with breast cancer from the UPTIDER program (n=3 individuals). Accompanied by matching H&E image. scale bars = 100 µm. Zoom-ins of each channel are shown. Scale bars of the zoom-in is 25µm. H&E and multiplex immunohistochemistry staining originate from consecutive cuts. d-e. Representative images of PanCK, LAMININ-β_1_, DHHC17, CD15, MPO and citrH3 detection in metastases and adjacent non-tumor liver tissues of a patient with colorectal cancer (n=2 individuals). Accompanied by matching H&E image. Scale bar 100µm. Zoom-ins of each channel are shown. Scale bars of the zoom-in is 25µm. H&E and multiplex immunohistochemistry staining originate from consecutive cuts.

**Fig 7:**
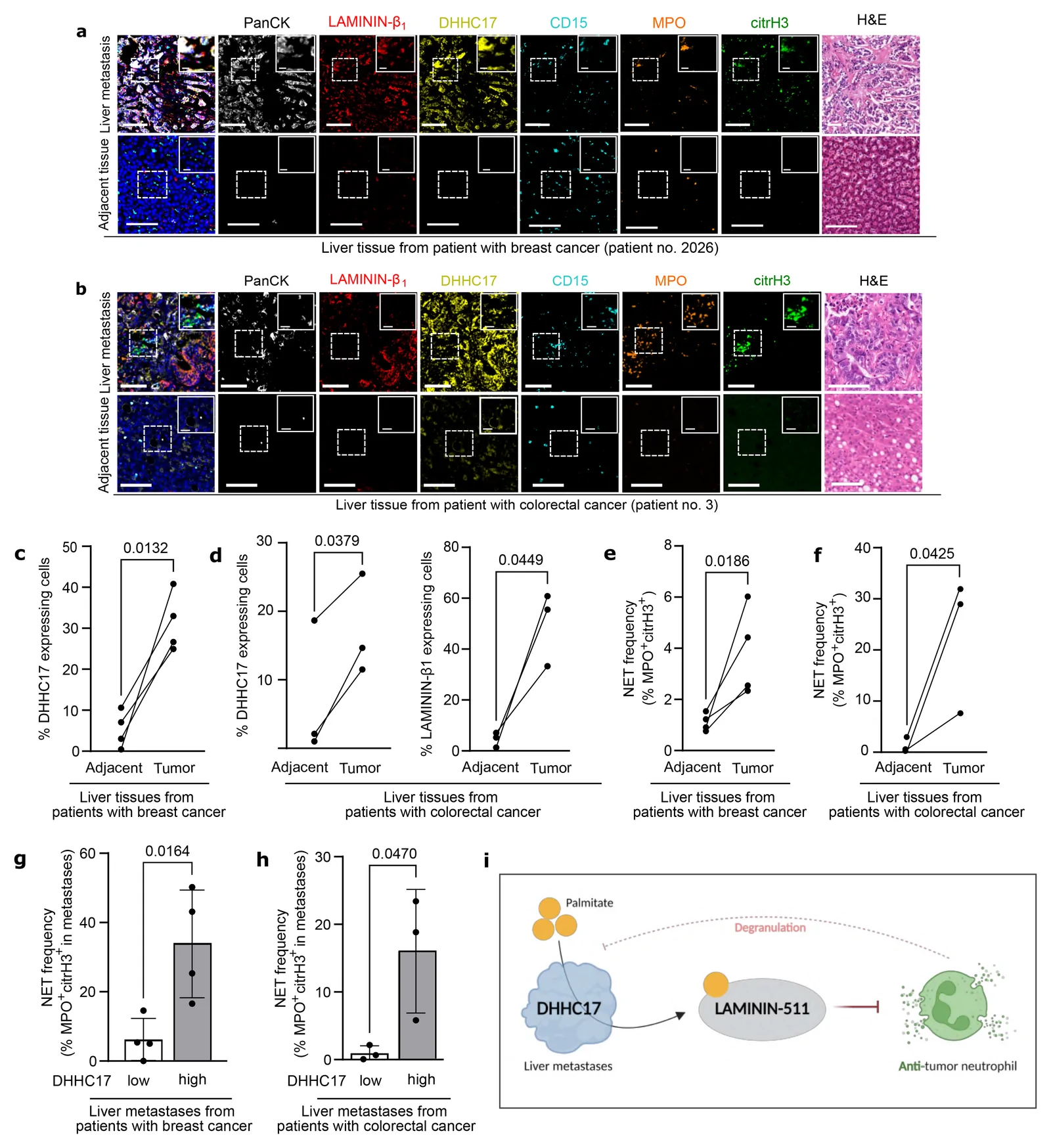
Evidence for palmitoylation instructed neutrophil behavior in liver metastases from patients with breast and colorectal cancer a. Representative images of PanCK, LAMININ-β_1_, DHHC17, CD15, MPO and citrH3 detection in metastases and adjacent non-tumor liver tissues of a human breast cancer patient from the UPTIDER program. Accompanied by matching H&E image. Scale bar 100µm. H&E and multiplex immunohistochemistry staining originate from consecutive cuts. Zoom-ins of each channel are shown. Scale bars of the zoom-in is 25µm. Representative images for additional patients are shown in Extended Data Fig. 8 (n=4 individuals). b. Representative images of PanCK, LAMININ-β_1_, DHHC17, CD15, MPO and citrH3 detection in metastases and adjacent non-tumor liver tissues of a human colorectal cancer patient. Accompanied by matching H&E image. Scale bar 100µm. H&E and multiplex immunohistochemistry staining originate from consecutive cuts. Zoom-ins of each channel are shown. Scale bars of the zoom-in is 25µm. Representative images for additional patients are shown in Extended Data Fig. 8 (n=3 individuals). c. Percent DHHC17 expressing cells in the adjacent tissue and tumor region of liver metastases of breast cancer patients from the UPTIDER program (n=4 individuals). Two-sided paired t-test. d. Left: Percent DHHC17 expressing cells in the adjacent tissue and tumor region of liver metastases of human colorectal cancer patients (n=3 individuals). Right: Percent LAMININ-β_1_ expressing cells in the adjacent tissue and tumor region of liver metastases of human colorectal cancer patients (n=3 individuals). Two-sided paired t-test. e. Percent NET release by neutrophils in the adjacent tissue and tumor region of liver metastases of breast cancer patients from the UPTIDER program (n=4 individuals), based on double positive staining of MPO and citrH3. Ratio two-sided paired t-test. f. Percent NET release by neutrophils in the adjacent tissue and tumor region of liver metastases of human colorectal cancer patients (n=3 individuals), based on double positive staining of MPO and citrH3. Ratio two-sided paired t-test. g. Percent NET release by neutrophils in human breast cancer liver metastases regions with low and high DHHC17 expression (n=4 individuals), based on double positive staining of MPO and citrH3. Two-sided unpaired t-test. h. Percent NET release by neutrophils in human colorectal cancer liver metastases regions with low and high DHHC17 expression (n=3 individuals), based on double positive staining of MPO and citrH3. Two-tailed unpaired t-test. i. Graphical representation of the identified mechanism Data are mean ± s.d. unless indicated otherwise.

Finally, we investigated the expression of key proteins in liver metastasis tissues from four patients with breast cancer collected *postmortem* (UPTIDER program, UZ Leuven, Belgium study number S64410) and from three treatment-naïve colorectal cancer patients collected by surgery (Erasmus MC Rotterdam, Netherlands, study number MEC-2018-1743) (Supplementary Table 1 and 2). We observed that the liver metastases from patients exhibited an increased expression of DHHC17, laminin-β_1_ and NETs (MPO, citrH3) as well as a trend toward elevated numbers of CD15^+^ neutrophils in the metastases compared to adjacent (non-tumour) tissue (Fig. 7a-f, Extended Data Fig. 8a-e). Next, we analysed the spatial heterogeneity of DHHC17 expression in relation to NETs. We found that liver metastasis regions with high DHHC17 expression showed also more NETs and vice versa in breast and colorectal cancer patients (Fig. 7g-h). Thus, we concluded that there is evidence for a mechanism by which cancer cells rely on DHHC17 activity to secrete laminin-511 altering neutrophil behaviour in patients with breast and colorectal cancer.

## Discussion

The liver is an organ with a particular metabolic and immune environment that enables metastasis growth from various cancer types. Here, we show that breast and colorectal cancer cells within liver metastases leverage palmitate to increase the stability of laminin-511 via DHHC17-mediated palmitoylation. In turn, cancer cell secreted laminin-511 suppresses the antitumor behaviour of neutrophils in the metastasis environment, increasing metastasis growth (Fig. 7i).

Fatty acids are highly abundant in the liver environment and specifically palmitate has been recognized for its role in promoting cancer metastasis through several cancer cell intrinsic mechanisms^9,39^. Thereby, palmitate has been shown to induce epigenetic changes in cancer cells of primary squamous cell carcinoma, which facilitates lymph node and lung metastasis^40^. Additionally, cancer cells within lung metastases oxidize palmitate resulting in the KAT2A-mediated acetylation of the NF-kB protein p65^11^, a process regulated by the palmitoylation of transmembrane 4 L six family member 1 (TM4SF1) via DHHC20^41^. Palmitate also enhances invasiveness of gastric and pancreatic cancer cells by activating Akt signaling^42^, toll-like receptor 4 (TLR4) and matrix metalloproteinase 9 (MMP-9)^43^. In contrast, some studies have indicated that palmitate may inhibit gastric cancer metastases by regulating the signal transducer and activator of transcription 3 (STAT3) signalling pathway^44^ or suppress the viability of colon cancer cells by inducing ferroptosis^45^. We add to this current knowledge by showing that cancer cells can use palmitate to palmitoylate a secreted protein. The use of palmitate in protein palmitoylation has been mainly studied in brain development and neurological disorders. Yet its role in cancer onset and progression is emerging. In the context of non-alcoholic fatty liver disease (NAFLD), metastatic colorectal cancer cells have been shown to upregulate de novo palmitate synthesis via fatty acid synthase (FASN) to drive tumour growth through a palmitoylation-dependent stabilization of epidermal growth factor receptor (EGFR)^46^. Additionally, specific palmitoyltransferases have emerged as important regulators of cancer cell proliferation, polarity, invasiveness and metastasis growth^14,41,47^. DHHC17 specifically has been identified as an oncogenic factor in NIH/3T3 cells^48^, shown to promote glioblastoma progression via interaction with mitogen-activated protein kinase 4 (MAP2K4)^49^ and to support liver tumorigenesis through palmitoylation of Akt in the context of a HFD^50^. Palmitoylation and palmitoylated proteins can also impact the tumour immune microenvironment^51^. For example, inhibition of palmitoylation of IFN-y receptor 1 (IFNGR1)^52^ or PD-L1 in cancer cells has been shown to enhance the antitumor immune response in both colorectal and breast cancer^53,54^. Furthermore, degradation of the palmitoyltransferase DHHC8 was recently found to enhance CD8^+^ T-cell cytotoxicity in melanoma^55^. While palmitoylation in metastases remains understudied, a recent study linked DHHC20 to immune modulation within metastatic niches, demonstrating that DHHC20 supports pancreatic cancer metastasis in a natural killer (NK) cell-dependent manner^56^. Complementary, we show that DHHC17 expression enables cancer cells within liver metastases to stabilize laminin-511, which impairs antitumor behaviour in neutrophils upon secreting in the metastasis microenvironment.

We found that silencing DHHC17 in cancer cells decreases liver, but not lung, metastases, while DHHC20 expression was important for lung metastasis via a cancer cell intrinsic mechanism^41^. This raises the question how this organ specific dependence on DHHC 17 arises. One contributing aspect could be that palmitate levels in the liver are much higher than in the lung^11^ and DHHC enzymes including DHHC17 undergo auto-palmitoylation, which is required for its enzymatic activity^57^. Elevated palmitate levels may therefore not only increase substrate availability but also potentiate DHHC17 activity itself. In addition, the immune compartment may also contribute to this organ specificity, as neutrophils exhibit substantial transcriptional heterogeneity and plasticity across tissues and metastatic sites^58,59^. Further investigations will be needed to disentangle how organ-specific functions of DHHC enzymes arises.

Neutrophils can adopt either a pro- or antitumor phenotype^60,59,61^. In the context of metastases, they typically exhibit a protumour behaviour that prevents T cell-mediated cancer cell elimination^62^. Specifically, neutrophils can suppress CD8^+^ T cell proliferation and cytotoxicity through nitric oxide (NO)^63^, arginase-1 (Arg1) or prostaglandin E2 (PGE2)^64^ dependent mechanisms. Additionally, by releasing NETs in the tumor microenvironment, neutrophils block the infiltration of cytotoxic T cells^65^ and also support cancer cells directly by promoting their adhesion^66^, aiding vascular remodeling^67^ and creating an inflammatory feedback loop^60^. Notably, liver metastases are particularly rich in NETs released by neutrophils in comparison to other types of metastases^30^. However, which local signals regulate the pro-tumour activities such as NETs-release remains poorly understood. Here we identified laminin-511 as a signal to alter neutrophil behaviour in the liver metastasis environment. Most importantly, we show that the absence of this signal drives neutrophils toward an antitumor behaviour, and that this is independent of CD8^+^ T cells in liver metastasis.

LCN2 is an iron-sequestering cytokine that has previously been linked to breast cancer-derived brain^68,69^ and lung metastasis^70^ as well as macrophage polarization within tumors^71^. Thereby, the role of LCN2 during tumour progression and metastasis remains highly context-dependent, varying with tumour type, disease stage and the cellular source of LCN2^72^. Most studies reporting metastasis-promoting effects of LCN2 have primarily attributed these effects to tumour cell-derived LCN2 production, whereas the contribution of LCN2 produced by tumour-associated neutrophils is relatively underexplored. Furthermore, in liver cancer LCN2 has been reported to suppress metastatic progression^73^. We add to this knowledge by showing that LCN2 contributes to cancer cell killing by neutrophils in liver metastases that lack DHHC17 expression.

The protumour role of neutrophils in the context of cancer and metastasis has triggered the idea to eliminate neutrophils from the tumour environment to increase the treatment response of patients. However, a recent clinical trial that investigated the elimination of neutrophils by targeting C-X-C motif chemokine ligand 2 (CXCL2) did not result in an increased efficacy of immunotherapy in patients with solid tumors^74^. Thus, this highlights that a more potent approach may be to switch the neutrophil behaviour from a pro- to an antitumor state. Thus, by identifying a mechanism through which cancer cells limit the antitumor behaviour of neutrophils, we contribute to the quest for therapeutic strategies that re-educate the metastasis microenvironment rather than indiscriminately eliminating all neutrophil populations.

## Materials and Methods

### Cell lines and culture conditions

4T1 (ER-/PR-/HER2-), MCF10A (ER-/PR-/HER2-), E0771 and HEK293T cells were purchased from the American Type Culture Collection (ATCC) and the MC38 cells from Kerafast. The EMT6.5 (ER-/PR-/HER2-) cells were provided by R. Anderson. MCF10A H-RAS^V12^ cells were generated by overexpressing the oncogenic driver H-RAS^V12^ in the MCF10A cells for in vitro modelling of breast cancer and metastasis. The 4T1 and EMT6.5 cells were grown in Roswell Park Memorial Institute (RPMI) 1640 medium and MC38, E0771 and HEK293T cells in Dulbecco’s Modified Eagle Medium (DMEM), both supplemented with 10% fetal bovine serum (FBS), 1% penicillin (50 units/mL) and 1% streptomycin (50 µg/mL). MCF10A H-RAS^V12^ cells were cultured in DMEM/F12 supplemented with 5% horse serum, 1% penicillin (50 units/ml), 1% streptomycin (50 μg/ml), 0.5 μg/ml hydrocortisone, 100 ng/ml cholera toxin, 10 μg/ml insulin and 20 ng/ml recombinant human EGF. All cells were cultured at 37°C with 5% CO_2_ and 95% relative humidity and were routinely tested negative for Mycoplasma infection using the MycoAlert Detection Kit (Lonza).

For the 3D cultures, the plates were covered with a soft agar-culture medium mix (1:1) as previously described^75^. Once solidified, cells were plated on top of the agar and incubated for 3-5 days. For free fatty acid palmitate supplementation, sodium palmitate (Sigma-Aldrich) was dissolved in 50% EtOH, heated at 60°C and conjugated with bovine serum albumin (BSA) at 37°C for at least one hour^11,76^. Afterwards, the FFA: BSA mix was added to the culture media to reach a final concentration of 130 µM (4T1 and MCF10A H-RAS) or 255 µM (MC38 and E0771). For control conditions, the same volume of ethanol was incubated with BSA and supplemented to the medium. Similarly, 2-BP was dissolved in 50% EtOH, heated at 60°C and conjugated with BSA at 37°C before supplementing to the cancer cells (75 µM).

For protein stability assays, 4T1 spheroids were grown with palmitate. After four days, spheroids were treated for the indicated time with/without cycloheximide (Sigma-Aldrich, 25µM) and supplemented with palmitate.

### Generation of genetically modified cell lines

*Zdhhc17* and *Lama5* knockdown cell lines were generated using the short hairpin RNA (shRNA)-expressing lentiviral TRC1 pLKO.1-shRNA vector with a puromycin selection (oligonucleotide sequences are available upon request). As negative control, a non-targeting (shSCR) (Sigma-Aldrich, SHC016) or eGFP-targeting (shSCR2) (Sigma-Aldrich, SHC005) scrambled shRNA sequence was used. For this, lentivirus was produced by transfecting HEK293T cells with psPAX2, PMD2.6 and lentiviral vectors using Lipofectamine 2000. After an overnight transduction, cells were selected with puromycin (2µg/ml for 4T1 and MC38 cells, 1µg/ml for EMT6.5 and E0771 cells, and 0.3 µg/ml for MCF10 H-RAS^V12^) for 72h. shRNA-based silencing was confirmed by RT-qPCR (Extended Data Fig. 10a-g). For overexpression of shRNA-resistant *Zdhhc17* and catalytic mutant (C467A) *Zdhhc17*, we cloned these coding sequences in the pLVX-IRES-Hyg plasmids (Takara Bio, 632182). The empty vector was used as a control. Polyclonal cells were selected with hygromycin (100 μg/ml) for 72 hours. The confirmation of the overexpression was done with RT-qPCR (Extended Data Fig. 10h)

### RNA isolation and RTqPCR

RNA was isolated from cells using the TRIzol Reagent (Thermo Fisher Scientific) and complementary DNA (cDNA) was synthesized using qScript cDNA Synthesis Kit (Quanta). Next, 20-50 ng of cDNA per reaction was amplified using SYBER Green PCR Master Mix (Life Technologies) in the 7500HT System (Applied Biosystems) as previously described^11^. The mRNA expression was normalized to an endogenous housekeeping gene (RPL19 or cyclophilin B) and relative expression was analyzed using the 2-ΔΔCt method. Gene-specific primers were designed using the NIH Primer Design Tool or obtained from PrimerBank and are listed in Supplementary Table 3.

### Western Blotting of tissue samples, lysates and supernatant

After 5 days, spheroids and supernatant were collected and centrifuged at 300 g for 5 minutes. After centrifugation, 15 ml of supernatant was filtered and concentrated for proteins <10kDa or > 100kDa using the ultra-centrifuge filter units (Amicon, Sigma-Aldrich). Cell pellets were lysed in RIPA buffer (Thermo Fisher Scientific) with protease (Roche) and phosphatase (PhosStop, Sigma) inhibitors. For tissues, metastases were resected by eye, snap frozen, cryo-grinded and lysed in RIPA buffer. Further dissociation was improved by using a tissue lyzer. Protein levels were measured using a Pierce BCA protein assay kit (Thermo Fisher Scientific). To assess the secreted laminin-511 and LCN2, we ensured to have equal cell numbers at the beginning of the experiment. Additionally, we counted the cell numbers at the end of the experiment (5 days) and normalized the loading volume based on the difference in number. For interstitial fluid analysis, equal number of proteins were loaded based on protein concentration and the extracted volume. For intracellular proteins, 20-30 µg was loaded on a NuPAGE 4-12% Bis-Tris gel (for proteins <120 kDa) or NuPAGE 3-8% Tris-Acetate gel (for proteins >120 kDa) (Thermo Fisher Scientific) and separated in MOPS SDS running buffer or Tris-Acetate buffer respectively (Thermo Fisher Scientific). After transferring to a nitrocellulose membrane (Thermo Fisher Scientific) and blocking in 5% milk in TBS with 0.05% Tween 20 for 1 hour, membranes were incubated with primary antibody overnight: LAMB1 (Proteintech, 23498-1-AP, 1:1000), LAMC1 (Cell Signaling, 92921, 1:500), ZDHHC17/HIP14 (Proteintech, 30022-1-AP, 1:500), LCN2 (Bio-Techne, AF1857, 1:500), β-ACTIN (Merck, A5441, 1:10 000 dilution) and VINCULIN (Cell Signaling, 4650S, 1:1000). Membranes were incubated with HRP-linked anti-rabbit (Cell Signaling, 7074; 1:5000 dilution), anti-goat (Dako, P0448, 1:1000) and anti-mouse IgG (Cell Signaling, 7076; 1:5000 dilution) secondary antibodies for 1 hour at room temperature and visualized using Pierce SuperSignal West Pico PLUS Chemiluminescent Substrate (Thermo Fisher Scientific). Images were acquired using an ImageQuant LAS 4000 (GE Healthcare) or a ChemiDoc MP Imaging System (BioRad). The relative band intensity was normalized to β-Actin and analyzed in ImageJ or ImageStudio.

### Acyl Biotin Exchange (ABE)-Immunoprecipitation (IP) assay

To assess palmitoylation, we used IP to isolate LAMB1 proteins and performed the ABE assay^77^. For this, spheroids of shSCR and sh*Zdhhc17* were lysed using a soft lysis buffer (50mM Tris pH 7.5, NaCl 150mM, 1mM MgCl_2_, 1% NP-40, 10% glycerol, 1mM PMSF and 1X proteinase inhibitors) containing 50 mM N-ethylmaleimide (NEM). Afterwards, 800 µg of protein was incubated with the primary antibody against LAMB1 (2 µg) overnight at 4°C in lysis buffer with 50 mM NEM. Then, Dynabeads (25 µl) (Thermo Fisher Scientific, 10008D) were added for 2 hours and the palmitoyl group was cleaved using HAM (1M in lysis buffer pH=7.2) for 1h. Next, the beads were washed and incubated with Biotin-BMCC (Fisher Scientific, 11871255, 5µM in lysis buffer pH=6.2) for 1 hour at 4°C. After washing, elution (SDS buffer with 5mM DL-Dithiothreitol (DTT) (Merck, D0632)) and heating at 80°C for 10 minutes, the samples were loaded on a 3-8% Tris-Acetate gel, transferred on a membrane, blocked with 3% BSA and incubated with Streptavidin-HRP antibody (1:5000, Abcam) overnight at 4°C. The day after, the membrane was washed and visualized using Pierce ECL Reagent (Thermo Fisher Scientific).

### Co-IP of DHHC17 and LAMININ-β_1_

To assess the interaction between DHHC17 and laminin-β1, 4T1 spheroids (supplemented with palmitate) were lysed (10mM Tris pH 7.5, NaCl 140mM, 5mM EDTA, 0.5% NP-40 and protease and phosphatase inhibitors) and incubated overnight at 4°C with Dynabeads (20µl, Thermo Fisher Scientific, 10008D) and primary antibody against DHHC17 (1µg, Proteintech, 30022-1-AP) or rabbit IgG isotype control (1µg, Invitrogen, 10500C). Then, the samples were washed and loaded on a 3-8% Tris-Acetate gel, transferred on a nitrocellulose membrane, blocked with 5% milk and incubated with a primary antibody against LAMB1 (1:1000). Next to the IP samples, an input sample of 10µg was loaded. The day after, the membrane was washed, incubated with a secondary anti-rabbit IgG conformation specific (L27A9) antibody (Cell Signaling, 5127S, 1:5000) and visualized.

### Click-chemistry based palmitoyl-proteome analysis

A detailed protocol for this technique has been written up in the book chapter “Vandekeere et al., Identification of S-palmitoylated proteins in cancer cells by metabolic labeling”^34^. In brief, 4T1 s*hZdhhc17* were grown in SILAC growth media containing heavy carbon labelled lysine (13C 15N L-Lysine hydrochloride (13C6H1415N2O2), Silantes, 211603902), 10% dialyzed FBS, 1% penicillin (50 units/ml) and 1% streptomycin (50 μg/ml), while 4T1 shSCR cells were grown in the same media but containing normal unlabeled L-lysine monohydrochloride (Merck, L8662) (referred to as 4T1 Lys8 *shZdhhc17* or 4T1 Lys0 shSCR respectively). The cells were grown in this media for over 8 passages to ensure full labeling of proteins. To assess the difference in palmitoylome between 4T1 Lys0 shSCR and Lys8 *shZdhhc17*, these cells were grown as spheroids and incubated with 17-octadecynoic acid (17-ODYA, 130µM) (SantaCruz, 34450-18-5). After 5 days, the spheroids were collected, washed with ice-cold PBS and snap-frozen in dry ice. Upon lysis (50mM HEPES pH 7.4-8, 150mM NaCl, 1% SDS, 1mM PMSF and protease inhibitors), and precipitation, 17-ODYA incorporation was validated with click-chemistry using tetramethylrhodamine (TAMRA) (Thermo Fisher Scientific, T10182).

Next, 1.5mg of the 4T1 Lys0 shSCR lysate was mixed with 1.5 mg of the 4T1 Lys8 *shZdhhc17* lysate to perform click-chemistry with Biotin-PEG3 (CAS 875770-34-6). After quenching with EDTA, precipitation, denaturation in a urea buffer, reduction with TCEP and alkylation with iodoacetamide, palmitoylated proteins were separated using magnetic streptavidin beads (Thermo Fisher Scientific, Dynabeads MyOne 65602). Before digestion, validation of each step was done by assessing samples from various steps of the protocol, of which representative images can be found in Extended Data Fig. 10i-j. Finally, the proteome and palmitoylome samples were digested using LysC (Pierce LysC Protease MS-grade, 90051). The peptides were cleaned and concentrated for mass spectrometry analysis by passing the samples through C18 (Empore SPE Disks C18, 2215) and strong cation exchange (SCX) (Empore SPE Disks Cation Exchange, 2251) chromatography columns.

### Flow cytometry analysis

For flow cytometry staining, 1×10^6^ cells per staining were incubated with anti-mouse CD16/CD32 (Fc block, BD Biosciences) for 10 minutes at 4°C and then incubated with antibodies for surface markers for 20 minutes at 4°C. Surface marker antibodies included anti-CD45 (BD Bioscience, 550994, 1:250 dilution), anti-CD8a (APC, clone 53-6.7 100711, 1:200 dilution), anti-CD11B (Biolegend, 101242, PE or BV711, 1:400), anti-Ly6G (Biolegend, 164507 and 127605, FITC or BV421, 1:200). To exclude dead cells, Viability efluor780 (Thermo Fisher Scientific, 65-0865-14, 1:2000 dilution) or Annexin V/Pi staining (Thermo Fisher Scientific, V13241) was used. The cells were washed twice and resuspended in 200 μl of FACS buffer before analysis using BD FACS Symphony with FACSDiva software (BD Biosciences) and FlowJo analysis.

### Isolation of neutrophils from liver metastases

To isolate neutrophils from liver metastases, 4T1, EMT6.5 or MC38 cells (shSCR or sh*Zdhhc17*) were injected intrasplenically into 6-to 8-week-old female BALB/C mice. After 14 days mice were anaesthetized with an intraperitoneal injection of 10 µl/g containing ketamine (100 mg/kg)-xylazine (10 mg/kg). Livers were perfused, extracted and briefly washed in saline. Metastases were dissected under the microscope and incubated at 37 °C for 45 min with 0.3 mg/ml liberase (Roche) and DNase1 (1 µg/ml) in DMEM/F12 with 5% FBS. The dissociated metastases were quenched with FACS buffer (3% fetal bovine serum in PBS, with 2 mM EDTA), filtered through a 70-µm cell strainer and centrifuged for 5 min at 300*g*. The cell pellet was washed, incubated with ACK lysis buffer (Merck) and strained with a 40 µm cell strainer. The single-cell suspension was counted and resuspended in neutrophil isolation buffer (0.2% FBS in PBS, with 2mM EDTA) at 10^7^ per 90µl. Per 10^7^ cells, 10µl of anti-Ly6G beads (Miltenyi Biotec, 130-120-337) were added and incubated for 15 minutes on ice in the dark. After a 10-minute centrifugation, the cells were loaded on a LS-column and washed for >5 times with isolation buffer to ensure positive selection of Ly6G+ neutrophils. To validate the efficiency of the neutrophil isolation, the isolated cells were processed for flow cytometry analysis. The experiment was continued when the isolation efficiency showed at least >85% purity (Supplementary Data Figure 1a).

### Isolation of neutrophils from blood

To isolate neutrophils from peripheral blood of the mice, 200-500µl full blood was taken from the mice. After red blood cell lysis, the cells were counted, resuspended in neutrophil isolation buffer and anti-Ly6G beads were added for 10 minutes on ice in the dark. After centrifugation, the cells were loaded on a LS-column and washed for >3 times with isolation buffer to ensure positive selection of Ly6G+ neutrophils. To validate the efficiency of the neutrophil isolation, the isolated cells were processed for flow cytometry analysis.

### Isolation of neutrophils from bone marrow

To isolate neutrophils from the bone marrow, the bone marrow was flushed out of the femur and tibia of BALB/c mice. The cell pellet was filtered through a 70-µm cell strainer, counted and resuspended in neutrophil isolation buffer. Neutrophils were isolated using the anti-Ly6G microbeads as mentioned above.

### Killing assay with neutrophils or conditioned media from neutrophils

To assess the killing capacity of neutrophils, the freshly isolated neutrophils from blood or liver metastases were added to 4T1, EMT6.5 or MC38 parental cells at a 10X ratio (10 neutrophils: 1 cancer cell). The neutrophil-cancer cell co-culture was kept for 24-48h at 37°C. In the co-cultures, various treatments were added: laminin-511 (Biolamina, LN511-0502, at 0.5 µg/well), LCN2 (R&D systems, 1857-LC-050, 1µg/well), anti-LCN2 antibody (R&D systems, AF1857-SP, 1:1000). After 48h, floating cancer cells and neutrophils are taken up, as well as the attached cancer cells and processed for flow cytometry analysis. The killing capacity was calculated based on the percentage of alive cancer cells based on the following formula: $100*(1-\frac{\%\;alive\;cancer\;cells\;in\;co-culture\;}{\%\;alive\;cancer\;cells\;in\;monoculture})$. The difference in killing capacity is represented as relative to the control condition (neutrophils from shSCR metastases) (Supplementary Data Figure 1b). To assess the dying cancer cells, the cancer cells and neutrophils were taken up similarly as before and stained with Annexin V/PI according to manufacturer’s instructions.

To create conditioned media from neutrophils, the isolated neutrophils from either shSCR or *shZdhhc17* liver metastases were cultured for 48h in full RPMI. Afterwards, the media was collected, centrifuged for 10 minutes at 300g, and added to the cancer cells for 48h. Killing was assessed in a similar manner as in the other co-cultures. For the degranulation inhibition experiment, the isolated neutrophils were cultured in full RPMI for 48h in the presence of Nexinhib20 (0.5µM) or vehicle.

### Cytokine array

An equal amount of volume of neutrophil conditioned medium was analyzed using the Proteome Profiler Mouse XL Cytokine Array Kit (ARY028, R&D Systems), according to the manufacturer’s instructions. Films were scanned and analyzed using ImageStudio software.

### Haematoxylin and eosin (H&E) staining and histological analysis

H&E staining was performed to identify the cancer metastases lesions in the livers or lungs. For this, liver and lung tissues were fixed overnight in 10% neutral buffered formalin, washed with 70% EtOH and put in the histokinette. After paraffin embedding, 7 µm sections were cut with a microtome, dried overnight at 37°C and subsequently stained using the Autostainer XL (Leica). Scanned slides were analyzed for metastatic area using QuPath (v0.4.4). Representative H&E images for the mice experiments can be found in Extended Data Fig. 10.

### Immunofluorescence (IF)

Formalin fixed, paraffin embedded mouse tissues were sectioned at 7 µm, deparaffinized in xylenes and rehydrated in ethanol/water. For NETosis staining^78^, antigens were retrieved in a Tris-EDTA buffer (10mM Tris-base, 1mM EDTA, 0.05% Tween20, pH 9) in a pressure cooker. The slides were blocked for 1 hour (2,5% BSA, 5% donkey serum, 0.1% Triton-X100 in PBS) and stained overnight at 4°C with primary antibodies in 0.5X of blocking buffer: MPO (R&D Systems, AF3667-SP, 1:200), citrullinated histone H3 (Cell Signaling, 97272S, 1:200), Ly6G for mouse neutrophil identification (BD Bioscience, 1:500), LCN2 (R&D systems, AF1857-SP, 1:200).. The day after, the slides were washed 3 times using PBS and incubated with secondary antibodies (donkey anti-goat 555, donkey anti rabbit 647 and donkey anti rat 488, Thermo Fisher Scientific) for 1h in PBS. At last, the nuclei were stained using DAPI (10 µg/ml) for 10 minutes at room-temperature. All slides were scanned and analyzed using QuPath (v0.4.4). Metastases were identified based on an H&E staining of a consecutive slide. The percentage of NET-releasing neutrophils (double positive for MPO and citrH3) in metastases were quantified using QuPath software. To avoid any bias, similarly sized metastases across conditions were taken for analysis.

### Multiplex immunohistochemistry (IHC) of mouse and patient tissues

For LY6G and CD8a staining, manual IHC was performed. Briefly, slides were deparaffinized and antigens were retrieved (AR6 buffer, Akoya) in a 100°C oven for 20 minutes. After 30 minutes blocking (1% BSA, 10% goat serum in TBS), slides were incubated with primary antibody in TNB for 30 minutes: Ly6G (BD Bioscience, 551459, 1:1000), CD8a (Cell Signaling, 98941, 1:1000). Afterwards, the slides were incubated with a secondary anti-Rabbit-HRP antibody (Akoya) and with Opal 690 (1:150, Akoya) in amplification diluent for each 10 minutes. Antibodies were stripped off the slide using a 90-Watt microwave for 15 minutes and nuclei were stained using DAPI.

Formalin fixed, paraffin embedded human tissues were sectioned at 7 µm, deparaffinized in xylenes and rehydrated in ethanol/water. Subsequently, antigen retrieval was performed using BOND Epitope Retrieval Solution 2, pH 6 (Leica) for 20 minutes at 100 °C. After cooling, slides were blocked with 10% normal goat serum (Thermo Fisher Scientific) in 0.5% BSA in Tris Buffered Saline for 30 minutes at room temperature. Primary antibodies were diluted in Tris-NaCl Blocking buffer (100 mM of Tris-HCl pH 7.5, 150 mM NaCl, 0.5% w/v of blocking reagent (Akoya)): DHHC17 (Proteintech, 30022-1-AP, 1:200), LAMB1 (Proteintech, 23498-1-AP, 1:400), EpCAM (Abcam, 71916, 1:200 dilution), MPO (Abcam, ab9535, 1:500), citrullinated histone H3 (Cell Signaling, 97272S, 1:200), CD15 for human neutrophil identification (Abcam, ab17080, 1:100) and applied to the slides for 60 minutes at room temperature. After washing, the HRP-conjugated anti-rabbit (Dako) or anti-mouse (Akoya) secondary antibody was applied for 10 minutes at room temperature and then the slides were washed again before applying the respectively OPAL fluorophore working solution (Akoya) for 10 minutes. Then, antibodies were heat-stripped in epitope retrieval solution for 15 minutes. Subsequent staining rounds were then performed starting from the blocking step. At the end, the slides were counterstained with 1 ng/mL DAPI (Akoya) for 5 minutes at room temperature and mounted with ImmunMount (Thermo Fisher Scientific). Slides were imaged using the AKOYA PhenoImager and analyzed in QuPath (v0.4.4.). DHHC17 and LAMB1 expression were quantified by calculating the percentage cells positive for DHHC17 or LAMB1 from all DAPI positive cells in several randomly selected metastatic areas and adjacent tissue. For each patient the mean of minimum 3 microscopy fields is shown in the graph, except if the material was limited.

### In vivo experiments

All animal experiments performed in this study complied with ethical regulations and were approved by the Animal Ethics Committee of KU Leuven (ECD number: P007/2020, P048/2021, P106/2023, P028/2024). All mice were housed under a regimen of 12 h light–12 h dark in non-SPF (conventional) conditions with *ad libitum* supply of food and water. Sample size calculations were based on a power of 80% and *P*-value of 0.05 and after correction for multiple testing. For all experiments female BALB/c or C57BL/6NCrl mice were used at an age of 6-8 weeks. For breast cancer studies, only female mice were used while for colorectal cancer studies both female and male C57BL/6NCrl mice were used.

For primary tumor experiments, 4T1 cells were injected in the m.f.p. of the mice (1×10^3^ for the 4T1-7226 clone). Mice were sacrificed and primary tumors were collected after 21 days. For lung metastasis experiments, BALB/c mice were injected *intravenously* with 4T1 cells (1×10^5^ cells) and sacrificed after 13 days. For liver metastases, 4T1 cells were injected *hydrodynamically* (5×10^5^ cells) in the tail vein of BALB/c mice, or *intrasplenically* (2.5×10^5^ for 4T1 and 2×10^5^ for EMT6.5 cells) in BALB/c or (5×10^5^ for MC38) in C57BL/6NCrl mice. During the intrasplenic injection, the spleen was removed surgically 2 minutes after cancer injection. The mice were sacrificed and livers were collected either 10 days (hydrodynamic) or 14 days (intrasplenic) after injection.

For neutrophil depletion experiments, rat anti-Ly6G (BioXCell BE0075, clone 1A8, 12.5 µg/mouse, in PBS) or rat IgG isotype control (BioXCell BE0089, 12.5 µg/mouse, in PBS) were intraperitoneally injected daily to BALB/c mice^27^. To deplete neutrophil in C57BL/6NCrl mice, the Combo method was used^26^. The mice were daily intraperitoneally injected with rat anti-Ly6G (25 µg/mouse in the first week, 50µg/mouse in the second week, in PBS) or rat IgG isotype control (25 µg/mouse in the first week, 50µg/mouse in the second week, in PBS). Every other day, the mice were also injected intraperitoneally with anti-rat kappa light antibody (50µg/mouse in PBS, BioXCell, BE0122) or control (50µg/mouse in PBS, BioXCell, BE0085). To inhibit neutrophil degranulation, female Balb/c or C57BL/6NCrl mice were given an intraperitoneal injection of Nexinhib20 (30 mg/kg) or a vehicle, for 3 times per week. Injections for neutrophil depletion or degranulation inhibition were started 24h after cancer cell injection. For CD8a depletion experiment, anti-CD8a (BioXCell, 10mg/kg in PBS) or vehicle was intraperitoneally injected three days prior injection, one day after injection and then weekly. Depletion efficacy was confirmed using FACS analysis.

For the HFD experiments, 4-week-old female BALB/c or C57BL/6NCrl mice were randomized into two groups: CD (E15742-33 ssniff Spezialdiäten) or long-chain HFD (S8655-E220 sniff Spezialdiäten). The specific energy balance from fat/protein/carbohydrates for these diets are for high fat diet: 60:20:20% and control diet: 13:27:60%. Mice were maintained on these diets for 16 (BALB/c) or 12 weeks (C57BL/6NCrl) before the experiments were performed. Body weight was monitored and mice were inspected weekly for welfare assessment. For liver metastasis, 2×10^5^ cells were injected intrasplenically and mice were sacrificed after 12 days. To deplete the neutrophils efficiently in the HFD model, rat anti-Ly6G (12.5 µg/mouse or rat IgG isotype control (12.5 µg/mouse) were intraperitoneally injected daily to the HFD BALB/c mice during the first week and concentration was doubled during the second week after cancer cell injection. For CD8a depletion in the HFD mice, anti-CD8a was given as written above. In none of the animal experiments, the maximal tumor burden (1.8cm^3^) was exceeded.

### Patient sample acquisition

Paraffin embedded and fresh frozen tissue samples from liver metastases and primary tumors were obtained through the ethically approved UPTIDER program (KU/UZ Leuven Program for Postmortem Tissue Donation to Enhance Research, NCT04531696, S64410). Patients with metastatic breast cancer who decided to participate based on an informed consent in UPTIDER were included in our study. Protein extraction was performed from fresh frozen tissue samples and IHC was performed on paraffin embedded tissues. Clinicopathological information for every patient in the UPTIDER dataset is shown in Supplementary Table 1.

Formalin fixed and paraffin embedded tissue samples from colorectal cancer liver metastases were obtained through the Department of Oncological Surgery and Department of Pathology of the Erasmus MC Rotterdam (study number MEC-2018-1743). The analysis of patient tissues is approved by the local KU/UZ Leuven Medical Ethics Committee (study number S67925). All tissues originated from patients who underwent a metastasectomy operation of colorectal cancer liver metastases in a curative intent. Clinicopathological information for every patient in this dataset is shown in Supplementary Table 2.

### Palmitate injection experiment

To locally increase palmitate levels in the liver, we dissolved ethyl palmitate at 200mM in 4.8% lecithin and 10% glycerol^79^. The lecithin-glycerol-water solution was used as vehicle for the mice. To prime the liver before cancer cell injection, 100 µl of this mix was injected 3x per week (Monday, Wednesday and Friday) directly in the liver using ultrasound (Vevo 3000). The increase in palmitate levels in the liver was confirmed by assessing the liver interstitial fluid. After two weeks, 4T1 cells were *intrasplenically* injected to induce liver metastases. 72h after the surgery, we again injected ethyl palmitate in the livers until the end of the experiment.

### Isolation of liver interstitial fluid from livers and liver metastases

Mice were sacrificed by *intraperitoneal* injection of pentobarbital (Dolethal) (50µl of 60mg/ml) and the liver was collected by surgical resection. Liver metastases were resected by eye with the use of a microscope. Liver tissues were washed with saline, dried on a gauze compress and placed in a homemade filtered centrifuge tube with a nylon mesh filter with 20 µm opening pores (Repligen). The interstitial fluid was collected by gentle centrifugation (10 minutes at 400g, 4°C).

### Mass spectrometry analysis

Metabolites were immediately extracted from the interstitial fluid^11,75^. For fatty acid measurements of the interstitial fluid, the lipid fraction was esterified with 2% sulfuric acid in methanol overnight at 50 °C and separated with GC (8860 or 7890A GC system, Agilent Technologies) combined with MS (5977B or 5975C Inert MS system, Agilent Technologies). Then, 1 μL was injected in splitless mode with an inlet temperature of 270 °C on a DB-FASTFAME column (30 m × 0.250 mm). Helium was used as a carrier gas with a flow rate of 1 ml min−1. The initial gradient temperature was set at 50 °C for 1 min and increased at the ramping rate of 12 °C min−1 to 180 °C, followed by a ramping rate of 1 °C min−1 to reach 200 °C for 1 min. The final gradient temperature was set at 230 °C with a ramping rate of 5 °C min−1 for 2 min and the temperatures of the quadrupole and the source at 150 °C and 230 °C, respectively. The MS system was operated under electron impact ionization at 70 eV (70–700 amu mass range). Metabolite abundances were calculated from the raw chromatograms using a homemade MATLAB script. For relative abundance, total ion counts were normalized to the internal standard and the extracted volume. For the proteome and palmitoylome, digested peptides were dissolved in 0.1% formic acid and 3% acetonitrile and loaded onto a PepMap100 C18 trap column (300µm × 5mm, 5µm beads; Thermo Fisher Scientific) and separated with a NanoEase MZ HSS T3 analytical column (75 μm × 250 mm, 1.8 μm beads; Waters) using a Dionex Ultimate 3000 nano liquid chromatographic system (Thermo Fisher Scientific) at a flow rate of 250 nl/min. Separation comprised three consecutive linear gradients: from 3 to 35% B in 180 min, from 35 to 50% B in 5 min and from 50 to 85% B in 2 min (*A*=0.1% FA in water, *B*=0.1% FA in CH_3_CN). The column outlet was connected to an Advion TriVersa NanoMate (Advion) with spray voltage set to 1.70 kV and a delivery pressure of 0.5 psi. Peptides were directly eluted into a Orbitrap Fusion Lumos™ Tribrid (Thermo Fisher Scientific) mass spectrometer, which was operated in positive mode using the data-dependent acquisition mode and singly charge state precursors were rejected for fragmentation. For proteome samples, survey MS scans were acquired in the Orbitrap with a scan range of 375 to 1,500 m/z using a 1.6-m/z precursor isolation window and with the resolution set to 120,000 and normalized automatic gain control (AGC) target of 100%. In each Orbitrap scan the top speed (most intense) ions per scan were fragmented by collision-induced dissociation (CID) with 35% of collision energy and detected in the linear ion trap. The ion count target value was 400,000 and 10,000 for the survey scan and for the MS/MS scan respectively. Target ions already selected for MS/MS were dynamically excluded for 15s. RF Lens were tuned to 30%. MS conditions for palmitoyl-proteome samples varied in the following terms: the m/z range was set to 350-1400 m/z, dynamic exclusion to 10s and higher energy collisional dissociation (HCD) was used as fragmentation method with a collision energy of 28%, and fragmented peptides were detected in the orbitrap with the resolution set to 60,000, maximum injection time of 150 ms and normalized AGC target of 150%. In both cases, Xcalibur version 4.2.28.14 (Thermo Fisher Scientific) was used to operate the instrument.

### Bulk RNA-sequencing of liver metastases

Female BALB/C mice (6-8 weeks of age) were *hydrodynamically* injected with 4T1 sh*Zdhhc17* or shSCR2 (*n* = 3 each). After 10 days, the mice were anaesthetised with an intraperitoneal injection of Dolethal. Liver metastases were dissected with the help of a microscope and RNA was isolated using Trizol reagent (Thermo Fisher Scientific). RNA concentration and purity were determined using the Nanodrop ND-8000 (Nanodrop Technologies) and RNA integrity and concentration were assessed using the Bionalyzer RNA Nano kit (Agilent). Per sample, 500 ng of total RNA was used as input for RNA-sequencing library preparation. Using the Illumina Stranded mRNA Sample Prep Kit (protocol version: # 1000000124518 v02 (April 2021)) poly-A containing mRNA molecules were purified from the total RNA input using poly-dT oligo-attached magnetic beads. The purified mRNA was then fragmented, converted into first strand cDNA in a reverse transcription reaction using random primers and Actinomycin D, and subsequently converted into double-stranded cDNA in a second strand cDNA synthesis reaction using dUTP to achieve strand specificity. The cDNA fragments were extended with a single ‘A’ base to the 3’ ends of the blunt-ended cDNA fragments after which pre-index anchors were ligated preparing the fragments for dual indexing. Anchor-ligated fragments were then purified using magnetic beads. Finally, enrichment PCR was carried out to enrich those DNA fragments that have anchor-ligated DNA fragments and to add indexes and primer sequences for cluster generation. Purified dual-indexed sequence-libraries of each sample were equimolarly pooled and sequenced on an Illumina NovaSeq 6000 (v1.5 kit, single read 100 (100-10-10-0)) at the VIB Nucleomics Core (www.nucleomics.be).

Following demultiplexing, reads for each sample originating from different sequencing lanes were concatenated, and the resulting merged reads were subject to optical-duplicate removal and adapter trimming using the *BBMap* suite (v38.98; https://sourceforge.net/projects/bbmap) modules *clumpify* and *BBDuk*, respectively. Trimmed-read quality control was then performed using FastQC (v0.11.8; https://www.bioinformatics.babraham.ac.uk/projects/fastqc). High-quality reads were then mapped to the mm39 reference mouse genome (GRCm39, Gencode build vM30) using *STAR*^80^ (v2.6.1; https://github.com/alexdobin/STAR), and further quantified using *HTSeq’s*^81^ (v0.9.1; https://htseq.readthedocs.io) *htseq*-count module. All the above steps were carried out using the VSC (Flemish Supercomputer Center) infrastructure, funded by the Research Foundation – Flanders (FWO) and the Flemish Government.

Gene counts for all 6 samples collected under the 2 conditions of interest (shSCR2 and sh*Zdhhc17*) were first filtered to keep only genes expressed in at least 2 out of 3 samples for at least one of the two conditions. Differential expression analysis was then performed within the *DESeq2* (v1.34.0; https://bioconductor.org/packages/release/bioc/html/DESeq2.html) framework^82^ on the remaining (24167) genes in order to determine differentially expressed genes between sh*Zdhhc17* and shSCR2 metastases. Differential expression analysis results corresponding to known marker genes for different immune-cell populations were projected onto the resulting Volcano plot so as to assess changes between the immune compartments of these metastases. DESeq2-derived log fold-changes (log_2_FC) and *P*-values for all genes in the sh*Zdhhc17* versus shSCR2 contrast were further combined to derive a ranking metric of the form RM = -log_10_(*P*-value) × log_2_FC. Pre-ranked gene-set enrichment analysis (GSEA;(https://www.gsea-msigdb.org/gsea) was then performed based on this ranking metric, using the R package *fgsea*^83^ (v1.20.0; https://github.com/ctlab/fgsea; *fgsea*-multilevel implementation with 10000 initial permutations and no lower bound for *P*-value estimation), and considering a collection of 3065 mouse gene sets. The latter comprised all HALLMARK and CURATED (C2) gene sets in the Broad Institute Molecular Signatures Database (MSigDB^84,85^; https://www.gsea-msigdb.org/gsea/msigdb) except for those in category C2:CGP, all of them obtained using the R package *msigdbr* (v7.4.1; https://igordot.github.io/msigdbr), plus all KEGG^86^ (https://www.genome.jp/kegg) metabolism gene sets, obtained by querying KEGG’s REST API (https://www.kegg.jp/kegg/rest). Only gene sets containing between 3 and 1500 genes (out of those with available differential expression analysis results) were considered for GSEA. Gene sets with either positive/negative (enriched/depleted in sh*Zdhhc17* versus shSCR metastases, respectively) normalized enrichment scores (NES) were ranked based on their absolute NES, with the top 10 gene sets for each direction being shown in the manuscript.

### Single-cell RNA-sequencing of liver metastases

Female BALB/C mice (6-8 weeks of age) were *intrasplenically* injected with 4T1 shZdhhc17 or shSCR (*n* = 3 each). After 14 days, mice were anaesthetised with an intraperitoneal injection of 10 µl/g containing ketamine (100 mg/kg) and xylazine (10 mg/kg). The liver was perfused and liver metastases were dissected using the microscope. Metastases were dissociated, after which the same number of cells were pooled together from the 3 independent liver metastases dissociations performed per group, resuspended in cell culture medium and loaded onto a BD Rhapsody (BD Biosciences) cartridge with a targeted capture of 15 000 cells per group. The remainder of the single-cell suspension was processed in parallel for flow cytometry analysis. Reverse transcription, cDNA amplification and library construction were performed following the manufacturer’s instructions (23-24117(02)). Libraries were first subject to shallow sequencing on an Illumina NextSeq2000 (P3 flow cell, 51-8-0-90), and after quality check were sequenced deeper on an Illumina NovaSeq6000 (S2 flow cell, 51-8-0-90), all at the VIB Nucleomics Core (www.nucleomics.be).

The BD Rhapsody Sequence Analysis Pipeline (BD Biosciences; version 2.0) was used to map the FASTQ files generated upon sequencing to the GRCm38 mouse reference genome. The resulting single-cell gene expression data were analysed within the *R/Bioconductor* framework (www.r-project.org and www.bioconductor.org). Raw unique molecular identifier (UMI) count matrices for both samples (shSCR and sh*Zdhhc17*) were first imported using *Seurat* (v4.1.0)^87^ (www.satijalab.org/seurat), and immediately subject to ambient RNA correction on a per-sample basis, using a customized version of the *SoupX* (v1.6.2) *R* pipeline (https://github.com/constantAmateur/SoupX)^88^. Specifically, two modifications were applied to the standard *SoupX* pipeline. First, the automatically determined (global) ambient contamination fractions for each of the samples were multiplicatively increased by a factor of 5, to account for the fact that, as reported in the literature, the automatic determination method tends to underestimate ambient contamination levels for single-cell RNA-sequencing data^89^. Second, rather than assuming a fixed contamination fraction for all cells, the latter was adapted for each cell based on its specific library size (total UMI counts), to achieve a uniform distribution of subtracted counts across all cells in each sample, in keeping with the nature of the ambient RNA contamination problem^89^. Ambient-corrected count matrices for the two samples were then merged together and converted for further processing with *Monocle3-alpha* (v2.99.3)^90^ (www.github.com/cole-trapnell-lab/monocle-release/tree/monocle3_alpha). Low-quality cells were filtered out based on standard quality-control metrics, with thresholds chosen based on evaluating quality-control histograms for the merged dataset. In particular, cells were filtered based on their mitochondrial RNA content (allowing for a maximum of 15%), library size (removing cells with total UMI counts below 500), and number of detected genes (removing cells expressing fewer than 250 genes). Genes not expressed in any of the cells remaining after quality-control filtering were ignored in all subsequent analyses. Size-factor normalization and variance-stabilizing transformation (based on fitting to a negative binomial distribution) were then applied to the filtered dataset, and highly variable genes (HVGs) were identified based on their departure from the average normalized dispersion versus expression trend observed among all genes. After excluding mitochondrial, ribosomal protein, and cell cycle-associated genes, the top 1,000 HVGs with size-factor normalized expression levels above 0.02 were selected. Principal component analysis (PCA) was then performed on the size factor-normalized and variance-stabilized count matrix restricted to these genes only, followed by 2D uniform manifold approximation and projection (UMAP^91^) dimensional reduction based on the resulting top 50 principal components (with *correlation distance metric, number of neighbors* = 15, and *minimum distance* = 0.1). After that, cells were clustered in the UMAP plane by applying *Monocle3-alpha*’s implementation of the *Louvain*^92^ graph-based algorithm at high resolution (*resolution* = 0.001, with *k*_NN_ = 9), in order to attain a fine-grained cluster structure (138 clusters). The resulting fine-grained clusters were then manually annotated to specific cell types, based on evaluating the expression profiles of several cell type-specific markers across the different fine-grained clusters. These cell-type annotations were further refined based on a second (analogous) step of dimensional reduction and clustering, applied separately to each of the preliminarily annotated cell-types. This in turn allowed us to more easily identify and filter out ambiguous clusters, most notably those originating from heterogeneous cell multiplets, characterized by simultaneously presenting markers associated to two or more cell types. Our multiplet identification was confirmed based on the distribution of doublet scores estimated across the different cells in each separate sample using the *R* package *scDblFinder* (v1.8.0)^93^ (https://bioconductor.org/packages/release/bioc/html/scDblFinder.html).

Specifically for the case of neutrophils, the latter were identified upon the first (global) clustering step based on expression of the markers *S100a8, S100a9, Ly6g*, and *Retnlg*, with ~ 9% of the cells initially annotated as neutrophils being re-annotated as potential multiplets (and thus excluded from all subsequent analyses) upon neutrophil-specific clustering refinement. Formal sub-clustering was then performed on the remaining neutrophils by applying unsupervised clustering in the PCA space, based again on *Monocle3-alpha*’s implementation of the *Louvain* algorithm, but now with default resolution/*k*_NN_ parameters. The latter resulted in the identification of 9 different neutrophil clusters or *subsets*. Marker genes for each of these subsets were inferred within the *Seurat* framework, using the function *FindMarkers* (with default parameters other than not imposing a fold-change threshold for output gene filtering) to compare each of the 9 subsets to the combination of all other subsets. In brief, the output of *FindMarkers* for each subset *S* was first filtered to include only genes enriched in that subset relative to the combination of all other subsets (log_2_FC_S vs REST_ > 0). A specificity score for that subset was then calculated for each of the remaining genes as *SPEC*_*S*_ *= X*_*S*_ × *(1 – X*_*REST*_ */2)*, where *X*_*S*_ and *X*_*REST*_ represent the fraction of cells expressing that gene within subset *S* and all other subsets, respectively. Genes were then ranked within each subset based on a combined marker score, defined as the product *MS*_*S*_ = log_2_FC_S vs REST_ × *SPEC*_*S*_ for each gene, with the top ranked genes for each subset being considered as potential markers. To ensure uniqueness among the markers assigned to different subsets, the list of potential markers for each subset was further filtered to include only genes presenting a higher marker score *MS*_*S*_ in that subset relative to all other subsets. The top 10 (unique) markers for each subset remaining after this filtering step are shown in the manuscript, and the top 20 were used to annotate the different neutrophil subsets to specific phenotypes based on literature search^94^. For example, markers like *Mpo, Elane* and *Fcnb* were used to annotate phenotype “Early-Stage”^95,96^, whereas markers such as *Hilpda, Ero1l* and *Slc2a1* were used to annotate phenotype Hypoxia/Angiogenesis^28^. Subsets showing a substantial overlap (> 50%) between their top 100 potential markers (before imposing the marker-uniqueness constraint) were in turn annotated to the same phenotype (e.g. Early-Stage, NETosis, Migration).

### Statistical analysis

Statistical data analysis was performed using GraphPad Prism v.8 (GraphPad Software) on n ≥ 3 biological replicates. Data are presented as mean (± SD). Details of statistical tests are mentioned in figure legends. Where not specifically indicated, non-significant comparisons are not shown. Mathematical outliers were determined using the Grubb’s outlier test (GraphPad) with alpa = 0.05 and consequently excluded for final analysis. For all mice experiments, normality and variance were formally tested. Animals were only excluded in case they died before the experimental endpoint or had to be killed according to ethical protocols. For mice experiments, all animals were randomized for diet, treatment or injection group. Mice were assigned a unique number before data collection to ensure blinded analysis. Also, for the in vitro studies, samples were randomized when possible before data acquisition. For these experiments, data distribution was assumed to be normally distributed. Statistical analysis for all time-course profiles was based on linear regression of the data to polynomials of the specified degree in the continuous time variable, either assuming joint coefficients for all conditions being compared (null model), or letting the coefficients depend on the condition (complete model). Statistical comparisons between the complete and null models were then performed using F-Tests, to assess the existence of significant differences between the time-course profiles for the different conditions. Polynomial degrees for regression were chosen empirically for each data set to ensure goodness of fit, and unless otherwise noted were set to at most 2 degrees lower than the number of time points in the data set, to avoid overfitting.

### Materials availability

This study did not generate new unique reagents, except of genetically manipulated cell lines based on commercially available constructs. Reagents generated in this study will be made available on request through the lead author or the collaboration partner that generated the resource, but we may require a payment and/or a completed Materials Transfer Agreement if there is potential for commercial application.

## Extended Data

**Extended Data Fig. 9:**
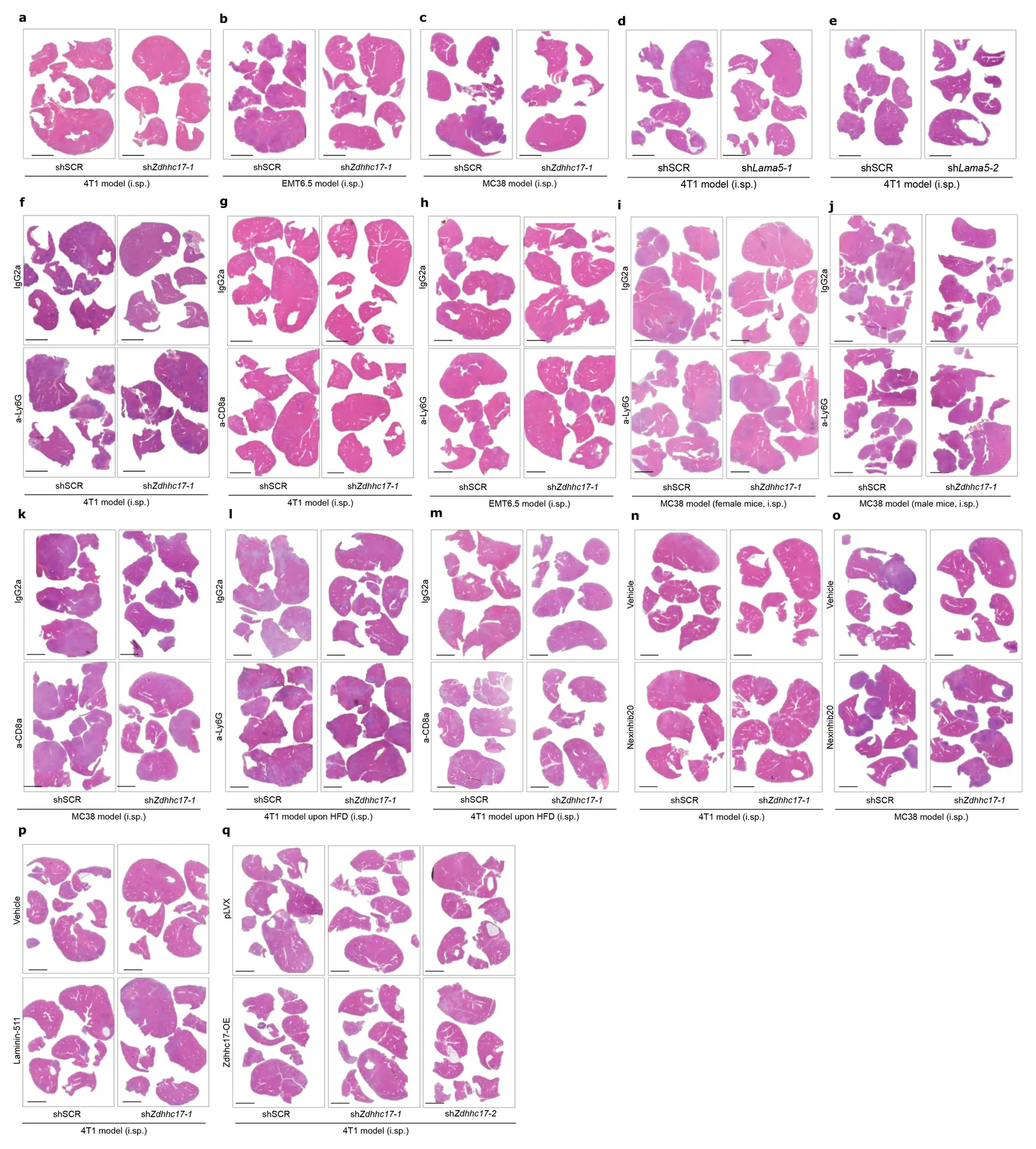
Representative H&E staining in mouse metastasis models a. Representative H&E staining in livers of mice *intrasplenically* injected with shSCR or sh*Zdhhc17-1* 4T1 cells. Scale bar 5 mm. b. Representative H&E staining in livers of mice *intrasplenically* injected with shSCR or sh*Zdhhc17-1* EMT6.5 cells. Scale bar 5 mm. c. Representative H&E staining in livers of mice *intrasplenically* injected with shSCR or sh*Zdhhc17-1* MC38 cells. Scale bar 5 mm. d. Representative H&E staining in livers of mice *intrasplenically* injected with shSCR or sh*Lama5-1* 4T1 cells. Scale bar 5 mm. e. Representative H&E staining in livers of mice *intrasplenically* injected with shSCR or sh*Lama5-2* 4T1cells. Scale bar 5 mm. f. Representative H&E staining in livers of mice *intrasplenically* injected with shSCR or sh*Zdhhc17-1* 4T1 cells and 24h later treated daily with IgG2A or α-Ly6G antibody. Scale bar 5 mm g. Representative H&E staining in livers of mice *intrasplenically* injected with shSCR or sh*Zdhhc17-1* 4T1 cells, treated weekly with IgG2A or α-CD8a antibody. Scale bar 5 mm. h. Representative H&E staining in livers of mice *intrasplenically* injected with shSCR or sh*Zdhhc17-1* EMT6.5 cells and 24h later, treated daily with IgG2A or α-Ly6G antibody. Scale bar 5 mm. i. Representative H&E staining in livers of female mice *intrasplenically* injected with shSCR or sh*Zdhhc17-1* MC38 cells and 24h later treated daily with IgG2A or α-Ly6G antibody. Scale bar 5 mm. j. Representative H&E staining in livers of male mice *intrasplenically* injected with shSCR or sh*Zdhhc17-1* MC38 cells and 24h later treated daily with IgG2A or α-Ly6G antibody. Scale bar 5 mm. k. Representative H&E staining in livers of mice *intrasplenically* injected with shSCR or sh*Zdhhc17-1* MC38 cells, treated weekly with IgG2A or α-CD8a. Scale bar 5 mm. l. Representative H&E staining in livers of mice fed with HFD for 16 weeks and *intrasplenically* injected with shSCR or sh*Zdhhc17-1* 4T1 cells. The day after, the mice were treated with IgG2A or α-Ly6G antibody. Scale bar 5 mm. m. Representative H&E staining in livers of mice fed with HFD for 16 weeks and *intrasplenically* injected with shSCR or sh*Zdhhc17-1* 4T1 cells, treated weekly with IgG2A or α-CD8a antibody. Scale bar 5 mm. n. Representative H&E staining in livers of mice *intrasplenically* injected with shSCR or sh*Zdhhc17-1* 4T1 cells, treated 3x/week with vehicle or Nexinhib20 (30 mg/kg). Scale bar 5 mm. o. Representative H&E staining in livers of mice *intrasplenically* injected with shSCR or sh*Zdhhc17-1* MC38 cells, treated 3x/week with vehicle or Nexinhib20 (30 mg/kg). Scale bar 5 mm. p. Representative H&E staining in livers of mice *intrasplenically* injected with shSCR or sh*Zdhhc17-1* 4T1 cells, injected with vehicle or LAMININ-511 directly in the liver. Scale bar 5 mm. q. Representative H&E staining in livers of mice *intrasplenically* injected with shSCR or sh*Zdhhc17-1*, sh*Zdhhc17-2*, shSCR -OE, *shZdhhc17-1-*OE, *shZdhhc17-2-*OE 4T1 cells. Scale bar 5 mm.

**Extended Data Fig. 10:**
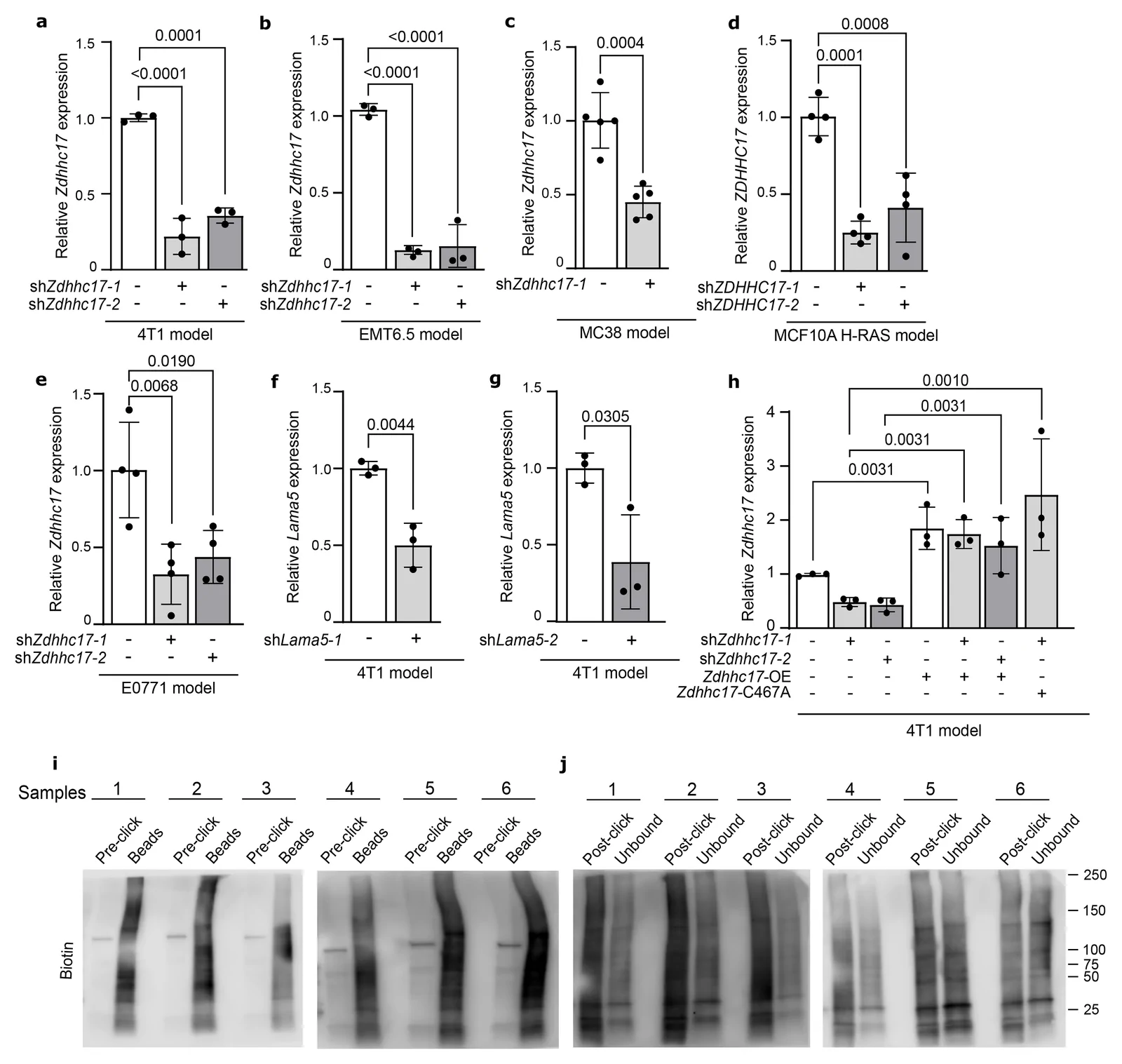
mRNA expression of genetically modified breast and colorectal cancer cells a. mRNA expression of *Zdhhc17* in 4T1 shSCR, sh*Zdhhc17-1*, and sh*Zdhhc17-2* cells (n=3 biological replicates). 1-way ANOVA. b. mRNA expression of *Zdhhc17* in EMT6.5 shSCR, sh*Zdhhc17-1* and sh*Zdhhc17-2* cells (n=3 biological replicates). 1-way ANOVA. c. mRNA expression of *Zdhhc17* in MC38 shSCR andsh*Zdhhc17-1* cells (n=5 biological replicates). Two-tailed unpaired t-test. d. mRNA expression of *ZDHHC17* in MCF10 H-RAS^V12^ shSCR, sh*ZDHHC17-1* and sh*ZDHHC17-2* cells (n=4 biological replicates). 1-way ANOVA. e. mRNA expression of *Zdhhc17* in E0771 shSCR, sh*Zdhhc17-1* and sh*Zdhhc17-2* cells (n=4 biological replicates). 1-way ANOVA. f. mRNA expression of *Lama5* in 4T1 shSCR and sh*Lama5-1* cells (n=3 biological replicates). Two-tailed unpaired t-test. g. mRNA expression of *Lama5* in 4T1 shSCR and sh*Lama5-2* cells (n=3 biological replicates). Two-tailed unpaired t-test. h. mRNA expression of *Zdhhc17* in 4T1 shSCR, *shZdhhc17-1* and *shZdhhc17-2* cells with overexpression of wild-type *Zdhhc17* or catalytic mutant (C467A) *Zdhhc17 (*n=3 biological replicates*)*. 2-way ANOVA. i. Protein levels of biotin at “pre-click” stage of the click-chemistry versus the “beads” (= palmitoylated proteins) in the 6 biological replicates (3 independent experiments) of the palmitoylome proteomics. j. Protein levels of biotin at “post-click” stage of the click-chemistry versus the “unbound” (= non-palmitoylated proteins) in the 6 biological replicates (3 independent experiments) of the palmitoylome proteomics. Data are mean ± s.d. unless indicated otherwise.

## Supplementary Material

Reporting Summary

Supplementary Data Figure 1

SUPPLEMENTARY TABLES

## Data Availability

Mouse single-cell RNA sequencing data and bulk RNA sequencing data generated as part of this study have been deposited in the Gene Expression Omnibus (GEO) under accession GSE300661. Source data are provided with this paper.

## Code Availability

No original software and/or algorithms were developed in the present study; however, code used for data analysis can be provided upon request.
