# Supplementary material for: Palmitate promotes liver metastases by decreasing neutrophil antitumour behaviour
Source: Nat Metab. Author manuscript; Available in PMC 2026 Oct 17. (PMC7619559; doi:10.1038/s42255-026-01582-0)
Supplement: Supplementary file 1 [file EMS217595-supplement-Reporting_Summary.pdf]

nature portfolio

Corresponding author(s): Sarah-Maria Fendt

Last updated by author(s): Jun 25, 2026

Reporting Summary

Nature Portfolio wishes to improve the reproducibility of the work that we publish. This form provides structure for consistency and transparency in reporting. For further information on Nature Portfolio policies, see our Editorial Policies and the Editorial Policy Checklist.

Statistics

For all statistical analyses, confirm that the following items are present in the figure legend, table legend, main text, or Methods section.

n/a	Confirmed	
☐	☒	The exact sample size (*n*) for each experimental group/condition, given as a discrete number and unit of measurement
☐	☒	A statement on whether measurements were taken from distinct samples or whether the same sample was measured repeatedly
☐	☒	The statistical test(s) used AND whether they are one- or two-sided<br>*Only common tests should be described solely by name; describe more complex techniques in the Methods section.*
☒	☐	A description of all covariates tested
☒	☐	A description of any assumptions or corrections, such as tests of normality and adjustment for multiple comparisons
☐	☒	A full description of the statistical parameters including central tendency (e.g. means) or other basic estimates (e.g. regression coefficient) AND variation (e.g. standard deviation) or associated estimates of uncertainty (e.g. confidence intervals)
☐	☒	For null hypothesis testing, the test statistic (e.g. *F*, *t*, *r*) with confidence intervals, effect sizes, degrees of freedom and *P* value noted<br>*Give P values as exact values whenever suitable.*
☒	☐	For Bayesian analysis, information on the choice of priors and Markov chain Monte Carlo settings
☒	☐	For hierarchical and complex designs, identification of the appropriate level for tests and full reporting of outcomes
☒	☐	Estimates of effect sizes (e.g. Cohen's *d*, Pearson's *r*), indicating how they were calculated

Our web collection on statistics for biologists contains articles on many of the points above.

Software and code

Policy information about availability of computer code

Data collection	GCMS data was collected using MSD Chemstation Data Analysis software F.01.03.2365 (Agilent). H&E stainings and immunohistochemistry on tissue samples were acquired on the Vectra Polaris/PhenoImager HT 2.0.0 (Akoya). Western blot imaging was performed using an ImageQuant LAS 4000 (GE Healthcare) or a ChemiDoc MP Imaging System (BioRad). qPCR data were acquired using QuantStudio 3 device (Thermo Fisher). Sequencing data were obtained on a Illumina NovaSeq 6000 and a Illumina NextSeq2000 system (Illumina). Flow cytometry data were acquired using the BD FACS Diva software v9.0 (BD Biosciences)
Data analysis	H&E staining and immunohistochemistry data were analyzed on QuPath (5.1). qPCR data were analyzed using QuantStudio 3 (1.5.2). Western Blot data were analyzed using ImageJ/FIJI (1.54f) or ImageStudio. Flow cytometry data were analyzed using FlowJo (v10.8.1). For mass spectrometry analysis, mass isotopomer abundances and isotopologue distributions were extracted from the raw ion chromatograms using Matlab (R2021b). All statistical analyses were performed in GraphPad Prism v.10 or within the R framework.<br>For the RNA-sequencing analysis, sequenced reads were concentrated, were subject to optical-duplicate removal and trimmed for adaptors and low-quality base calls using BBmap (v38.98), after which quality control was performed with FastQC (v0.11.8). High-quality reads were then mapped to the mm39 reference mouse genome (GRCm39) using STAR (v2.6.1) and quantified using HTSeq's (v0.9.1). All these steps were carried out using the VSC (Flemish Supercomputer Center) infrastructure. Gene counts for individual total RNA samples (for total RNA sequencing) collected under all conditions of interest were processed simultaneously within the DESeq2 (v1.34.0) framework. Pre-ranked gene-set enrichment analysis (GSEA) was performed on the output of DESeq2 using the R package fgsea (v1.20.0), considering a collection of 3065 mouse gene sets obtained via the R package msigdbr (v7.4.1) and by querying KEGG's REST API.<br>For single-cell RNA-sequencing analysis, sequenced reads were mapped to a customized version of the GRCm38 mouse reference genome, using the BD Rhapsody Sequence Analysis Pipeline (BD Biosciences). The resulting single-cell gene expression data were analyzed within the R/Bioconductor framework. Raw UMI count matrices for all samples were first imported using Seurat (v4.1.0) and subject to ambient RNA correction using a customized version of SoupX (v1.6.2). Ambient-corrected count matrices for all individual samples were then merged and

converted for further processing with Monocle3-alpha (v2.99.3). Low-quality cells were then filtered out based on standard quality-control metrics, and size-factor and variance-stabilizing normalization were then applied to the filtered data set. Highly variable genes (HVGs) were identified based on their departure from the average normalized dispersion versus expression trend observed among all genes, and principal component analysis (PCA) was then performed on the size factor-normalized and variance-stabilized count matrix restricted to these HVGs, followed by 2D UMAP dimensional reduction. After that, cells were clustered in the UMAP plane by applying Monocle3-alpha implementation of the Louvain graph-based algorithm at high resolution. The resulting fine-grained clusters were then manually annotated to specific cell types, based on evaluating the expression profiles of several cell type-specific markers. This annotation was further refined based on subsequent sub-clustering applied to each of the preliminarily annotated cell types, concomitantly with doublet removal guided by the output of scDblFinder (v1.8.0). Specifically for the case of neutrophils, the latter were identified upon the firstclustering step based on expression of the markers S100a8, S100a9, Ly6g, and Retnlg, with ~ 9% of the cells being re-annotated as potential multiplets upon neutrophil-specific clustering refinement. Formal sub-clustering was then performed on the remaining neutrophils by applying unsupervised clustering in the PCA space, based again on Monocle3-alpha's implementation of the Louvain algorithm, but now with default resolution/kNN parameters. The latter resulted in the identification of 9 different neutrophil clusters or subsets. Marker genes for each of these subsets were inferred within the Seurat framework, using the function FindMarkers.

For manuscripts utilizing custom algorithms or software that are central to the research but not yet described in published literature, software must be made available to editors and reviewers. We strongly encourage code deposition in a community repository (e.g. GitHub). See the Nature Portfolio guidelines for submitting code & software for further information.

Data

Policy information about availability of data

All manuscripts must include a data availability statement. This statement should provide the following information, where applicable:

- Accession codes, unique identifiers, or web links for publicly available datasets
- A description of any restrictions on data availability
- For clinical datasets or third party data, please ensure that the statement adheres to our policy

The authors declare that all the other data supporting the findings of this study are available within the paper and its supplementary information files, and from the corresponding author upon reasonable request. Gel source images are available in Source Data for each Figure/Extended Data Figure.
Mouse single-cell RNA-sequencing data and total RNA sequencing data is deposited in the Gene Expression Omnibus (GEO) under accession code GSE300661.
No original softwares and/or algorithms were developed in the present study, but the code used for data analysis can be provided upon request. All other data supporting the findings of this study are available within the Article and the Supplementary Information, and from the corresponding author on reasonable request.

Research involving human participants, their data, or biological material

Policy information about studies with human participants or human data. See also policy information about sex, gender (identity/presentation), and sexual orientation and race, ethnicity and racism.

Reporting on sex and gender	Liver metastasis tissues from female breast cancer patients and from male or female colorectal cancer patients were analyzed in this study. The gender was not used in the selection of the patient tissue that is analyzed in this manuscript.
Reporting on race, ethnicity, or other socially relevant groupings	Not applicable for this study since no such data was collected as part of the retrospective database.
Population characteristics	Patients with metastatic breast cancer, or hereditary cancer syndrome with a moderate to high lifetime risk of breast cancer, for which the patient is treated/followed in UZ Leuven (UPTIDER). Age of the patients had to be ≥ 18 years.<br>Also samples from colorectal cancer patients who underwent a curative hepatic metastasectomy at the Erasmus MC Rotterdam were included in this study.
Recruitment	Female patients (≥ 18 years) with metastatic breast cancer who consented to participate and undergone a rapid research autopsy in the first 12 hours after death (UPTIDER).<br>Samples and patient characteristics were obtained from the pathological database at the Erasmus MC Rotterdam. These samples were taken in a curative intend and the patients signed an informed consent form. For analyses of patient samples, samples were chosen randomly from the suitable specimens.
Ethics oversight	UZ/KU Leuven Program for Post-mortem Tissue Donation to Enhance Research (UPTIDER), NCT04531696, protocol S64410 and UZ/KU Leuven, protocol S57123.<br>Erasmus MC Rotterdam study number MEC-2018-1743, KU/UZ-Leuven study number S67925

Note that full information on the approval of the study protocol must also be provided in the manuscript.

Field-specific reporting

Please select the one below that is the best fit for your research. If you are not sure, read the appropriate sections before making your selection.

☒ Life sciences ☐ Behavioural & social sciences ☐ Ecological, evolutionary & environmental sciences

For a reference copy of the document with all sections, see nature.com/documents/nr-reporting-summary-flat.pdf

Life sciences study design

All studies must disclose on these points even when the disclosure is negative.

Sample size	For in vitro experiments, sample sizes were chosen based on previous experiments that gave statistically significant results (e.g. https://doi.org/10.1038/s41586-024-08335-7, https://doi.org/10.1038/s43018-023-00513-2, https://doi.org/10.1038/s41586-022-04758-2). For in vivo experiments, sample size were calculated based on power calculations of B = 0.8 and $p < 0.05$ with the effect size from preliminary experiments and previous studies. The 3R recommendations (Replacement, reduction and refinement) were always complied.
Data exclusions	Identifying and removing outliers was made using the ROUT or Grubbs method of regression with coefficient Q = 1% or alpha = 0.05% using Graphpad Prism
Replication	All experiments were performed at least in triplicate. All attempts were successful.
Randomization	Mice were randomized before start of diet, depleting antibodies injections, tumor cell injections and sacrifice. For in vitro experiments, samples were randomly harvested and were randomized (when possible) prior acquisition.
Blinding	In vitro experiments were analyzed in a blinded manner before data collection and analysis by assigning an identifier to the sample. Mice were assigned an unique number prior to sacrifice and sample processing and data were analyzed blindly. All data were grouped in the corresponding cohorts for statistical analysis.

Reporting for specific materials, systems and methods

We require information from authors about some types of materials, experimental systems and methods used in many studies. Here, indicate whether each material, system or method listed is relevant to your study. If you are not sure if a list item applies to your research, read the appropriate section before selecting a response.

Materials & experimental systems

n/a	Involved in the study	
☐	☒	Antibodies
☐	☒	Eukaryotic cell lines
☒	☐	Palaeontology and archaeology
☐	☒	Animals and other organisms
☐	☒	Clinical data
☒	☐	Dual use research of concern
☒	☐	Plants

Methods

n/a	Involved in the study	
☒	☐	ChIP-seq
☐	☒	Flow cytometry
☒	☐	MRI-based neuroimaging

Antibodies

Antibodies used	For Western Blot experiments, the following antibodies were used: DHHC17 (1:500, Proteintech, 30022-1-AP), Streptavidin-HRP (1:5000, Thermo Scientific, 21126), Laminin beta-1 (1:1000, Proteintech, 23498-1-AP), Laminin gamma-1 (1:1000, Cell Signaling, 92921S), LCN2 (1:500, Bio-Techne AF1857), Vinculin (1:1000, Cell Signaling), Beta-Actin (1:5000 Merck A5441) primary antibodies. For secondary antibodies, HRP-linked anti-rabbit IgG (1:5000, Cell Signaling, 7074), anti-mouse IgG (1:5000, Cell Signaling, 7076) or HRP-linked anti-goat IgG (1:5000, Thermofisher,A15999) were used.<br>For fluorescent immunohistochemistry, the following antibodies were used: Ly6G (1:1000, BD Bioscience, 551459), CD8a (1:1000, Cell Signaling, 98941), DHHC17 (1:200, Proteintech, 30022-1-AP), Laminin Beta 1 (1:400, Proteintech, 23498-1-AP), LCN2 (1:200, Bio-Techne, AF1857), Epcam (1:200, Abcam, 71916), MPO (1:500, Abcam, ab9535), MPO (1:200, Bio-Techne, AF3667), citrullinated histone H3 (1:200, Cell Signaling, 97272S) and CD15 (1:100, Abcam, ab17080) as primary antibodies and HRP-conjugated anti-rabbit (Dako) or anti-mouse (Akoya) secondary antibody (both ready to use from the manufacturer).<br>For flow cytometry experiments, the following antibodies were used: CD16/CD32 (BD Bioscience, 553142, 1:25 dilution), CD45 (BD Bioscience, 550994, 1:200 dilution), Ly6G (Biolegend, S19018G clone 164507, 1A8 clone 127627 and 1A8 clone 127606, 1:400), CD11B (Biolegend, M1/70 clone 101242 and 101208, 1:400), anti-CD8a (APC, clone 53-6.7 100711, 1:200 dilution), Viability efluor780 (ThermoFisher, 65-0865-14, 1:2000 dilution), Viability efluor450 (ThermoFisher, 65-0863-14, 1:1000 dilution), Annexin V and PI (Thermo Fisher Scientific, V13241)<br>For immunoprecipitation (IP) assays, the following antibodies were used: DHHC17 (Proteintech, 30022-1-AP), Laminin Beta 1 (Proteintech, 23498-1-AP).<br>For neutrophil depletion experiments, rat anti-Ly6G (BioXCell BE0075, clone 1A8, 12.5 µg/Balbc mouse, 25-50µg for C57BL/6NCrl, in PBS) or rat IgG isotype control (BioXCell BE0089, 12.5 µg/Balbc mouse, 25-50µg for C57BL/6NCrl, in PBS) was used, together with anti-rat kappa light antibody (50µg/mouse in PBS, BioXCell, BE0122) or control (50µg/mouse in PBS, BioXCell, BE0085). For CD8a depletion experiment, anti-CD8a (BioXCell, 10mg/kg in PBS) or vehicle was used.
Validation	For Western Blot and fluorescent immunohistochemistry experiments, the antibodies were used as recommended in the data sheets and validated for immunocytofluorescence and western blot analysis and react with human and murine samples, as stated in the manufacturer's website. Additional validation was performed using knockdown/overexpression cell lines when possible.

Eukaryotic cell lines

Policy information about cell lines and Sex and Gender in Research

Cell line source(s)	Human HEK293T cells, human MCF10A, murine 4T1 and E0771 mammary gland cancer cells were obtained from ATCC. Mouse MC38 cells were obtained from Kerafast. Murine EMT6.5 mammary gland cancer cell line is a single cell clone from the EMT6 cell line available from ATCC and kindly provided by Professor Robin L. Anderson (Peter MacCallum Cancer Centre).
Authentication	None of the cell lines used were authenticated.
Mycoplasma contamination	All cell lines were regularly tested for mycoplasma contamination during culture using the MycoAlert Detection Kit (Lonza) and the MycoStrip Mycoplasma Detection Kit (InvivoGen) with negative results.
Commonly misidentified lines (See ICLAC register)	No commonly misidentified cell lines were used.

Animals and other research organisms

Policy information about studies involving animals; ARRIVE guidelines recommended for reporting animal research, and Sex and Gender in Research

Laboratory animals	Female Balb/c and C57BL/6N mice at 4 weeks age were used to start the control or high fat diets. Female Balb/c and male/female C57BL6N mice at 6-8 weeks age were used for direct cancer cells injection. All mice were not genetically engineered. All mice were housed under conventional conditions with ad libitum supply of food and water under 12h light/12h dark cycles.
Wild animals	No wild animals were used.
Reporting on sex	Findings apply to both sexes, as results apply to female breast cancer and we used both males and females for our colorectal cancer studies.
Field-collected samples	No field collected samples were used.
Ethics oversight	All animal experiments were approved by the Institutional Animal Care and Research Advisory Committee of KU Leuven, Belgium, under the ECD number P007/2020, P048/2021, P106/2023, P028/2024 regarding mouse models, house and experimental procedures. All animal experiments in this study comply with ethical regulations. For all experiments, the maximum permitted tumor volume was 1.8 cm3 and this limit was not exceeded in any experiment. For all experiments humane endpoints were applied using a scoresheet: body condition score, rough coat, abnormal posture, enlarged pupils, unsolicited vocalizations, self mutilation, restlessness or immobility, violent reactions, comatose, score 2 of mouse grimace scale, body weight loss of 20%. Mice were regularly checked on these signs and euthanized when needed.

Note that full information on the approval of the study protocol must also be provided in the manuscript.

Clinical data

Policy information about clinical studies

All manuscripts should comply with the ICMJE guidelines for publication of clinical research and a completed CONSORT checklist must be included with all submissions.

Clinical trial registration	This study is not a prospective clinical trial. The clinical data used in this study is not part of a clinical trial and thus not registered with a clinical trial registration number.
Study protocol	The study protocol for the approval from the Medical Ethics Committee of KU Leuven is available upon reasonable request.
Data collection	Data used in this study is collected during routine clinical examinations (baseline characteristics, histology of the resected specimen).
Outcomes	We did not obtain new data that is used to define outcomes. All clinical data used in this study is part of a retrospective clinical database.

Plants

Seed stocks

Report on the source of all seed stocks or other plant material used. If applicable, state the seed stock centre and catalogue number. If plant specimens were collected from the field, describe the collection location, date and sampling procedures.

Novel plant genotypes

Describe the methods by which all novel plant genotypes were produced. This includes those generated by transgenic approaches, gene editing, chemical/radiation-based mutagenesis and hybridization. For transgenic lines, describe the transformation method, the number of independent lines analyzed and the generation upon which experiments were performed. For gene-edited lines, describe the editor used, the endogenous sequence targeted for editing, the targeting guide RNA sequence (if applicable) and how the editor was applied.

Authentication

Describe any authentication procedures for each seed stock used or novel genotype generated. Describe any experiments used to assess the effect of a mutation and, where applicable, how potential secondary effects (e.g. second site T-DNA insertions, mosiacism, off-target gene editing) were examined.

Flow Cytometry

Plots

Confirm that:

☒ The axis labels state the marker and fluorochrome used (e.g. CD4-FITC).

☒ The axis scales are clearly visible. Include numbers along axes only for bottom left plot of group (a 'group' is an analysis of identical markers).

☒ All plots are contour plots with outliers or pseudocolor plots.

☒ A numerical value for number of cells or percentage (with statistics) is provided.

Methodology

Sample preparation

Livers were perfused with saline and metastases were dissected by eye using a microscope. Tissues were incubated with Liberase (Roche) (0.3 mg/mL) and DNAse1 (1 µg/mL) during 45 min at 37°C with occasional vortex. The reaction was quenched with 3% FBS:PBS + 2mM EDTA and filtered through a 70 µm cell strainer. Cell pellet was washed, incubated with ACK Lysis buffer (Merck) and transferred through a 40 µm cell strainer. Single-cells suspension was counted, resuspended and incubated with anti-Ly6G beads (Miltenyi, 130-120-337) for 10 minutes on ice. After, neutrophils were positively selected using the LS columns. These neutrophils were co-cultured for 48h with cancer cells ex vivo and subsequently analyzed using flow cytometry .

Instrument

BD FACS Diva Fortessa, BD FACS DIVA Symphony

Software

BD FACSDiva and FlowJo

Cell population abundance

For neutrophils from liver metastases: all cells (>90%), All Cells/Single cells (>97%); All cells/Single Cells/Neutrophils (>85%); All Cells/Single Cells/Neutrophils/Alive (>80%).
For the co-culture, killing analysis: All cells (>90%); All Cells/Single cells (>85%); All Cells/Single Cells/Neutrophils (>5%); All Cells/Single Cells/Cancer cells (>90%); All Cells/Single Cells/Cancer Cells/Alive (>60%).

Gating strategy

To define the starting population, FSC-A and SSC-A were used. To subsequently identify the single cell populations with FSC-H and FSC-A.
To assess purity of neutrophils after isolation, we used B530 for Ly6G-FITC and V710 for CD11B-BV711 to identify the neutrophil population (Ly6G + and CD11B +). When the percent of neutrophils is >85% we continued with the cancer cell-neutrophil co-culture experiment.
For the co-culture experiments, we used B530 for Ly6G-FITC and Y585 for CD11B-PE or V450 for Ly6G-BV421 and V710 for CD11B-BV711 to identify the neutrophil population (Ly6G + and CD11B +). The cancer cells are the double-negative population. To assess the alive/dead cancer cells, the cancer cells were taken separately and the R780 was assessed for the Viability dye eFluor 780. To specifically assess apoptotic and dying cells in the co-cultures, the cells were stained with Annexin V and priorium ionide (PI).
Details on gating strategy is provided in Supplementary Data Figure 1.

☒ Tick this box to confirm that a figure exemplifying the gating strategy is provided in the Supplementary Information.
