# Supplementary material for: Palmitate promotes liver metastases by decreasing neutrophil antitumour behaviour
Source: Nat Metab. Author manuscript; Available in PMC 2026 Oct 17. (PMC7619559; doi:10.1038/s42255-026-01582-0)
Supplement: Supplementary file 2 [file EMS217595-supplement-Supplementary_Data_Figure_1.pdf]

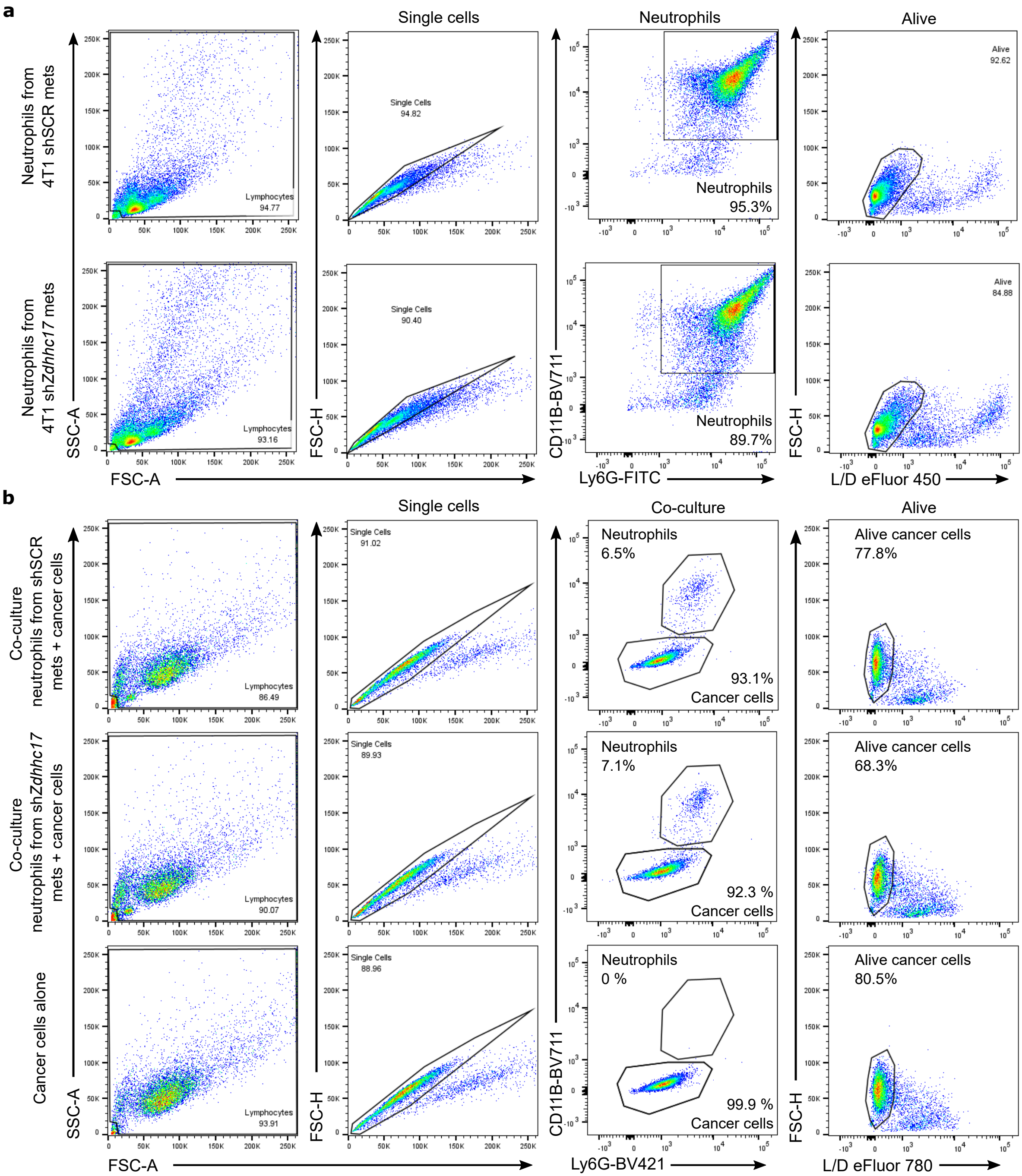

Supplementary Data Figure 1 Gating strategy for isolated neutrophils from liver metastases and the neutrophil-cancer cell killing assay

a. Gating strategy for neutrophils from liver metastases after isolation with anti-Ly6G beads. Based on this FACS analysis, the isolation efficacy of neutrophils from liver metastases was assessed (89% and 95%)

b. Gating strategy for killling assays of neutrophils from liver metastases or blood with cancer cells ex vivo
